# Iridium‐Catalyzed Ionic Hydrogenation of Multi‐Aza Heterocycles

**DOI:** 10.1002/anie.4341513

**Published:** 2026-07-13

**Authors:** Arthur Despois, Nicolai Cramer

**Affiliations:** ^1^ Institute of Chemical Sciences and Engineering (ISIC) Lausanne Switzerland

**Keywords:** homogeneous catalysis, hydrogenation, iridium, nitrogen heterocycles

## Abstract

Nitrogen‐containing‐heterocycles are a cornerstone of biologically active molecules, natural products, and pharmaceuticals. Saturated heterocycles can lead to improved clinical success of drugs over their aromatic counterpart and sp^3^‐rich three‐dimensional molecules cover a much broader and complex chemical space. In this respect, increasing the number of nitrogen atoms in saturated heterocycles open up a wide range of isomeric structures with defined conformations. However, hydrogenation technology of complex heterocycles as efficient access strategy to these entities is still not general and straightforward. Herein, we report an ionic iridium‐catalyzed hydrogenation platform of diverse multi‐aza arenes to (partially)saturated heteroarenes. With a readily available iridium(III)‐complex, we devised a robust system for the selective hydrogenation of 14 different heteroaromatic cores with a unified approach, mild reaction conditions preserving typical reduction‐sensitive functional groups such as halides. The hydrogenation operates with low loadings of 0.5 mol%, is amenable to multigram scales without adaption. For instance, all three isomeric diazines cores were reduced to useful piperazines, tetrahydropyrimidines as well as elusive piperidazines with its fragile N─N bonds. Additional heterocycles containing up to four nitrogen atoms comprise isomeric [6,6]‐naphthyridines, pyridopyrazines, pyrazolopyridines, pyrazolopyrimidines, and triazolopyrazines.

## Graphical abstract

Saturated nitrogen‐containing heterocycles are ubiquitous in bioactive molecules and are thus attractive synthetic targets. A readily accessible cyclometalated iridium(III) complex reduces 14 different aromatic aza heterocycles to (partially)saturated multi aza‐heterocycles enlarging accessible chemical space while displaying high tolerance toward reduction‐sensitive functional groups.

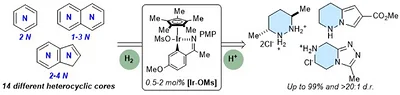

## 1 Introduction

**FIGURE 1:**
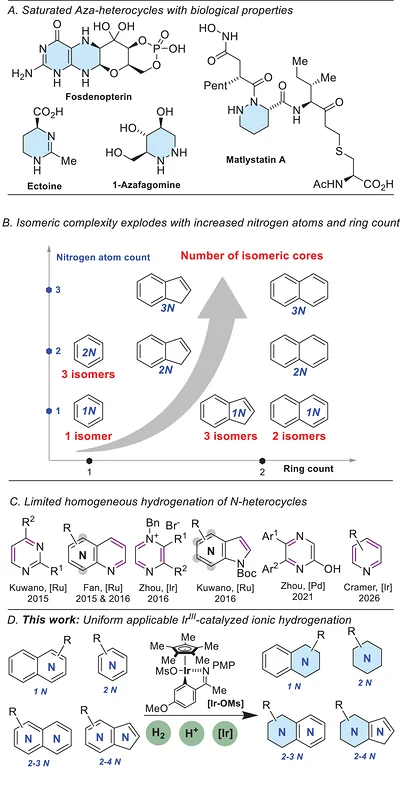
Pharmaceutical relevance of (partially)saturated multi aza‐heterocycles and their access via homogeneous hydrogenation. (A) Selected examples of relevant drug molecules with multi‐heteroatom (partially) saturated heterocycles. (B) Increase of isomeric complexity by the addition of additional nitrogen atoms and rings. (C) Reported homogeneous hydrogenation of nitrogen‐heterocycles are scattered with narrow windows on the available chemical space. (D) Uniformly applicable iridium(III)‐catalyzed ionic hydrogenation platform for the reduction of diverse multi‐aza arenes with broad functional group tolerance.

Nitrogen containing heterocycles are omnipresent in medicinal chemistry with more than 82% of FDA‐approved drugs containing aza‐heterocycles in the last decade [1, 2]. The most prevalent nitrogen‐containing heterocycles are 5‐ and 6‐membered monocyclic heterocycles—respectively pyridine, piperidine, pyrrolidine, piperazine, and pyrimidine. However, the proportion of fused heterocycles often containing multiple nitrogen atoms is substantially increasing, doubling from a long‐term 10% until 2013 to 22% in the 2013–2023 decade, suggesting realization of a strong underlying potential. Saturation of flat only aromatic motifs in drugs have long been correlated to improved clinical success rates, allowing more sophisticated and diverse 3‐dimensional design with minimal molecular weight increase (Figure 1A) [3, 4, 5]. Thus, deraromative hydrogenation is an efficient strategy allowing to construct challenging saturated or partially saturated heterocycles from abundant and easily‐accessible aromatic heterocycles [6, 7, 8, 9]. Starting from a simple heterocyclic core like pyridine, the addition of an additional nitrogen atom and/or an additional ring—both important for medicinal chemistry needs—has a dramatic effect on the possible number of isomers (Figure 1B). The addition of a second nitrogen atom to a pyridine ring triples the amount of possible isomeric cores, while fusing a second ring provides for five different isomeric cores. Incorporation of a second nitrogen atom to a fused bicyclic system dramatically increases the number of possible isomeric aromatic cores. However, the developed hydrogenation methods have barely scratched the surface of these multi‐hetero atom heterocycles (Figure 1C). While hydrogenations of monocyclic pyridines have been reported [9, 10, 11], related reactions with isomeric pyrazines [12, 13], and pyrimidines [14] remain understudied, and pyridazine hydrogenation are virtually elusive [15, 16]. In contrast, the partial reduction of quinoline and isoquinolines as examples for fused heterocycles with single nitrogen atoms that are established and can be linked to their lower aromaticity energy barrier that needs to be overcome [17]. However, hydrogenation of [6,6]‐ or [6,5]‐fused ring system bearing two or more nitrogen atoms are very scarce. For instance, naphthyridines and their corresponding tetrahydronaphthyridines are relevant in medicinal chemistry context [18], but only few examples of the corresponding hydrogenation are reported under either ruthenium of palladium catalysis [19, 20, 21]. Besides the rather harsh heterogeneous Pd/C or PtO_2_ hydrogenation system, other complex heterocycles like pyrazolo[1,5‐*a*]pyrimidines [22], pyrido[2,3‐b]pyrazine [23], and aza‐indoles [24] received little or no attention.

Inspired by our previously developed ionic iridium catalyzed hydrogenation of pyridines [11], we endeavored to explore a generalized hydrogenation technology applicable for a broad variety of nitrogen‐containing heterocycles and fused heterocycles (Figure 1D). With readily accessible cyclometalated iridium complex **[Ir‐OMs]**, we successfully reduced 14 different heterocyclic and fused heterocyclic aromatic cores containing from one to four nitrogen atoms. Under mild reactions conditions, monocyclic, [6,6]‐ and [6,5]‐fused aromatic ring systems were selectively hydrogenated providing access to their saturated analogues whilst preserving reduction‐sensitive C─Br, C─I or N─N bonds that are lost under typical heterogenous catalysis conditions [25].

### 1.1 Results and Discussion

The hydrogenation of diaza‐heterocycles was investigated and optimized with methyl pyrazine‐2‐carboxylate **1** (Table 1). Exposure to hydrogen (50 bar) and trifluoroacetic acid in the presence of 2 mol% of **[Ir‐OMs]** in methanol provided corresponding piperazine salt **2** in 92% yield (entry 1). The hydrogenation reaction could be run at medium or higher dilution without affecting the yield of **2** (entries 2 and 3). The reaction solvent could be exchanged to isopropanol or MeTHF with similar yields. However, methanol remained preferential providing an overall better substrate solubility (entries 4 and 5). The amount of TFA could be reduced to one equivalent maintaining an excellent yield (entry 6). Nevertheless, superstoichiometric amounts of acids were maintained to ensure complete protonation of any basic nitrogen atom of the substrate. The catalyst loading could be reduced to 1 mol% while conserving the same reactivity. The reduction could be scaled‐up 25‐fold affording 2.03 g (94% yield) of **2** using 0.5 mol% of **[Ir‐OMs]** (entries 7 and 8). At a lower hydrogen pressure of 10 bar, no changes in kinetics or yield were observed (entry 9).

**TABLE 1:** Optimization of the hydrogenation of pyrazine 1.^a^

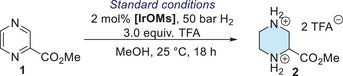					
Entry	TFA (equiv.)	Solvent	Concentration (M)	[IrOMs] (mol%)	Yield 2 (%)
1	3	MeOH	1	2	92^b^
2	3	MeOH	0.5	2	93
3	3	MeOH	2	2	90
4	3	*i*PrOH	1	2	91
5	3	MeTHF	1	2	73
6	1	MeOH	1	2	90
7	3	MeOH	1	1	90
8^b^	3	MeOH	1	0.5	94%^c^ *[2.03 g]*
9^d^	3	MeOH	1	2	94

^a^ Conditions: 0.4 mmol **1**, 8 µmol [**IrOMs**], 1.2 mmol TFA, 50 bar H_2_ in MeOH (1 M) at 25°C for 18 h; NMR yield with 1,3,5‐trimethoxybenzene as internal standard.

^b^ 10 mmol **1**, 50 µmol [**IrOMs**], 30 mmol TFA in 10 mL MeOH.

^c^ Isolated yield.

^d^ 10 bar H_2_.

**SCHEME 1:**
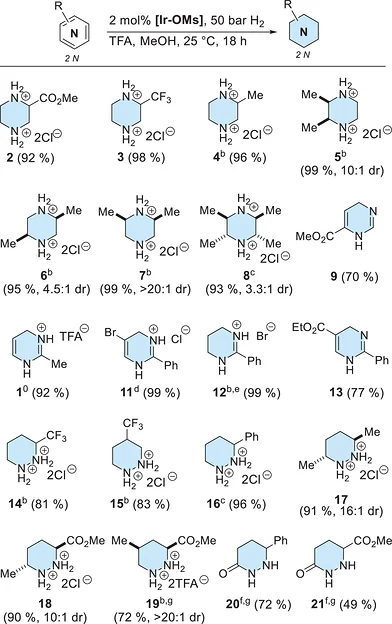
Hydrogenation of monocyclic diazines. ^a^ Conditions: 0.4 mmol heterocycle, 8 µmol [**IrOMs**], 1.2 mmol TFA, 50 bar H_2_ in MeOH (1 M) at 25°C for 18 h; Isolated yield; ^b^ at 60°C; ^c^ at 80°C; ^d^ for 1 h; ^e^ from 5‐bromo‐2‐phenylpyrimidine; ^f^ from 3‐hydroxyl‐substituted pyridazines in THF; ^g^ With 4Å molecular sieves in THF.

We then investigated the hydrogenation of monocyclic diazines (Scheme 1). Electron‐poor pyrazines bearing either a methyl ester (**2**) or trifluoromethyl group (**3**) were smoothly hydrogenated at room temperature to the corresponding piperazine in high yields. 2‐Methyl pyrazine was amenable to hydrogenation at 60°C, affording piperazine **3** in 96% yield. Full permutation of three methyl groups on the pyrazine core (2,3‐; 2,5‐, and 2,6‐) selectively provided the corresponding *cis*‐piperazines **5**‐**7** in excellent yield and moderate‐to‐excellent diastereoselectivities. Tetra‐methyl pyrazine was smoothly hydrogenated to tetramethyl piperazine **8** in 93 % yield as a 3.3:1 ratio of diastereomers. As second class of diaza heterocycles, pyrimidines were found to be amenable to partial hydrogenation. Mono‐substituted pyrimidines bearing a methyl ester in the 4‐position or a methyl group in the 2‐ position gave rise to the corresponding 1,4‐dihydropyrimidines in high yields (**9** and **10**). 2,5‐Disubstituted pyrimidines were converted to the corresponding dihydropyrimidines, for example, leading to synthetically exploitable vinyl bromide **11** or unsaturated ester **13**. Notably, conducting the hydrogenation of the bromo‐pyrimidine at 60°C for 18 h instead of 25°C for 1 h led to debromination and further reduction forming amidinium bromide **12** in virtually quantitative yield. We next turned our attention to the underexplored hydrogenation of pyridazines. Intriguingly, pyridazines bearing either electron‐donating or withdrawing groups responded well providing corresponding piperidazines **14**–**16** as the major reaction products. Noteworthy, the hydrogenation proceeds without a reductive cleavage of the weak N─N bond. 2,5‐Dimethyl pyridazine was selectively reduced to *trans*‐piperidazine **17** in 91% yield and 16:1 dr. Replacement of one methyl group by a methyl ester provided product **18** in 90% and 10:1 dr. Isomeric 3,5‐substituted pyridazine was reduced to predominantly *cis*‐piperidazine **19**. Hydroxy‐pyridazine substrates selectively formed the corresponding six‐membered lactams **20** and **21** in good yields.

**SCHEME 2:**
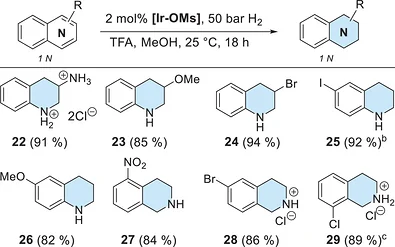
Hydrogenation of quinolines and isoquinolines ^a^ Conditions: 0.4 mmol quinoline or isoquinoline, 8 µmol [**IrOMs**], 1.2 mmol TFA, 50 bar H_2_ in MeOH (1 M) at 25°C for 18 h; Isolated yield; ^b^ 1 h instead of 18 h; ^c^ at 60°C.

While the hydrogenation of the heteroaryl portion of quinolines and isoquinolines is established [17], the functional group tolerance especially of sensitive reducible groups require improvements. We thus tested several quinolines and isoquinolines with our catalytic system (Scheme 2). In this respect, electron‐donating groups on the pyridyl portion were well tolerated and the 3‐amino‐ and 3‐methoxy tetrahydroquinolines were formed obtained in very good yields (**22**, **23**). 3‐Bromo tetrahydroquinoline **24** was obtained in 94% yield, showing the remarkable tolerance of for halides, even placed on the reduced ring. In analogy, 6‐iodoquinoline was reduced to **25** in 92% yield in 1 h reaction time, and 6‐methoxyquinoline **26** was obtained in 82% yield. Tetrahydroisoquinolines **27‐29** bearing either a nitro‐, bromo‐, or a chloro‐ substituent on the remaining arene ring were obtained in high yields, once again without any hydrogenation of the reduction‐sensitive functional groups.

**SCHEME 3:**
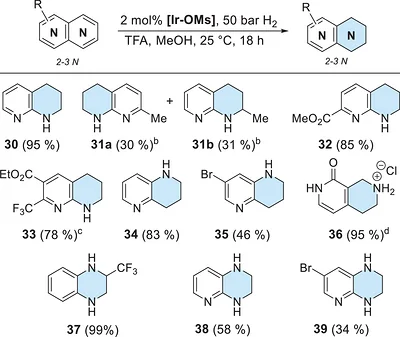
Hydrogenation of di‐ and tri‐aza [6, 6]‐fused heteroarenes ^a^ Conditions: 0.4 mmol heterocycle, 8 µmol [**IrOMs**], 1.2 mmol TFA, 50 bar H_2_ in MeOH (1 M) at 25°C for 18 h; Isolated yield; ^b^ from 2‐methyl‐1,8‐naphthyridine; ^c^ for 1 h; ^d^ From 2,7‐naphthyridin‐1‐ol.

We then turned our attention to the hydrogenation of more sophisticated [6,6]‐fused bicyclic heteroarenes containing two or three nitrogen atoms (Scheme 3). Pleasingly, 1,8‐naphthyridines were found to be very reactive and smoothly reduced at 25°C with our standard reaction conditions. The parent compound was reduced to 1,2,3,4‐tetrahydro‐1,8‐naphthyridine **30** with one remaining pyridine core in 95% yield. A methyl group in the 2‐position of 1,8‐naphthyridine did not cause any preferential bias for a particular pyridine ring, forming regioisomeric products **31a** and **31b** in essentially the same amount. In contrast, the installation of an electronic bias (methyl ester) triggered the selective reduction of the non‐substituted pyridine unit forming **32** in 85% yield. In analogy, naphthyridine substrate bearing a 2‐CF_3_ and 3‐CO_2_Et was reduced with the same preference to **33**. However, a short reaction time of 1 h was necessary to avoid further reduction and side products. Regioisomeric 1,5‐naphthtyridines were also converted to their semi‐saturated congeners. The parent heterocycle cleanly reduced to pyridine **34**. 3‐Bromo‐1,5‐naphthyridine selectively yielded 3‐bromo pyridine derivative **35**, again evidencing the subtle selectivity effects at play. Isomeric 1‐hydroxy‐2,7‐naphthyridine reduced to give the piperidine‐pyridone fused product **36** in excellent yield. As heterocycles having the two nitrogen atoms on the same core, quinoxalines such as 2‐trifluoromethylquinoxaline underwent smooth hydrogenation of the heterocyclic core yielding tetrahydroquinoxaline **37** in virtually quantitative yield. As an example for a complex triaza heterocycle, pyrido[2,3‐b]pyrazine having two potentially reducible heterocyclic cores were checked with our catalytic system. The parent compound was selectively hydrogenated to compound **38** with an intact pyridine ring in good yield. An analog with 3‐bromo substitution displayed the same chemoselectivity favoring reduction of the diaza‐containing core to deliver 3‐bromo pyridine derivative **39** while conserving the sensitive bromide.

**SCHEME 4:**
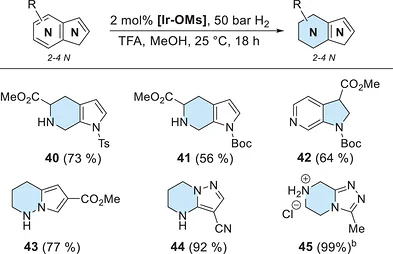
Hydrogenation of di‐, tri‐, and tetra‐aza [6, 5]‐fused heteroarenes. ^a^ Conditions: 0.4 mmol heterocycle, 8 µmol [IrOMs], 1.2 mmol TFA, 50 bar H_2_ in MeOH (1 M) at 25°C for 18 h; Isolated yield; ^b^ 6 equivalents of TFA at 60°C.

We further expanded the hydrogenation technology to a range of [6, 5]‐fused azaheterocycles with two to four nitrogen atoms (Scheme 4). In this respect, 6‐azaindoles were reduced under our standard reaction conditions. When the pyridine portion was substituted with an electron‐withdrawing methyl ester, the major product was the fully reduced pyridine core with an intact pyrrole unit. *N‐*Tosyl pyrrole **40** was formed in 73% yield. Related *N*‐Boc protected pyrrole **41** was formed without no detectable cleavage of the Boc group in the acidic reaction media at 25°C. Curiously, the reduction selectivity for the pyridine ring is contrasting the selectivity compared to known catalytic systems [24, 26] representing the first instance of homogeneous hydrogenation with such preferred selectivity. Interestingly, installation of an electron‐withdrawing methyl ester on the pyrrole unit prompted a selective reduction of the pyrrole core, yielding pyridine **42** in 64% yield. The selectivity could be explained by the presence of an electron‐withdrawing group on the pyridine ring in **41** enabling hydrogenation, while **42** has the methyl ester on the pyrrole ring, removing the needed electron‐withdrawing activation of the pyridine and leading to reduction of a β‐unsaturated ester. The hydrogenation could be further extended to pyrrolopyridazine, enabling access to the corresponding piperidazine‐type product **43** in 77% yield with an intact N─N bond. Complex nitrogen‐heteroarenes bearing three or four nitrogen atoms were found as well reducible. For instance, cyano‐substituted pyrrazolopyrimidine was reduced to corresponding aminopyrazole **44** in excellent yield. Similarly, a methyl substituted triazolopyrazine having four nitrogen atoms underwent a selective reduction of the pyrazine core providing quantitively 1,2,4‐triazole **45** at 60°C.

## 2 Conclusion

In summary, we have developed a generally applicable ionic hydrogenation platform leveraging iridium(III) catalysis for the hydrogenation of diverse multi‐aza arenes to (partially)saturated heteroarenes which are of increasingly high relevance in pharmaceuticals. With a readily available iridium(III)‐complex, we devised a robust system for the selective hydrogenation of 14 different heteroaromatic cores with a unified approach under mildly acidic reaction conditions, which can undoubtedly be expended further. The hydrogenation operates with low loadings of 0.5 mol%, is amenable to multigram scales without adaption, and relatively fast reaction times, showing potential for further up‐scale development and optimization. This catalytic system unlocks new reactivity such as providing straightforward access to unexplored piperidazines as well as exploitable regioselectivities—all the while preserving reduction‐sensitive functional groups.

## Author Contributions

**Arthur Despois**: methodology, conceptualization, investigation, writing – original draft, writing – review and editing. **Nicolai Cramer**: supervision, conceptualization, methodology, writing – original draft, writing – review and editing, funding acquisition.

## Conflicts of Interest

The authors declare no conflicts of interest.

## Supporting information



The authors have cited additional references within the Supporting Information [25, 27, 28, 29, 30, 31, 32, 33, 34, 35, 36, 37, 38, 39, 40, 41]. **Supporting File**: anie73533‐sup‐0001‐SuppMat.pdf.

## Data Availability

The data that supports the findings of this study are available in the supplementary material of this article.
