# Supplementary material for: Iridium‐Catalyzed Ionic Hydrogenation of Multi‐Aza Heterocycles
Source: Angew Chem Int Ed Engl. 2026 Jul 13;65(35):e4341513. doi: 10.1002/anie.4341513 (PMC13502548; doi:10.1002/anie.4341513)
Supplement: Supplementary file 1 [file ANIE-65-e4341513-s001.pdf]

Iridium-Catalyzed Ionic Hydrogenation of Multi-aza Heterocycles

Supplementary Information

Authors: Arthur Despois[1], Nicolai Cramer[1]*

Affiliations:

1. Laboratory of Asymmetric Catalysis and Synthesis, Institute of Chemical Sciences and Engineering, Ecole Polytechnique Fédérale de Lausanne (EPFL), 1015 Lausanne, Switzerland.

*Correspondence to: nicolai.cramer@epfl.ch

Table of Contents

Materials and methods **3**
General Procedure for the hydrogenation of heterocycles – GP **4**
Characterization of new compounds **5**
  Substates synthesis 5
  Pyrazine hydrogenation 6
  Pyrimidines hydrogenation 8
  Pyridazines hydrogenation 9
  [6,6]-fused heteroarene hydrogenation 11
  [6,5]-fused heteroarene hydrogenation 15
NMR Spectra **17**
  Substates synthesis 17
  Pyrazines hydrogenation 20
  Pyrimidines hydrogenation 28
  Pyridazines hydrogenation 33
  [6,6]-fused heteroarenes hydrogenation 41
  [6,5]-fused heteroarenes hydrogenation 52
Supplementary references **58**

Materials and methods

All reactions were carried out under inert atmosphere in oven-dried glassware with magnetic stirring, unless otherwise indicated. Hydrogenations were performed in a stainless steel Berghof BR- 100/200 high-pressure autoclave. Iridium chloride **[Ir-Cl]** and mesylate **[Ir-OMs]** were synthesized according to known literature.[1]

The following substrates were synthetized according to known literature: 3-Phenylpyridazine,[2] 2methyl 1,8-naphthyridine-2-carboxylate.[3] All other substates and reagents were used as obtained from the suppliers unless otherwise noted.

Dry diethyl ether (Et_2O), dichloromethane (DCM), toluene and tetrahydrofuran (THF) were purified by an Innovative Technology Solvent Delivery System. Ethyl acetate (EtOAc), dichloromethane (DCM) and pentane for filtration, *trans*fers, chromatography, and recrystallizations were purchased from commercial sources and distilled before use. MeOH, *i*PrOH and THF were HPLC-grade solvents used as received. Column chromatography was performed with Silicycle silica gel 60 (40-63 μm grade). Analytical thin-layer chromatography was performed with commercial glass plates coated with 0.25mm silica gel (E. Merck, Kieselgel 60 F254). R*f* values reported were measured using a 5 x 2 cm plate. Compounds were visualized by UV-light at 254 nm and by dipping the plates in an ethanolic vanillin/sulfuric acid solution, an aqueous potassium permanganate solution or a butanolic ninhydrin/acetic acid solution, followed by heating.

Proton nuclear magnetic resonance (^{1}H NMR), proton decoupled carbon-13 nuclear magnetic resonance (^{13}C{^{1}H} NMR), fluorine-19 nuclear magnetic resonance (^{19}F NMR) and proton decoupled fluorine-19 nuclear magnetic resonance (^{19}F{^{1}H} NMR) spectra were recorded on a Bruker AVANCEIII-400 spectrometer (400 MHz, ^{1}H; 101 MHz, ^{13}C; 376 MHz, ^{19}F; 162 MHz, ^{31}P). Chemical shifts are reported in parts per million (ppm). Spectra are referenced to residual chloroform (δ = 7.26 ppm, ^{1}H; 77.16 ppm, ^{13}C), residual benzene (δ = 7.16 ppm, ^{1}H; 128.06 ppm, ^{13}C), according to the literature.[4] All splitting patterns are designated as s, singlet; d, doublet; t, triplet; q, quartet; hept, heptuplet; m, multiplet, br (broad). All NMR data were recorded at 298 K unless otherwise indicated.

For sample measured in D_2O: ^{1}H Chemical shifts were referenced to TMS using the signals of the residual protons of the deuterated solvent (δ^1H = 4.79 (D_2O)) as secondary reference. NMR Spectra of heteronuclei were referenced using the Ξ-scale with TMS (Ξ = 25.145020 MHz, ^{13}C), 85 % H_3PO_4 (Ξ = 40.480747 MHz, ^{31}P) and CCl_3F (Ξ = 94.094011 MHz, ^{19}F) as secondary references.[5]

Infrared (IR) data were recorded on an Alpha-P Bruker FT-IR spectrometer. Absorbance frequencies are reported in reciprocal centimeters (cm^{-1}). HRMS data were acquired on an Agilent LC-MS TOF (Multimode: ESI + APCI) or on a LTQ Orbitrap FTMS instrument (LTQ Orbitrap Elite FTMS, Thermo Scientific, Bremen, Germany) equipped with an Ion Max APPI ionization source with a VUV Kr lamp (Syagen, CA, USA). The sample was diluted in DCM and directly infused into the mass spectrometer. FT-MS spectra were recorded in the 100-1000 m/z range in the positive mode with a resolution set to 120,000). The sample was diluted in DCM and directly infused into the mass spectrometer. FT-MS spectra were recorded in the 100-1000 *m/z* range in the positive mode with a resolution set to 120,000. Melting points were measured on a Büchi B-540 and are uncorrected. HRMS analyses were performed by Dr. L. Menin team at the mass spectroscopy platform of ISIC at the EPF Lausanne.

General Procedure for the hydrogenation of heterocycles – GP

Without any precaution from air or moisture, the substrate (0.40 mmol, 1.0 equiv.) was added to a glass vial (1.5 mL). The catalyst (5.4 mg, 8.0 µmol, 0.02 equiv.) dissolved in the corresponding solvent (1M, 0.4 mL) was added, followed by trifluoroacetic acid (1.2 mmol, 92 µL, 3.0 equiv.) using a micropipette. A stir bar was added, the vial was closed with an adapted PTFE screw cap with inner septum, pierced with a needle (21G) and placed in a high-pressure autoclave, purged with H_2 (3 x 5 bar), pressurized to 50 bar H_2 and stirred for 18 h at 25 °C (unless specified otherwise), 1000 rpm. When heating, the stirring plate was set to the desired temperature (external sensor). After 18h the heating was turned off, the autoclave was left to cool down until it reached < 40 °C, (usually ≈30 min) and the pressure was carefully released.

General Procedure A – Aqueous work-up to access salts – GPA

The reaction mixture was poured in a 50 mL separatory funnel, diluted with 10 mL water and washed with 2 x 10 mL of DCM. The aqueous layer was basified using ≈1 mL saturated aqueous K_2CO_3 and extracted with 3 x 20 mL of DCM. The combined organic layers were dried over $MgSO_4$, filtered-off, then HCl (2 M in H_2O, 0.40 mL, 0.80 mmol, 2.0 equiv/nitrogen, e.g. 0.80 mL for piperazines) was added and the solvent was removed under reduced pressure to give the crude product as an HCl salt.

General Procedure B – Aqueous work-up to access free bases – GPB

The reaction mixture was poured in a 50 mL separatory funnel, diluted with 10 mL water and washed with 2 x 10 mL of DCM. The aqueous layer was basified using ≈1 mL saturated aqueous K_2CO_3 and extracted with 3 x 20 mL of DCM. The combined organic layers were dried over $MgSO_4$, filtered-off, and the solvent was removed under reduced pressure to give the crude product which was then purified by silica-gel chromatography if needed.

General Procedure C – Lyophilization – GPC

The reaction mixture was poured in a 50 mL separatory funnel, diluted with 10 mL water and washed with 2 x 10 mL of DCM. The aqueous layer was acidified with HCl (2 M in H_2O, 0.40 mL, 0.80 mmol, 2.0 equiv/nitrogen, e.g. 0.80 mL for piperazines), frozen in liquid nitrogen and lyophilized to give the crude product as an HCl salt.

General Procedure D – Basification then column chromatography – GPD

The reaction mixture was directly basified with excess NEt_3 (0.2 mL) and concentrated under reduced pressure, then purified by column chromatography.

Scale-up procedure for the gram-scale hydrogenation of methyl pyrazine-2-carboxylate (1).

Without any precaution from air or moisture, methyl pyrazine-2-carboxylate **1** (1.38 g, 10.0 mmol) was added to a 20 mL microwave vial. The catalyst (34 mg, 0.05 mmol, 0.50 mol%) dissolved in MeOH (1 M, 10 mL) was added, followed by trifluoroacetic acid (2.3 mL, 30 mmol, 3.0 equiv.). A stir bar was added, the vial was sealed, pierced with a needle (21G) and placed in a high-pressure autoclave, purged with H_2 (3 x 5 bar), pressurized to 50 bar H_2 and stirred for 18 h at 25 °C, 1000 rpm. After 18h the pressure was carefully released, HCl (2 M in Et_2O, 25 mL, 50 mmol) was added and the crude mixture was concentrated under reduced pressure and co-evaporated with MeOH (2 x 25mL). The resulting yellow solid was triturated with DCM to give an off-white powder (2.03 g, 94 %).

Characterization of new compounds

***Note**: In many cases the NMR solvent is D_2O, hence the ammonium protons NH_2^+ are exchanged and undetectable, as well as other heteroatom-bound protons.*

Substates synthesis

Methyl 1-tosyl-1*H*-pyrrolo[2,3-*c*]pyridine-5-carboxylate (SM-40)

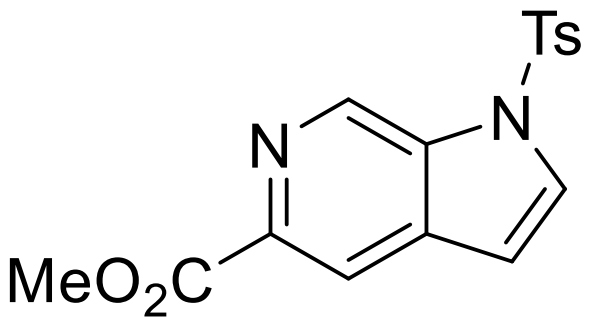

To a solution of methyl 1*H*-pyrrolo[2,3-*c*]pyridine-5-carboxylate (0.50 g, 2.8 mmol, 1.0 equiv.) in MeCN (10 mL) was added DMAP (0.52 g, 4.3 mmol, 1.5 equiv.) followed by TsCl (1.1 g, 5.7 mmol, 2.0 equiv.) and the reaction mixture was stirred at 25 °C for 18 h. The reaction mixture was partitioned between EtOAc and saturated aqueous $NaHCO_3$, the aqueous layer was extracted three times with EtOAc. The combined organic layers were dried over $MgSO_4$, filtered off and concentrated under reduced pressure. The crude product was purified by column chromatography (2% MeOH in DCM, Rf = 0.8) to give the product as a white solid (0.74 g, 79%)

^{1}H NMR (400 MHz, $CDCl_3$) δ 9.37 (t, *J* = 0.9 Hz, 1H), 8.38 (d, *J* = 1.0 Hz, 1H), 7.84 – 7.79 (m, 2H), 7.77 (d, *J* = 3.6 Hz, 1H), 7.28 – 7.24 (m, 2H), 6.78 (dd, *J* = 3.6, 0.8 Hz, 1H), 4.01 (s, 3H), 2.36 (s, 3H). **^{13}C NMR** (101 MHz, $CDCl_3$) δ 166.1, 146.3, 141.3, 136.8, 135.7, 134.6, 133.1, 130.7, 130.5, 127.2, 118.9, 108.5, 53.0, 21.8.

1-(*Tert*-butyl) 5-methyl 1*H*-pyrrolo[2,3-*c*]pyridine-1,5-dicarboxylate (SM-41)

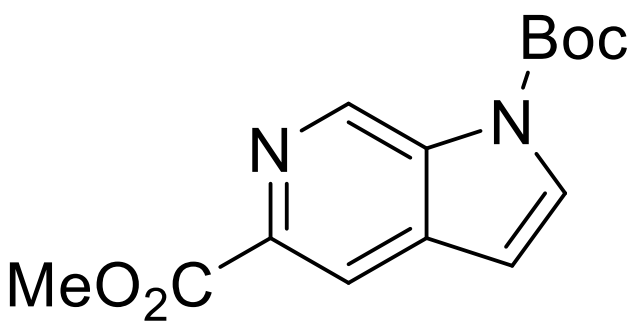

To a solution of methyl 1*H*-pyrrolo[2,3-*c*]pyridine-5-carboxylate (0.30 g, 1.7 mmol, 1.0 equiv.) in DCM (4 mL) was added NEt_3 (0.48 mL, 3.4 mmol, 2 equiv.) followed by Boc_2O (0.44 g, 2.0 mmol, 1.2 equiv.) and catalytic amount of DMAP and the reaction mixture was stirred at 25 °C for 18 h. The reaction mixture was partitioned between DCM and 10% (w/v) citric acid, the aqueous layer was extracted three times with DCM. The combined organic layers were dried over $MgSO_4$, filtered off and concentrated under reduced pressure. The crude product was purified by column chromatography (60% EtOAc in pentane, Rf = 0.5) to give the product as a white solid (0.745 g, 96%).

^{1}H NMR (400 MHz, $CDCl_3$) δ 9.46 (s, 1H), 8.42 (d, *J* = 1.0 Hz, 1H), 7.84 (d, *J* = 3.6 Hz, 1H), 6.71 (dd, *J* = 3.7, 0.7 Hz, 1H), 4.03 (s, 3H), 1.71 (s, 9H). **^{13}C NMR** (101 MHz, $CDCl_3$) δ 166.5, 148.7, 140.6, 137.3, 136.5, 133.4, 130.4, 118.6, 107.1, 85.9, 52.9, 28.3.

1-(*Tert*-butyl) 3-methyl 1*H*-pyrrolo[2,3-*c*]pyridine-1,3-dicarboxylate (SM-42)

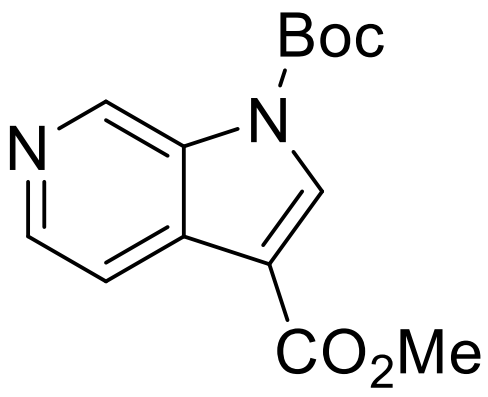

To a solution of methyl 1*H*-pyrrolo[2,3-*c*]pyridine-3-carboxylate (0.30 g, 1.7 mmol, 1.0 equiv.) in DCM (4 mL) was added NEt_3 (0.48 mL, 3.4 mmol, 2 equiv.) followed by Boc_2O (0.44 g, 2.0 mmol, 1.2 equiv.) and catalytic amount of DMAP and the reaction mixture was stirred at 25 °C for 18 h. The reaction mixture was directly purified by column chromatography (60% EtOAc in pentane, Rf = 0.4) to give the product as a white solid (0.45 g, 91%).

^{1}H NMR (400 MHz, $CDCl_3$) δ 9.44 (s, 1H), 8.52 (d, *J* = 5.4 Hz, 1H), 8.38 (s, 1H), 8.04 (dd, *J* = 5.4, 1.1 Hz, 1H), 3.96 (s, 3H), 1.71 (s, 9H). **^{13}C NMR** (101 MHz, $CDCl_3$) δ 163.8, 148.2, 143.3, 137.7, 134.6, 133.2, 132.4, 116.0, 111.7, 86.4, 51.8, 28.1.

Pyrazine hydrogenation

Methyl piperazine-2-carboxylate dihydrochloride (2)

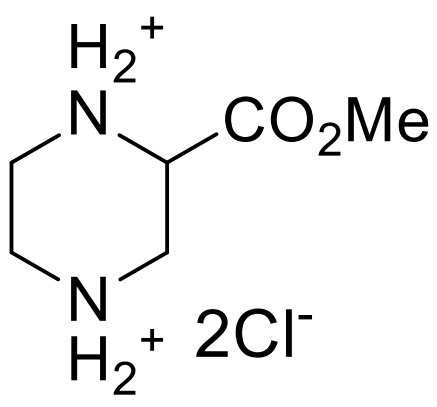

Compound **2** was prepared from methyl pyrazine-2-carboxylate **1** (55.3 mg, 0.40 mmol) according to **GPA** at 25 °C. Yellow solid (79.9 mg, 92%).

^{1}H NMR (400 MHz, D_2O) δ 4.56 (dd, *J* = 10.4, 3.9 Hz, 1H), 3.94 (dd, *J* = 14.3, 3.2 Hz, 1H), 3.84 (s, 3H), 3.75 – 3.67 (m, 1H), 3.67 – 3.59 (m, 1H), 3.57 – 3.29 (m, 3H). **^{13}C NMR** (101 MHz, D_2O) δ 168.3, 57.1, 55.2, 44.0, 42.6, 42.4. **FTIR** (cm^{-1}): 3371, 2912, 2594, 2454, 1745, 1587, 1574, 1442, 1394, 1371, 1233, 1179, 1057, 929, 891. **HRMS** (Sicrit plasma/LTQ-Orbitrap) m/z: $[M - 2HCl + H]^+$ Calculated for $[C_6H_{13}N_2O_2]^+$ 145.0972; Found 145.0972. **m.p**. 147-149°C.

2-(Trifluoromethyl)piperazine dihydrochloride (3)

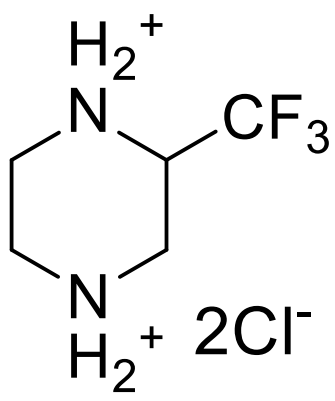

Compound **3** was prepared from 2-(trifluoromethyl)pyrazine (59.2 mg, 0.40 mmol) according to **GPA** at 25 °C. Off-white solid (89.0 mg, 98%).

^{1}H NMR (400 MHz, D_2O) δ 4.18 – 4.03 (m, 1H), 3.77 (ddd, *J* = 13.2, 3.3, 1.3 Hz, 1H), 3.61 – 3.42 (m, 2H), 3.33 – 3.19 (m, 3H). **^{13}C NMR** (101 MHz, D_2O) δ 127.1 (q, *J* = 280.5 Hz), 55.7 (q, *J* = 31.4 Hz), 44.6, 43.3 (q, *J* = 2.8 Hz), 43.2. **^{19}F NMR** (376 MHz, D_2O) δ -75.18. **FTIR** (cm^{-1}): 2977, 2955, 2918, 1721, 1703, 1498, 1477, 1376, 1332, 1280, 1158, 1094, 1021, 986. **HRMS** (ESI/QTOF) m/z: $[M - 2HCl + H]^+$ Calculated for $[C_5H_{10}F_3N_2]^+$ 155.0791; Found 155.0796. **m.p**. Decomposes at > 250 °C.

2-Methylpiperazine dihydrochloride (4)

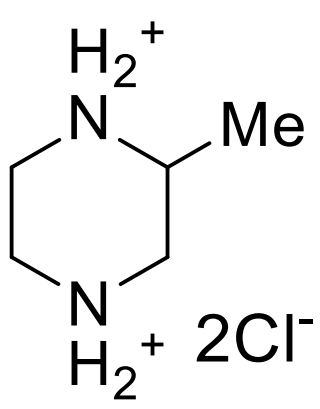

Compound **4** was prepared from 2-methylpyrazine (37.6 mg, 0.40 mmol) according to **GPA** at 60 °C. Off-white solid (52.5 mg, 96%).

^{1}H NMR (400 MHz, D_2O) δ 3.75 (dddt, *J* = 16.0, 8.9, 4.0, 2.2 Hz, 4H), 3.53 – 3.34 (m, 2H), 3.23 (dd, *J* = 14.6, 12.4 Hz, 1H), 1.44 (d, *J* = 6.5 Hz, 3H). **^{13}C NMR** (101 MHz, D_2O) δ 51.6, 48.1, 42.9, 42.5, 17.8. **FTIR** (cm^{-1}): 2973, 2901, 2792, 2705, 1418, 1079, 1057, 975, 948, 578. **HRMS** (ESI/QTOF) m/z: $[M – 2HCl + H]^+$ Calculated for $[C_5H_{13}N_2]^+$ 101.1073; Found 101.1074. **m.p**. 249-250 °C.

2,3-Dimethylpiperazine dihydrochloride (5)

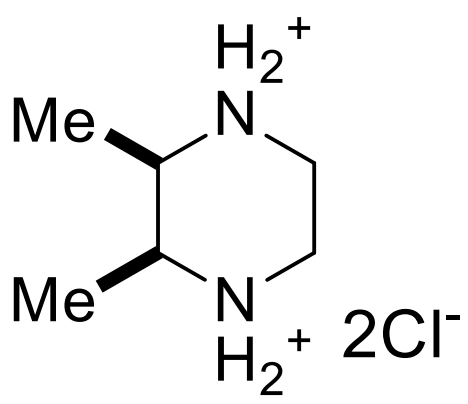

Compound **5** was prepared from 2,3-dimethylpyrazine (43.3 mg, 0.40 mmol) according to **GPA** at 60 °C. Yellow solid (59.7 mg, 99%). 10:1 dr, *cis* major. Only the major product is reported. NMR values are consistent with literature.[6]

^{1}H NMR (400 MHz, D_2O) δ 3.94 (qd, *J* = 5.7, 3.2 Hz, 2H), 3.68 – 3.56 (m, 2H), 3.55 – 3.47 (m, 2H), 1.44 (d, *J* = 6.9 Hz, 6H). **^{13}C NMR** (101 MHz, D_2O) δ 53.4, 40.0, 14.0. **FTIR** (cm^{-1}): 3387, 2924, 2657, 1587, 1444, 1355, 1216, 1139, 1111, 896. **HRMS** (ESI/QTOF) m/z: $[M – 2HCl + H]^+$ Calculated for $[C_6H_{15}N_2]^+$ 115.1230; Found 115.1228. **m.p**. 101-103°C.

2,5-dimethylpiperazine dihydrochloride (6)

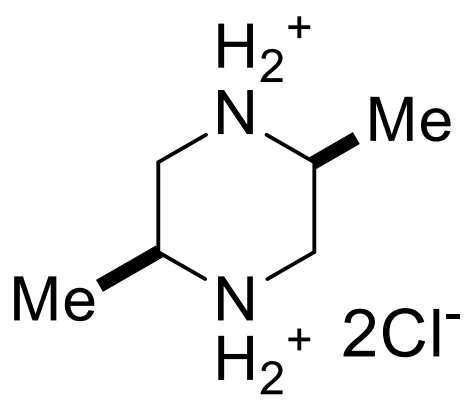

Compound **6** was prepared from 2,5-dimethylpyrazine (37.6 mg, 0.40 mmol) according to **GPA** at 60 °C. Beige solid (57.2 mg, 95%). 4.5:1 dr, *cis* major. NMR values are consistent with literature.[6]

^{1}H NMR (400 MHz, D_2O) **Major** δ 3.74 (dd, *J* = 13.8, 3.4 Hz, 2H), 3.71 – 3.63 (m, 2H), 3.23 (dd, *J* = 13.6, 12.2 Hz, 2H), 1.43 (dd, *J* = 6.4, 0.9 Hz, 6H). **Minor** δ 3.95 – 3.86 (m, 2H), 3.57 (dd, *J* = 14.4, 4.0 Hz, 2H), 3.48 (dd, *J* = 14.4, 6.8 Hz, 2H), 1.50 (dd, *J* = 7.0, 0.9 Hz, 6H). **^{13}C NMR** (101 MHz, D_2O) **Major** δ 51.2, 48.2, 17.6. **Minor** δ 49.7, 45.2, 16.5. **FTIR** (cm^{-1}): 2973, 2901, 2714, 2657, 2571, 2428, 2346, 1580, 1479, 1441, 1379, 1252, 1137, 1065, 891. **HRMS** (ESI/QTOF) m/z: $[M – 2HCl + H]^+$ Calculated for $[C_6H_{15}N_2]^+$ 115.1230; Found 115.1231. **m.p**. >250 °C.

2,6-Dimethylpiperazine dihydrochloride (7)

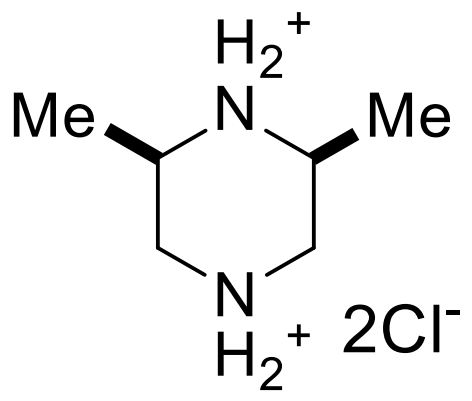

Compound **7** was prepared from 2,6-dimethylpyrazine (37.6 mg, 0.40 mmol) according to **GPA** at 60 °C. Brown solid (59.7 mg, 99%). >20:1 dr, *cis* major. NMR values are consistent with literature.**[6]**

^{1}H NMR (400 MHz, D_2O) δ 3.81 – 3.66 (m, 4H), 3.14 (dd, *J* = 14.6, 12.8 Hz, 1H), 1.42 (d, *J* = 6.4 Hz, 6H). **^{13}C NMR** (101 MHz, D_2O) δ 51.8, 47.6, 17.8. **FTIR** (cm^{-1}): 3470, 3018, 2968, 8911, 2728, 1745, 1434, 1367, 1277, 1230, 1118, 1062, 974. **HRMS** (ESI/QTOF) m/z: $[M – 2HCl + H]^+$ Calculated for $[C_6H_{15}N_2]^+$ 115.1230; Found 115.1228 **m.p**. Decomposes at > 250 °C.

2,3,5,6-Tetramethylpiperazine dihydrochloride (8)

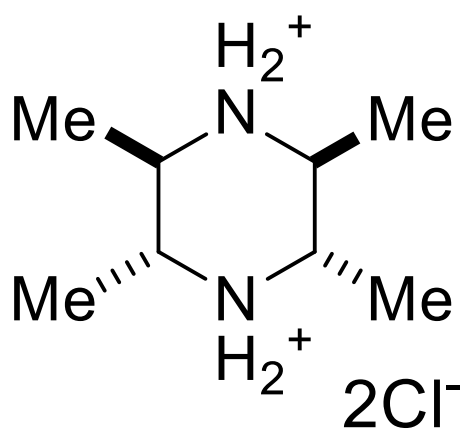

Compound **8** was prepared from 2,3,5,6-tetramethylpyrazine (54.5 mg, 0.40 mmol) according to **GPA** at 60 °C. Yellow solid (66.5 mg, 93%). 3.3:1 dr. Only the major product is reported.

^{1}H NMR (400 MHz, D_2O) δ 3.92 (qd, *J* = 5.7, 3.0 Hz, 4H), 1.47 (d, *J* = 6.7 Hz, 12H). **^{13}C NMR** (101 MHz, D_2O) δ 53.2, 15.7. **FTIR** (cm^{-1}): 3372, 2941, 2644, 2499, 1583, 1452, 1407, 1322, 1067, 1013, 689. **HRMS** (nanochip-ESI/LTQ-Orbitrap) m/z: $[M – 2HCl + H]^+$ Calculated for $[C_8H_{19}N_2]^+$ 143.1543; Found 143.1547. **m.p**. Decomposes at > 250 °C

Pyrimidines hydrogenation

Methyl 3,4,5,6-tetrahydropyrimidine-4-carboxylate (9)

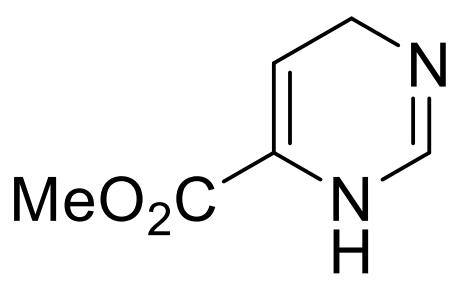

Compound **9** was prepared from methyl pyrimidine-4-carboxylate (55.3 mg, 0.40 mmol) according to **GPB** at 25 °C. Yellow solid (39.8 mg, 70%).

^{1}H NMR (400 MHz, $CDCl_3$)) δ 7.14 (q, *J* = 1.2 Hz, 1H), 5.68 (td, *J* = 3.8, 1.1 Hz, 1H), 4.21 (dd, *J* = 3.8, 1.2 Hz, 2H), 3.81 (s, 3H). **^{13}C NMR** (101 MHz, $CDCl_3$)) δ 162.9, 146.1, 129.0, 109.0, 52.6, 44.5. **FTIR** (cm^{-1}): 2987, 2972, 2901, 2361, 1458, 1449, 1406, 1383, 1250, 1075, 892. **HRMS** ((ESI/QTOF) m/z: [M + H]$^+$ Calculated for $[C_6H_9N_2O_2]^+$ 141.0659; Found 141.0662. **m.p**. 78-80 °C.

2-Methyl-3,6-dihydropyrimidine trifluoroacetate (10)

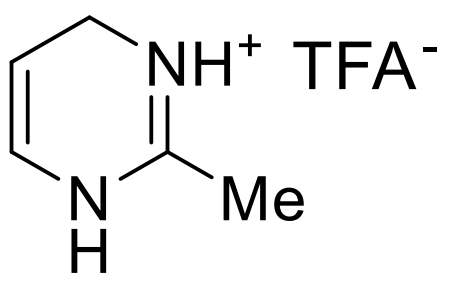

Compound **10** was prepared from 2-methylpyrimidine (37.6 mg, 0.40 mmol) according to **GPC** at 25 °C. The lyophilization step was performed without added HCl (decomposition observed otherwise) to give the TFA salt. Orange oil (77.3 mg, 92%).

^{1}H NMR (400 MHz, $CDCl_3$) δ 11.39 (br s, 1H), 10.61 (br s, 1H), 6.07 (ddt, *J* = 8.0, 4.0, 1.8 Hz, 1H), 5.02 (ddq, *J* = 8.0, 3.0, 1.5 Hz, 1H), 4.20 (dt, *J* = 3.2, 1.6 Hz, 2H), 2.25 (s, 3H). **^{13}C NMR** (101 MHz, $CDCl_3$) δ 162.0 (q, *J* = 35.1 Hz), 161.1, 122.6, 115.3 (q, *J* = 292.9 Hz), 103.8, 40.5, 17.6. **^{19}F NMR** (376 MHz, CDCl3) δ -75.79. **FTIR** (cm^{-1}): 3208, 3021, 2801, 1655, 1500, 1427, 1329, 1176, 1047, 914, 832. **HRMS** (ESI/QTOF) m/z: [M – CF_3CO_2H + H]$^+$ Calculated for $[C_5H_9N_2]^+$ 97.0760; Found 97.0758. **m.p**. Oil.

5-Bromo-2-phenyl-1,4-dihydropyrimidine hydrochloride (11)

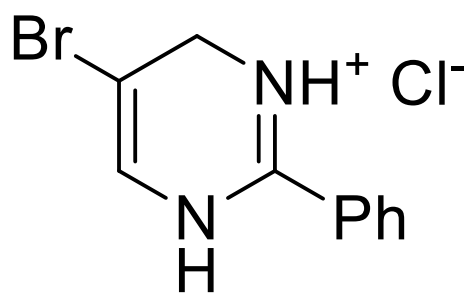

Compound **11** was prepared from 5-bromo-2-phenylpyrimidine (94.0 mg, 0.40 mmol) according to **GPA** at 25 °C for 1 h. Beige solid (108.3 mg, 99%).

^{1}H NMR (400 MHz, D_2O) δ 7.83 – 7.76 (m, 1H), 7.75 – 7.70 (m, 2H), 7.67 – 7.61 (m, 2H), 6.75 (t, *J* = 1.6 Hz, 1H), 4.57 (d, *J* = 1.7 Hz, 2H). **^{13}C NMR** (101 MHz, D_2O) δ 161.1, 137.3, 132.2, 130.3, 128.9, 125.0, 103.5, 49.2. **FTIR** (cm^{-1}): 2854, 2741, 1562, 1434, 1356, 1319, 1271, 777. **HRMS** ESI/QTOF) m/z: [M – HCl + H]$^+$ Calculated for $[C_{10}H_{10}BrN_2]^+$ 237.0022; Found 237.0019. **m.p**. 206-208 °C.

2-Phenyl-1,4,5,6-tetrahydropyrimidine hydrobromide (12)

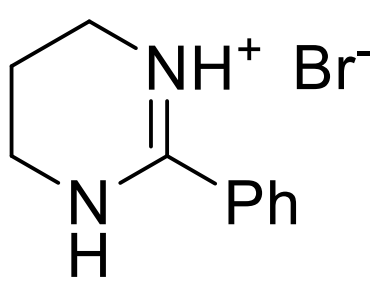

Compound **12** was prepared from 5-bromo-2-phenylpyrimidine (91.3 mg, 0.40 mmol) according to **GPA** at 60 °C. Orange solid (96.1 mg, 99%).

^{1}H NMR (400 MHz, D_2O) δ 7.76 – 7.66 (m, 2H), 7.65 – 7.57 (m, 1H), 3.62 – 3.55 (m, 2H), 2.15 – 2.05 (m, 1H). **^{13}C NMR** (101 MHz, D_2O) δ 163.4, 136.1, 132.0, 131.5, 129.7, 41.7, 20.4. **FTIR** (cm^{-1}): 3427, 3095, 2979, 1639, 1609, 1473, 1396, 1274, 1175, 1073, 970. **HRMS** (ESI/QTOF) m/z: [M - Br]$^+$ Calculated for $[C_{10}H_{13}N_2]^+$ 161.1073; Found 161.1077. **m.p**. 191-193 °C.

Ethyl 2-phenyl-1,4-dihydropyrimidine-5-carboxylate hydrochloride (13)

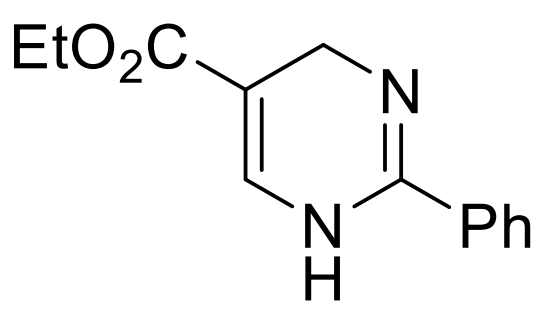

Compound **13** was prepared from ethyl 2-phenylpyrimidine-5-carboxylate (91.3 mg, 0.40 mmol) according to **GPD** at 25 °C. Purified by column chromatography (60% EtOAc in pentane, **R*f*** = 0.3) to give a yellow solid (82.2 mg, 77%). Matches literature.[7]

Pyridazines hydrogenation

3-(Trifluoromethyl)hexahydropyridazine dihydrochloride (14)

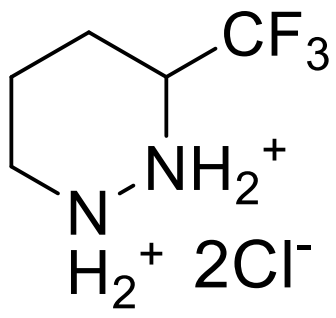

Compound **14** was prepared from 3-(trifluoromethyl)pyridazine (59.2 mg, 0.40 mmol) according to **GPA** at 60 °C. Off-white solid (73.6 mg, 81%).

^{1}H NMR (400 MHz, D_2O) δ 3.85 (ddtd, *J* = 13.5, 9.4, 6.7, 2.8 Hz, 1H), 3.46 (ddd, *J* = 12.9, 5.3, 2.6 Hz, 1H), 3.13 (td, *J* = 12.7, 3.3 Hz, 1H), 2.10 (tq, *J* = 17.5, 3.8 Hz, 2H), 1.97 – 1.81 (m, 1H), 1.82 – 1.66 (m, 1H). **^{13}C NMR** (101 MHz, D_2O) δ 126.6 (q, *J* = 278.5 Hz), 58.3 (q, *J* = 30.4 Hz), 47.4, 23.2 (q, *J* = 2.0 Hz), 21.5. **^{19}F NMR** (376 MHz, D_2O) δ -75.74. **FTIR** (cm^{-1}): 3160, 2972, 2713, 2676, 1320, 1266, 1208, 1161, 1112, 971, 698. **HRMS** (Nanochip-ESI/LTQ-Orbitrap) m/z: $[M – 2HCl + H]^+$ Calculated for $[C_5H_{10}F_3N_2]^+$ 155.0791; Found 155.0795. **m.p**. Decomposes at > 180 °C.

4-(Trifluoromethyl)hexahydropyridazine dihydrochloride (15)

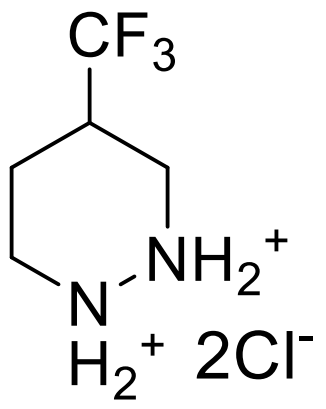

Compound **15** was prepared from 4-(trifluoromethyl)pyridazine (59.2 mg, 0.40 mmol) according to **GPA** at 60 °C. Yellow solid (84.5 mg, 83%).

^{1}H NMR (400 MHz, D_2O) 3.57 (dt, *J* = 13.3, 3.9 Hz, 1H), 3.48 (ddd, *J* = 13.8, 4.3, 1.9 Hz, 1H), 3.26 – 3.08 (m, 2H), 2.78 (dddq, *J* = 15.9, 12.0, 8.1, 4.1 Hz, 1H), 2.31 – 2.21 (m, 1H), 1.95 (dtd, *J* = 14.4, 12.3, 4.5 Hz, 1H). **^{13}C NMR** (101 MHz, D_2O) δ 129.9 (q, *J* = 278.1 Hz), 46.4, 45.7 (q, *J* = 3.4 Hz), 39.2 (q, *J* = 27.9 Hz), 22.6 (q, *J* = 2.7 Hz). **^{19}F NMR** (376 MHz, D_2O) δ -72.71. **FTIR** (cm^{-1}): 3191, 2940, 2681, 2505, 1586, 1451, 1336, 1257, 1162, 1105, 1042, 963, 889, 825. **HRMS** (Nanochip-ESI/LTQ-Orbitrap) m/z: $[M – 2HCl + H]^+$ Calculated for $[C_5H_{10}F_3N_2]^+$ 155.0791; Found 155.0795. **m.p.** 77-79 °C.

3-Phenylhexahydropyridazine dihydrochloride (16)

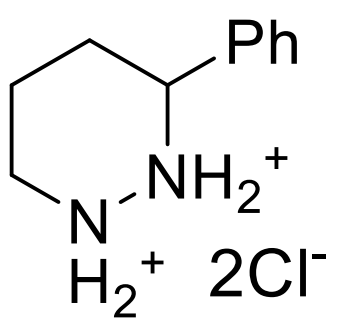

Compound **16** was prepared from 3-phenylpyridazine (62.5 mg, 0.40 mmol) according to **GPA** at 80 °C. Off-white solid (90.3 mg, 96%, 86% pure). The crude could be triturated in boiling THF to obtain analytically pure **XX** as white powder (37.6 mg, 40 %).

^{1}H NMR (400 MHz, D_2O) δ 7.51 – 7.43 (m, 5H), 4.29 – 4.21 (m, 1H), 3.53 – 3.43 (m, 1H), 3.27 – 3.10 (m, 1H), 2.24 – 1.87 (m, 4H). **^{13}C NMR** (101 MHz, D_2O) δ 140.4, 131.8, 131.8, 130.0, 62.9, 47.5, 31.0, 24.1. **FTIR** (cm^{-1}): 3201, 2957, 2785, 1493, 1428, 1406, 884, 824, 757. **HRMS** (APCI/QTOF) m/z: $[M – 2HCl + H]^+$ Calculated for $[C_9H_{13}N_2]^+$ 149.1073; Found 149.1076. **m.p.** 187-189 °C.

3,6-Dimethylhexahydropyridazine dihydrochloride (17)

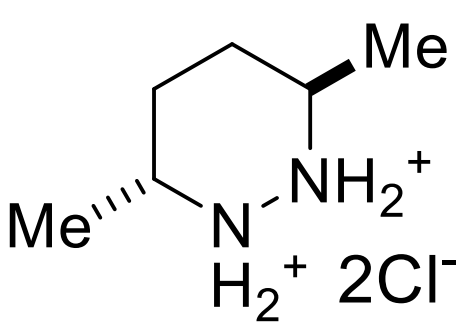

Compound **17** was prepared from 3,6-dimethylpyridazine (43.3 mg, 0.40 mmol) according to **GPA** at 25 °C. Beige solid (68.1 mg, 91%). 16:1 dr, *trans* major. Only the major product is reported.

^{1}H NMR (400 MHz, D_2O) δ 3.17 (dqd, *J* = 12.6, 6.4, 2.4 Hz, 2H), 1.95 (ddd, *J* = 12.2, 7.1, 3.6 Hz, 1H), 1.54 – 1.40 (m, 2H), 1.19 (d, *J* = 6.5 Hz, 6H). **^{13}C NMR** (101 MHz, D_2O) δ 54.8, 32.0, 20.0. **FTIR** (cm^{-1}): 2974, 2930, 2417, 2366, 2198, 2150, 2042, 1988, 1466, 1450, 1250, 1137, 1043, 1001, 601. **HRMS** (Nanochip-ESI/LTQ-Orbitrap) m/z: $[M – 2HCl + H]^+$ Calculated for $[C_6H_{15}N_2]^+$ 115.1230; Found 115.1232. **m.p.** 166-168 °C.

Methyl 6-methylhexahydropyridazine-3-carboxylate dihydrochloride (18)

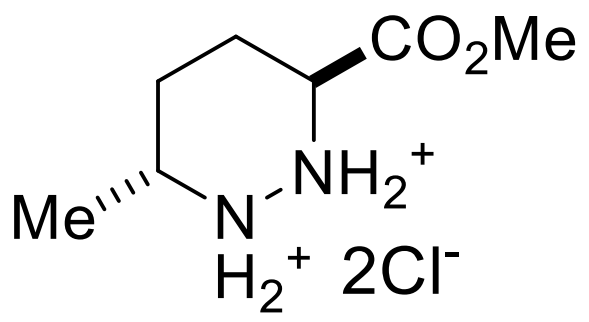

Compound **18** was prepared from methyl 6-methylpyridazine-3-carboxylate (60.9 mg, 0.40 mmol) according to **GPA** at 25 °C. Yellow hygroscopic solid (83.2 mg, 90%). 10:1 dr, *trans* major. Only the major product is reported.

^{1}H NMR (400 MHz, D_2O) 3.94 (dd, J = 11.6, 3.1 Hz, 10H), 3.80 (s, 18H), 3.33 (dddd, J = 14.7, 9.8, 6.6, 3.2 Hz, 12H), 2.29 – 2.21 (m, 12H), 2.14 (dq, J = 14.2, 3.1 Hz, 10H), 1.81 – 1.60 (m, 22H), 1.30 (d, J = 6.6 Hz, 28H). **^{13}C NMR** (101 MHz, D_2O) δ 174.1, 58.8, 55.8, 55.8, 30.7, 27.9, 19.1. **FTIR** (cm^{-1}): 2953, 2851, 2701, 2536, 1737, 1436, 1309, 1151, 1030, 883. **HRMS** (Nanochip-ESI/LTQ-Orbitrap) m/z: [M – 2HCl + H]$^+$ Calculated for $[C_7H_{15}N_2O_2]^+$ 159.1128; Found 159.1132. **m.p.** Hygroscopic solid.

Methyl 5-methylhexahydropyridazine-3-carboxylate dihydrochloride (19)

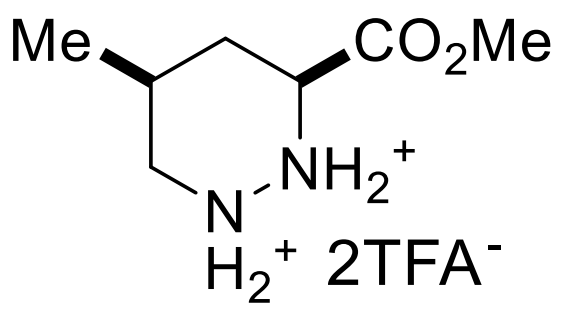

Compound **19** was prepared from methyl 5-methylpyridazine-3-carboxylate (60.9 mg, 0.40 mmol) according to **GPA** with 200mg of 4Å molecular sieves at 60 °C. Trituration with $CDCl_3$ afforded off-white solid (111 mg, 72%). >20:1 dr, *cis* major.

^{1}H NMR (400 MHz, D_2O) δ 4.00 (dd, J = 12.1, 3.1 Hz, 1H), 3.80 (s, 3H), 3.41 (ddd, J = 12.9, 4.3, 1.6 Hz, 1H), 2.70 (t, J = 12.5 Hz, 1H), 2.28 (d, J = 13.2 Hz, 1H), 2.13 – 2.01 (m, 1H), 1.38 (dt, J = 13.6, 12.0 Hz, 1H), 1.03 (d, J = 6.7 Hz, 3H). **^{13}C NMR** (101 MHz, D_2O) ^{13}C NMR (101 MHz, D_2O) δ 175.1*, 56.7, 53.1, 50.3, 33.0, 27.0, 17.5. *Ester carbon is not resolved but is visible by HMBC. TFA carbons are not resolved. **FTIR** (cm^{-1}): 2957, 2673, 2558, 1736, 1438, 1269, 1217, 1180, 1044, 1019. **HRMS** (Nanochip-ESI/LTQ-Orbitrap) m/z: [M – 2TFA + H]$^+$ Calculated for $[C_7H_{15}N_2O_2]^+$ 159.1128; Found 159.1133. **m.p.** >250 °C.

6-Phenyltetrahydropyridazin-3(2*H*)-one (20)

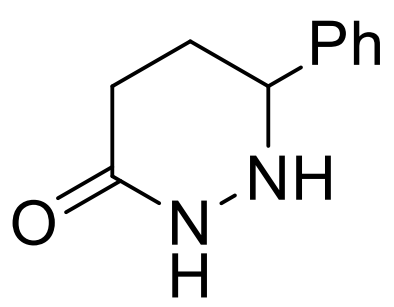

Compound **20** was prepared from 6-phenylpyridazin-3-ol (68.9 mg, 0.40 mmol) according to **GPD** at 25 °C with 200mg of 4Å molecular sieves in THF (0.4 mL). Purified by column chromatography (7% MeOH in DCM, **R*f*** = 0.25) to give a yellow solid (50.4 mg, 72%). Matches literature.[8]

Methyl 6-oxohexahydropyridazine-3-carboxylate (21)

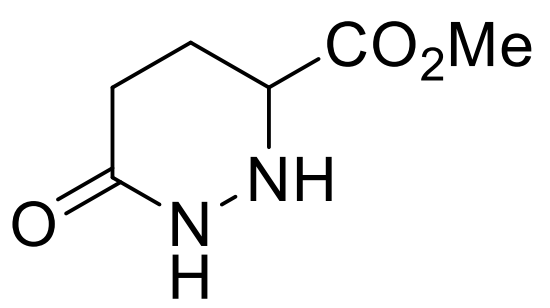

Compound **21** was prepared from methyl 6-hydroxypyridazine-3-carboxylate (61.7 mg, 0.40 mmol) according to **GPD** at 25 °C with 200mg of 4Å molecular sieves in THF (0.4 mL). Purified by column chromatography (7% MeOH in DCM, **R*f*** = 0.25) to give a yellow solid (31.0 mg, 49%). Matches literature.[8]

[6,6]-fused heteroarene hydrogenation

3-Amino-1,2,3,4-tetrahydroquinoline (22)

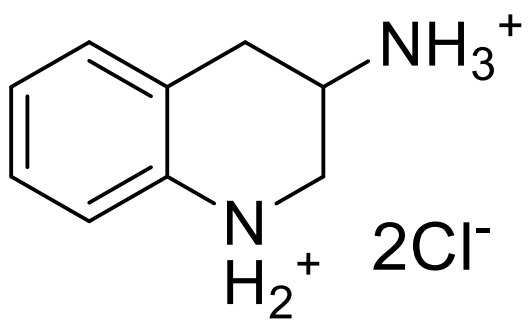

Compound **22** was prepared from 3-aminoquinoline (57.7 mg, 0.40 mmol) according to **GPA** at 25 °C. White solid (80.5 mg, 91%).

^{1}H NMR (400 MHz, D_2O) δ 7.32 – 7.22 (m, 2H), 7.12 (td, *J* = 7.5, 1.2 Hz, 1H), 7.05 – 6.96 (m, 1H), 3.99 (tdd, *J* = 7.3, 5.3, 3.1 Hz, 1H), 3.71 (ddd, *J* = 12.4, 3.2, 1.5 Hz, 1H), 3.47 (ddd, *J* = 12.4, 7.7, 1.2 Hz, 1H), 3.35 (dd, *J* = 17.0, 5.3 Hz, 1H), 3.01 (dd, *J* = 16.9, 7.1 Hz, 1H). **^{13}C NMR** (101 MHz, D_2O) δ 139.3, 131.0, 126.5, 124.1, 121.2, 46.3, 45.6, 32.3. **FTIR** (cm^{-1}): 2980, 2924, 2749, 1595, 1542, 1531, 773. **HRMS** (APCI/QTOF) m/z: [M -2 HCl + H]$^+$ Calculated for $[C_9H_{13}N_2]^+$ 149.1073; Found 149.1076. **m.p.** Decomposes at > 260 °C.

3-Methoxy-1,2,3,4-tetrahydroquinoline (23)

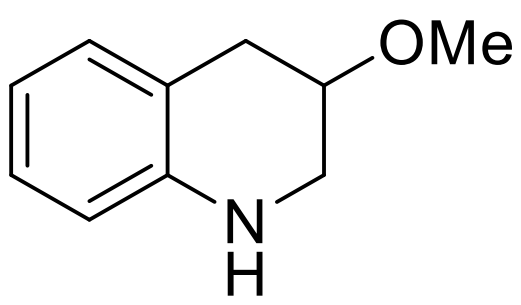

Compound **23** was prepared from 3-methoxyquinoline (63.7 mg, 0.40 mmol) according to **GPD** at 25 °C. Purified by column chromatography (20% EtOAc in pentane, **R*f*** = 0.25) to give a yellow oil (55.5 mg, 85%). Matches literature.[9]

3-Bromo-1,2,3,4-tetrahydroquinoline (24)

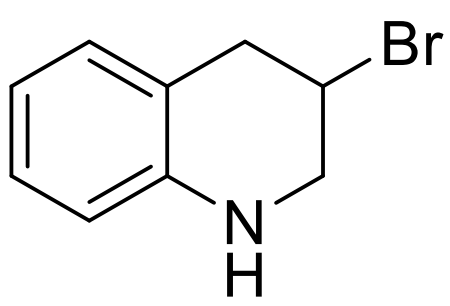

Compound **24** was prepared from 3-methoxyquinoline (83.2 mg, 0.40 mmol) according to **GPD** at 25 °C. Filtered over a silica pad (20% EtOAc in pentane) to give a yellow oil (79.8 mg, 94%). Matches literature.[10]

6-Iodo-1,2,3,4-tetrahydroquinoline (26)

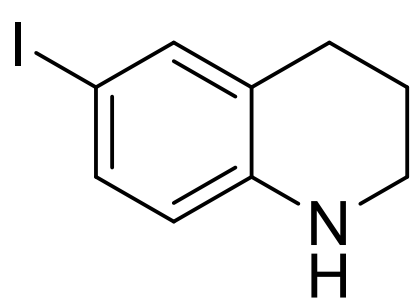

Compound **25** was prepared from 6-iodoquinoline (63.7 mg, 0.40 mmol) according to **GPD** at 25 °C for 1 h. Purified by column chromatography (20% EtOAc in pentane, **R*f*** = 0.25) to give a brown solid (95.3 mg, 92%). *Decomposes within days to a purple solid*.

^{1}H NMR (400 MHz, $CDCl_3$) δ 7.23 – 7.21 (m, 1H), 7.19 (dd, *J* = 8.4, 2.1 Hz, 1H), 6.24 (d, *J* = 8.3 Hz, 1H), 3.84 (br s, 1H), 3.31 – 3.25 (m, 2H), 2.71 (t, *J* = 6.4 Hz, 2H), 1.95 – 1.85 (m, 2H). **^{13}C NMR** (101 MHz, $CDCl_3$) δ 144.5, 137.8, 135.4, 124.2, 116.2, 41.8, 27.1, 21.7. **HRMS** (Nanochip-ESI/LTQ-Orbitrap) m/z: [M + H]$^+$ Calculated for $[C_9H_{11}IN]^+$ 259.9931; Found 259.9939.

6-Methoxy-1,2,3,4-tetrahydroquinoline (26)

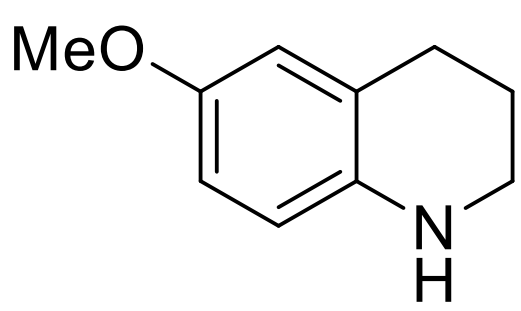

Compound **25** was prepared from 6-methoxyquinoline (63.7 mg, 0.40 mmol) according to **GPB** at 25 °C. Yellow oil (53.5 mg, 82%). Matches literature.[11]

5-nitro-1,2,3,4-tetrahydroisoquinoline (27)

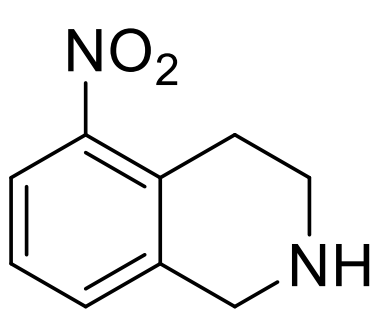

Compound **27** was prepared from 5-nitroisoquinoline (69.6 mg, 0.40 mmol) according to **GPB** at 25 °C. Brown oil (60.0mg, 84%) Matches literature.[12]

6-Bromo-1,2,3,4-tetrahydroisoquinoline hydrochloride (28)

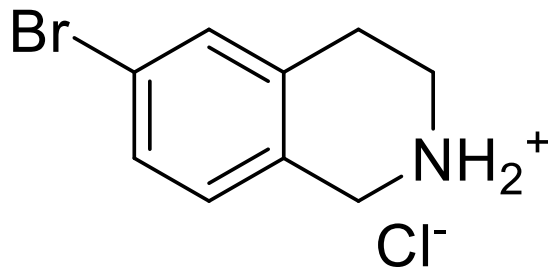

Compound **28** was prepared from 6-bromoisoquinoline (83.2 mg, 0.40 mmol) according to **GPA** at 25 °C. Off-white solid (58.5 mg, 86%).

^{1}H NMR (400 MHz, D_2O) δ 7.53 – 7.45 (m, 1H), 7.15 (d, *J* = 8.2 Hz, 0H), 4.35 (s, 1H), 3.53 (t, *J* = 6.4 Hz, 1H), 3.13 (t, *J* = 6.4 Hz, 1H). **^{13}C NMR** (101 MHz, D_2O) δ 136.4, 134.3, 132.7, 131.2, 129.6, 123.8, 46.9, 44.0, 27.0. **FTIR** (cm^{-1}): 2917, 2780, 2725, 1591, 1454, 1380, 1179, 1004, 851. **HRMS** (ESI/QTOF) m/z: [M - Cl]$^+$ Calculated for $[C_9H_{11}BrN]^+$ 212.0069; Found 212.0075. **m.p**. Decomposes at > 275 °C.

8-Chloro-1,2,3,4-tetrahydroisoquinoline hydrochloride (29)

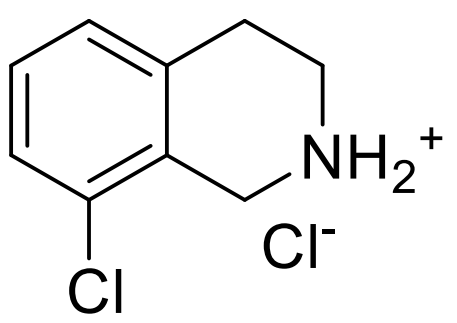

Compound **29** was prepared from 8-chloroisoquinoline (65.4 mg, 0.40 mmol) according to **GPA** at 60 °C. White solid (72.7 mg, 89%).

^{1}H NMR (400 MHz, D_2O) δ 7.41 (dd, *J* = 8.0, 1.3 Hz, 1H), 7.34 (t, *J* = 7.8 Hz, 1H), 7.26 (dd, *J* = 7.6, 1.3 Hz, 1H), 4.43 (s, 2H), 3.53 (t, *J* = 6.3 Hz, 2H), 3.16 (t, *J* = 6.4 Hz, 2H). **^{13}C NMR** (101 MHz, D_2O) δ 36.6, 134.5, 130.4, 130.3, 128.3, 45.4, 43.8, 27.4. **FTIR** (cm^{-1}): 2898, 2753, 2585, 1585, 1465, 1431, 1351, 1146, 1009, 980. **HRMS** (ESI/QTOF) m/z: [M - Cl]$^+$ Calculated for $[C_9H_{11}ClN]^+$ 168.0575; Found 168.0581. **m.p**. 246-248 °C.

1,2,3,4-tetrahydro-1,8-naphthyridine (30)

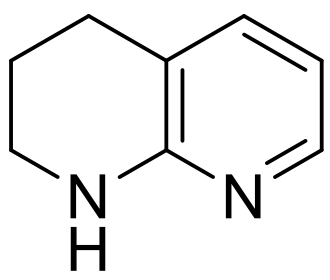

Compound **30** was prepared from 1,8-naphthyridine (51.0 mg, 0.40 mmol) according to **GPB** at 25 °C. Yellow oil (72.7 mg, 95%). Matches literature. [13]

7-Methyl-1,2,3,4-tetrahydro-1,8-naphthyridine (31a) &
2-Methyl-1,2,3,4-tetrahydro-1,8-naphthyridine (31b)

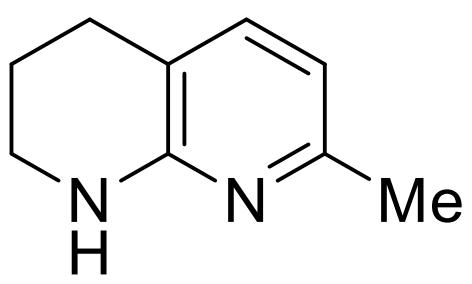

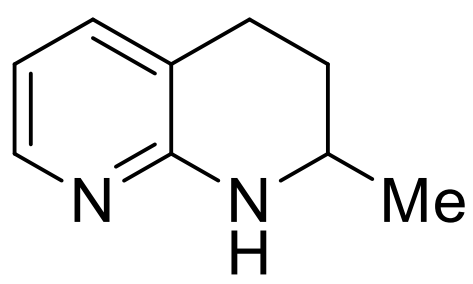

Compound **31a** and **31b** were prepared from 2-methyl-1,8-naphthyridine (57.7 mg, 0.40 mmol) according to **GPB** at 25 °C. Purified by column chromatography (100% EtOAc) to give **31a** (**R*f*** = 0.3, 18.0 mg, 30%) and **31b** (**R*f*** 0.2, 18.3 mg, 31%). Matches literature.[14]

Methyl 5,6,7,8-tetrahydro-1,8-naphthyridine-2-carboxylate (32)

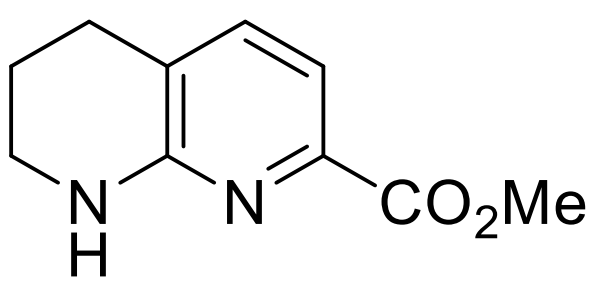

Compound **32** was prepared from methyl 1,8-naphthyridine-2-carboxylate (75.3 mg, 0.40 mmol) according to **GPD** at 25 °C. Purified by column chromatography (100% EtOAc in pentane, **R*f*** = 0.4) to give a yellow oil (65.3 mg, 85%).

^{1}H NMR (400 MHz, $CDCl_3$) δ 7.34 (d, *J* = 7.4 Hz, 1H), 7.25 – 7.22 (m, 1H), 5.13 (s, 1H), 3.93 (s, 3H), 3.44 (td, *J* = 5.7, 2.6 Hz, 2H), 2.78 (t, *J* = 6.3 Hz, 2H), 1.92 (p, *J* = 6.2 Hz, 2H). **^{13}C NMR** (101 MHz, $CDCl_3$) δ 166.5, 156.1, 144.3, 136.5, 121.2, 114.9, 52.7, 41.6, 27.2, 21.0. **FTIR** (cm^{-1}): 3258, 2947, 1716, 1599, 1526, 1431, 1342, 1297, 1201, 1130, 1074, 1010. **HRMS** (Nanochip-ESI/LTQ-Orbitrap) m/z: [M + H]$^+$ Calculated for $[C_{10}H_{13}N_2O_2]^+$ 193.0972; Found 193.0977. **m.p**. Oil.

Ethyl 2-(trifluoromethyl)-5,6,7,8-tetrahydro-1,8-naphthyridine-3-carboxylate (33)

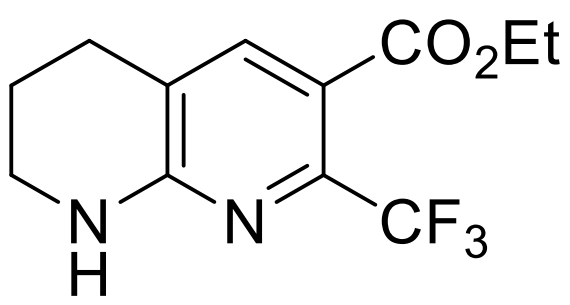

Compound **33** was prepared from ethyl 2-(trifluoromethyl)-1,8-naphthyridine-3-carboxylate (108 mg, 0.40 mmol) according to **GPD** at 25 °C for 1 h. Purified by column chromatography (10% MeOH in DCM, **R*f*** = 0.8) to give a beige solid (85.1 mg, 85%).

^{1}**H NMR** (400 MHz, $CDCl_3$) δ 7.64 (s, 1H), 5.68 (s, 1H), 4.32 (q, *J* = 7.1 Hz, 2H), 3.47 (td, *J* = 5.8, 2.7 Hz, 2H), 2.78 (t, *J* = 6.3 Hz, 2H), 1.93 (p, *J* = 6.1 Hz, 2H), 1.35 (t, *J* = 7.2 Hz, 3H). ^{13}**C NMR** (101 MHz, $CDCl_3$) δ 165.9, 156.6, 144.2 (q, *J* = 34.7 Hz), 138.5, 118 (m), 118.1, 115.3, 61.6, 41.6, 26.4, 20.4, 14.1. CF_3 carbon (~118 ppm) not resolved to see the quadruplet. ^{19}**F NMR** (376 MHz, $CDCl_3$) δ -64.28. **FTIR** (cm^{-1}): 3354, 2987, 2901, 1708, 1604, 1560, 1408, 1393, 1330, 1274, 1232, 1137, 1075, 1057, 790, 639. **HRMS** (ESI/QTOF) m/z: $[M + H]^+$ Calculated for $[C_{12}H_{14}F_3N_2O_2]^+$ 275.1002; Found 275.1009. **m.p**. 102-104 °C.

1,2,3,4-Tetrahydro-1,5-naphthyridine (34)

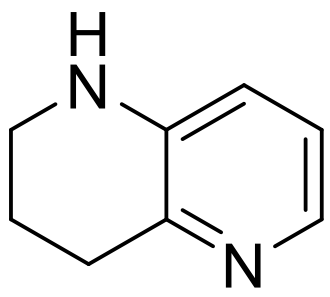

Compound **34** was prepared from 1,5-naphthyridine (52.1 mg, 0.40 mmol) according to **GPB** at 25 °C. Beige solid (44.5 mg, 83%). Matches literature.[11]

7-Bromo-1,2,3,4-tetrahydro-1,5-naphthyridine (35)

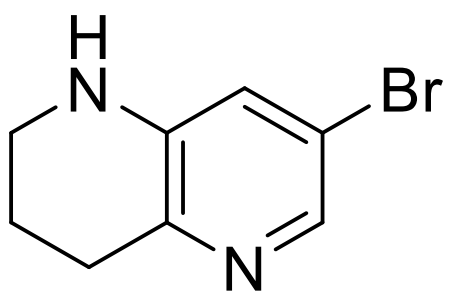

Compound **35** was prepared from 3-bromo-1,5-naphthyridine (83.6 mg, 0.40 mmol) according to **GPD** at 25 °C. Purified by column chromatography (50% EtOAc in pentane **R*f*** = 0.5) to give a beige solid (39.4 mg, 46%).

^{1}**H NMR** (400 MHz, $CDCl_3$) δ 7.87 (d, *J* = 2.0 Hz, 1H), 6.86 (d, *J* = 1.9 Hz, 1H), 3.88 (s, 1H), 3.29 (t, *J* = 5.5 Hz, 2H), 2.87 (t, *J* = 6.5 Hz, 2H), 2.07 – 1.95 (m, 2H). ^{13}**C NMR** (101 MHz, $CDCl_3$) δ 141.9, 141.3, 138.2, 121.9, 118.1, 41.4, 30.1, 21.5. **FTIR** (cm^{-1}): 3248, 2921, 2853, 1580, 1517, 1453, 1351, 1282, 1220, 1172, 1066, 1005. **HRMS** (Nanochip-ESI/LTQ-Orbitrap) m/z: $[M + H]^+$ Calculated for $[C_8H_{10}BrN_2]^+$ 213.0022; Found 213.0032. **m.p**. 140-142 °C.

5,6,7,8-Tetrahydro-2,7-naphthyridin-1(2*H*)-one hydrochloride (36)

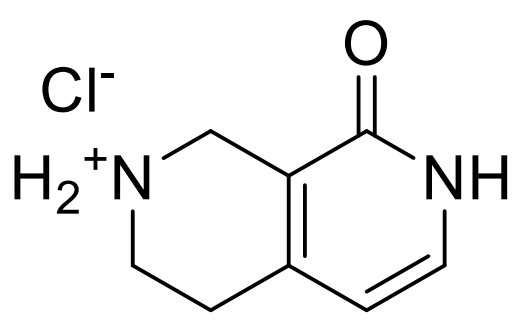

Compound **36** was prepared from 2,7-naphthyridin-1-ol (58.4 mg, 0.40 mmol) according to **GPA** at 25 °C**.** Off-white solid (70.9 mg, 95%).

^{1}**H NMR** (400 MHz, D_2O) δ 7.46 (d, *J* = 6.7 Hz, 1H), 6.47 (d, *J* = 6.7 Hz, 1H), 4.11 (s, 2H), 3.51 (t, *J* = 6.3 Hz, 2H), 3.04 (dd, *J* = 7.0, 5.5 Hz, 2H). ^{13}**C NMR** (101 MHz, D_2O) δ 164.5, 150.4, 135.7, 120.4, 112.2, 42.8, 42.8, 27.2. **FTIR** (cm^{-1}): 3016, 2958, 2916, 2752, 1641, 1584, 1567, 1538, 1417, 1322, 1293. **HRMS** (Nanochip-ESI/LTQ-Orbitrap) m/z: [M – 2HCl + H]$^+$ Calculated for $[C_8H_{11}N_2O]^+$ 151.0866; Found 151.0871. **m.p**. Decomposes at > 250 °C.

2-(trifluoromethyl)-1,2,3,4-tetrahydroquinoxaline (37)

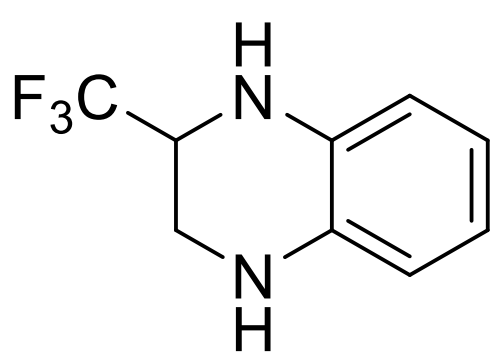

Compound **37** was prepared from 2-(trifluoromethyl)quinoxaline (79.3 mg, 0.40 mmol) according to **GPB** at 25 °C**.** Brown solid (80.1 mg, 99%).

^{1}**H NMR** (400 MHz, DMSO- d_6) δ 6.58 – 6.46 (m, 1H), 6.49 – 6.36 (m, 3H), 6.11 (d, *J* = 4.5 Hz, 1H), 5.44 (s, 1H), 4.06 (qq, *J* = 8.0, 4.0 Hz, 1H), 3.36 – 3.29 (m, 2H), 3.16 (ddt, *J* = 12.0, 3.9, 2.0 Hz, 1H). ^{13}**C NMR** (101 MHz, DMSO-d_6) δ 132.9, 131.7, 125.9*, 117.8, 117.3, 113.9, 113.5, 51.0 (q, *J* = 28.7 Hz), 37.8 (q, *J* = 2.1 Hz). *CF_3 carbon quadruplet not resolved. ^{19}**F NMR** (376 MHz, DMSO-d_6) -74.50. **FTIR** (cm^{-1}): 3356, 3313, 1599, 1509,

1385, 1324, 1265, 1155, 10776, 1032, 910. **HRMS** (Nanochip-based ESI/LTQ-Orbitrap) m/z: [M + H]+ Calculated for $[C_9H_{10}F_3N_2]^+$ 203.0791; Found 203.0795. **m.p**. 118-120 °C.

1,2,3,4-tetrahydropyrido[2,3-*b*]pyrazine (38)

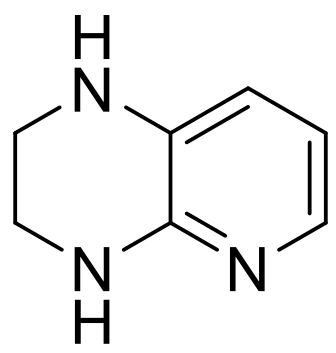

Compound **38** was prepared from pyrido[2,3-b]pyrazine (52.5 mg, 0.40 mmol) according to **GPD** at 25 °C**.** Purified by column chromatography (10% MeOH in DCM, **R*f*** = 0.25) to give a beige solid (31.4 mg, 58%). Matches literature.[15]

7-bromo-1,2,3,4-tetrahydropyrido[2,3-*b*]pyrazine (39)

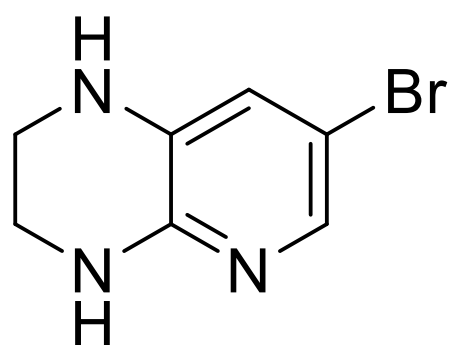

Compound **39** was prepared from 7-bromopyrido[2,3-b]pyrazine (84.0 mg, 0.40 mmol) according to **GPD** at 25 °C**.** Purified by column chromatography (50% EtOAc in pentane, **R*f*** = 0.4) to give a brown solid (29.1 mg, 34%).

^{1}H NMR (400 MHz, $CDCl_3$) δ 7.49 (d, *J* = 2.1 Hz, 1H), 6.72 (dd, *J* = 2.0, 0.8 Hz, 1H), 4.71 (br s, 1H), 3.76 (br s, 1H), 3.56 – 3.42 (m, 2H), 3.38 (t, *J* = 4.7 Hz, 2H). **^{13}C NMR** (101 MHz, $CDCl_3$) δ 145.8, 137.0, 130.2, 120.4, 107.9, 40.6, 40.1. **FTIR** (cm^{-1}): 3216, 2988, 2918, 1578, 1530, 1441, 1354, 1282, 1228, 1080. **HRMS** (Nanochip-based ESI/LTQ-Orbitrap) m/z: [M + H]+ Calculated for $[C_7H_9BrN_3]^+$ 213.9974; Found 213.9984.**m.p**. 123-125 °C.

[6,5]-fused heteroarene hydrogenation

Methyl 1-tosyl-4,5,6,7-tetrahydro-1*H*-pyrrolo[2,3-*c*]pyridine-5-carboxylate (40)

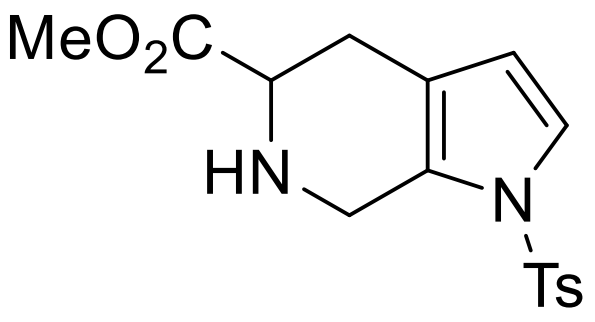

Compound **40** was prepared from methyl 1-tosyl-1*H*-pyrrolo[2,3-*c*]pyridine-5-carboxylate (132 mg, 0.40 mmol) according to **GPB** at 25 °C. Purified by column chromatography (20% EtOAc in DCM, **R*f*** = 0.2) to give a off-white solid (97.6 mg, 73%).

^{1}**H NMR** (400 MHz, $CDCl_3$) δ 7.69 – 7.62 (m, 2H), 7.30 – 7.27 (m, 2H), 7.14 (d, *J* = 3.3 Hz, 1H), 6.11 (d, *J* = 3.3 Hz, 1H), 4.19 (dt, *J* = 16.5, 1.1 Hz, 1H), 3.97 (dt, *J* = 16.7, 2.6 Hz, 1H), 3.73 (s, 3H), 3.56 (dd, *J* = 9.7, 4.6 Hz, 1H), 2.78 (ddd, *J* = 15.7, 4.7, 2.0 Hz, 1H), 2.60 (ddt, *J* = 15.9, 9.8, 2.3 Hz, 1H), 2.40 (s, 3H). ^{13}**C NMR** (173.4, 145.1, 136.2, 130.2, 127.4, 126.9, 121.1, 120.8, 112.4, 55.4, 52.3, 42.7, 27.3, 21.8. **FTIR** (cm^{-1}): 2972, 2945, 1734, 1434, 1361, 1275, 1240, 1186, 1143, 1054, 992. **HRMS** (ESI/QTOF) m/z: $[M + H]^+$ Calculated for $[C_{16}H_{19}N_2O_4S]^+$ 335.1060; Found 335.1066. **m.p**. 119-121 °C.

1-(*Tert*-butyl) 5-methyl 4,5,6,7-tetrahydro-1*H*-pyrrolo[2,3-*c*]pyridine-1,5-dicarboxylate (41)

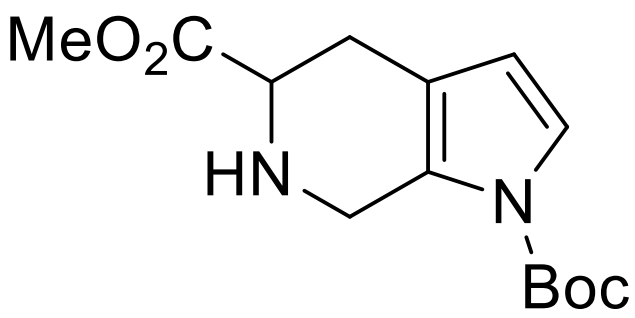

Compound **41** was prepared from 1-(*tert*-butyl) 5-methyl 1*H*-pyrrolo[2,3-*c*]pyridine-1,5-dicarboxylate (110 mg, 0.40 mmol) according to **GPB** at 25 °C**.** Purified by column chromatography (60% EtOAc in pentane, **R*f*** = 0.2) to give a yellow oil (62.8 mg, 56%).

^{1}**H NMR** (400 MHz, $CDCl_3$) δ 7.13 (d, *J* = 3.3 Hz, 1H), 6.02 (d, *J* = 3.4 Hz, 1H), 4.28 (dt, *J* = 17.1, 1.2 Hz, 1H), 4.06 (dtd, *J* = 17.0, 2.5, 0.9 Hz, 1H), 3.77 (s, 3H), 3.64 (dd, *J* = 9.7, 4.6 Hz, 1H), 2.83 (dddd, *J* = 15.7, 4.6, 2.3, 0.8 Hz, 1H), 2.66 (ddt, *J* = 15.7, 9.7, 2.3 Hz, 1H), 1.91 – 1.85 (m, 1H), 1.57 (s, 9H). ^{13}**C NMR** 173.7, 149.2, 127.6, 119.6, 119.2, 110.7, 83.7, 55.5, 52.1, 43.9, 28.1, 27.3. **FTIR** (cm^{-1}): 3475, 2977, 2928, 2727, 1738, 1434, 1341, 1280, 1230, 1140, 1061, 976. **HRMS** (ESI/QTOF) m/z: $[M + H]^+$ Calculated for $[C_{14}H_{21}N_2O_4]^+$ 281.1496; Found 281.1484.**m.p**. Oil.

1-(*Tert*-butyl) 3-methyl 2,3-dihydro-1*H*-pyrrolo[2,3-*c*]pyridine-1,3-dicarboxylate (42)

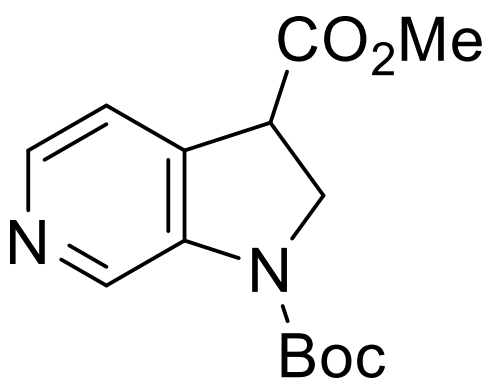

Compound **42** was prepared from 1-(*tert*-butyl) 3-methyl 1*H*-pyrrolo[2,3-*c*]pyridine-1,3-dicarboxylate (110 mg, 0.40 mmol) according to **GPB** at 25 °C**.** Purified by column chromatography (40% EtOAc in pentane, **R*f*** = 0.2) to give a yellow oil (71.2 mg, 64%). Started decomposing within days.

^{1}**H NMR** 400 MHz, $CDCl_3$, 1:1 mixture of rotamers) δ 9.12 (br s, 0.5H), 8.75 (br s, 0.5H), 8.26 (d, *J* = 4.9 Hz, 1H), 7.33 (dt, *J* = 4.9, 1.0 Hz, 1H), 4.46 – 4.33 (m, 1H), 4.25 (dd, *J* = 10.6, 6.2 Hz, 1H), 4.13 (td, *J* = 12.4, 6.4 Hz, 1H), 3.81 (s, 3H), 1.60 – 1.55 (m, 9H). ^{13}**C NMR** (101 MHz, $CDCl_3$) δ 170.7, 168.4, 143.7, 136.8, 120.3, 87.3, 53.1, 49.3, 44.9, 28.5. 2 carbons unresolved. **HRMS** (ESI/QTOF) m/z: $[M + Na]^+$ Calculated for $[C_{14}H_{18}N_2NaO_4]^+$ 301.1159; Found 301.1158. **m.p**. Oil.

Methyl 1,2,3,4-tetrahydropyrrolo[1,2-*b*]pyridazine-6-carboxylate (43)

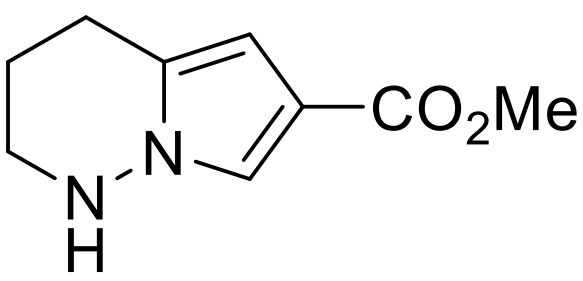

Compound **43** was prepared from methyl pyrrolo[1,2-*b*]pyridazine-6-carboxylate (70.5 mg, 0.40 mmol) according to **GPD** at 25 °C**.** Purified by column chromatography (50% EtOAc in pentane, **R*f*** = 0.3) to give a off-white solid (55.5 mg, 77%).

^{1}**H NMR** (400 MHz, $CDCl_3$) δ 7.19 (d, *J* = 2.0 Hz, 1H), 6.25 (dt, *J* = 2.1, 1.2 Hz, 1H), 4.57 (s, 1H), 3.78 (s, 3H), 3.32 – 3.25 (m, 2H), 2.88 (td, *J* = 6.9, 1.2 Hz, 2H), 1.91 – 1.82 (m, 2H). ^{13}**C NMR** (101 MHz, $CDCl_3$) δ 165.6, 125.6, 122.4, 111.9, 104.9, 51.1, 46.4, 22.2, 20.9.**FTIR** (cm^{-1}): 2947, 2924, 1700, 1518, 1459, 1383, 1261, 1199, 1137, 1000, 924, 827, 767. **HRMS** (Nanochip-based ESI/LTQ-Orbitrap) m/z: $[M + H]^+$ Calculated for $[C_9H_{13}N_2O_2]^+$ 181.0972; Found 181.0977. **m.p**. 89-91 °C.

4,5,6,7-Tetrahydropyrazolo[1,5-*a*]pyrimidine-3-carbonitrile (44)

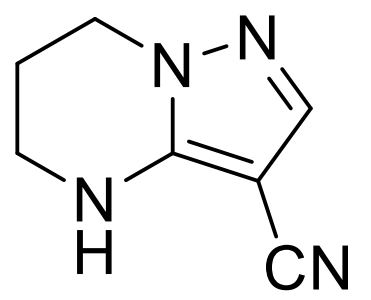

Compound **44** was prepared from pyrazolo[1,5-*a*]pyrimidine-3-carbonitrile (57.7 mg, 0.40 mmol) according to **GPD** at 25 °C**.** Purified by column chromatography (80% EtOAc in pentane, **R*f*** = 0.3) to give a yellow solid (54.5 mg, 92%).

^{1}H NMR (400 MHz, $CDCl_3$) δ 7.45 (s, 1H), 4.75 (br s, 1H), 4.09 (t, *J* = 6.1 Hz, 2H), 3.43 (ddd, *J* = 8.1, 4.5, 2.4 Hz, 2H), 2.18 (p, *J* = 6.0 Hz, 2H). **^{13}C NMR** (101 MHz, $CDCl_3$) δ 149.2, 140.5, 114.7, 72.6, 45.3, 39.3, 21.6. **FTIR** (cm^{-1}): 3220, 2952, 2205, 1594, 1574, 1470, 1364, 1268, 1199, 1090, 991, 931. **HRMS** (Nanochip-based ESI/LTQ-Orbitrap) m/z: $[M + H]^+$ Calculated for $[C_7H_9N_4]^+$ 149.0822; Found 149.0825. **m.p**. 202-204 °C.

4,5,6,7-Tetrahydropyrazolo[1,5-*a*]pyrimidine-3-carbonitrile hydrochloride (45)

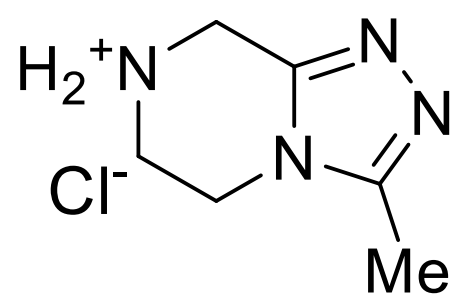

Compound **45** was prepared from pyrazolo[1,5-*a*]pyrimidine-3-carbonitrile (57.7 mg, 0.40 mmol) according to **GPC** at 60 °C with 6.(184 µL, 6.0 equiv.) of TFA**.** Hygroscopic brown solid (69.1 mg, 99%).

^{1}H NMR (400 MHz, D_2O) δ 4.80 (s, 52H), 4.53 – 4.46 (m, 4H), 3.91 – 3.86 (m, 4H), 2.68 (s, 3H). **^{13}C NMR** (101 MHz, D_2O) δ 155.5, 148.0, 42.7, 42.5, 42.0, 11.3. **FTIR** (cm^{-1}): 3360, 2625, 1601, 1571, 1422, 1338, 1109, 1042, 999, 890, 763. **HRMS** (ESI/QTOF) m/z: $[M – Cl]^+$ Calculated for[$C_6H_{11}N_4]^+$ 139.0978; Found 139.0978. **m.p**. Hygroscopic solid.

Alternatively, the crude mixture could be stirred with excess solid K_2CO_3 and purified by column chromatography (100% MeOH) to give pure product as a free base (14 mg, 25 %). Free base matches literature.[16]

NMR Spectra

Substates synthesis

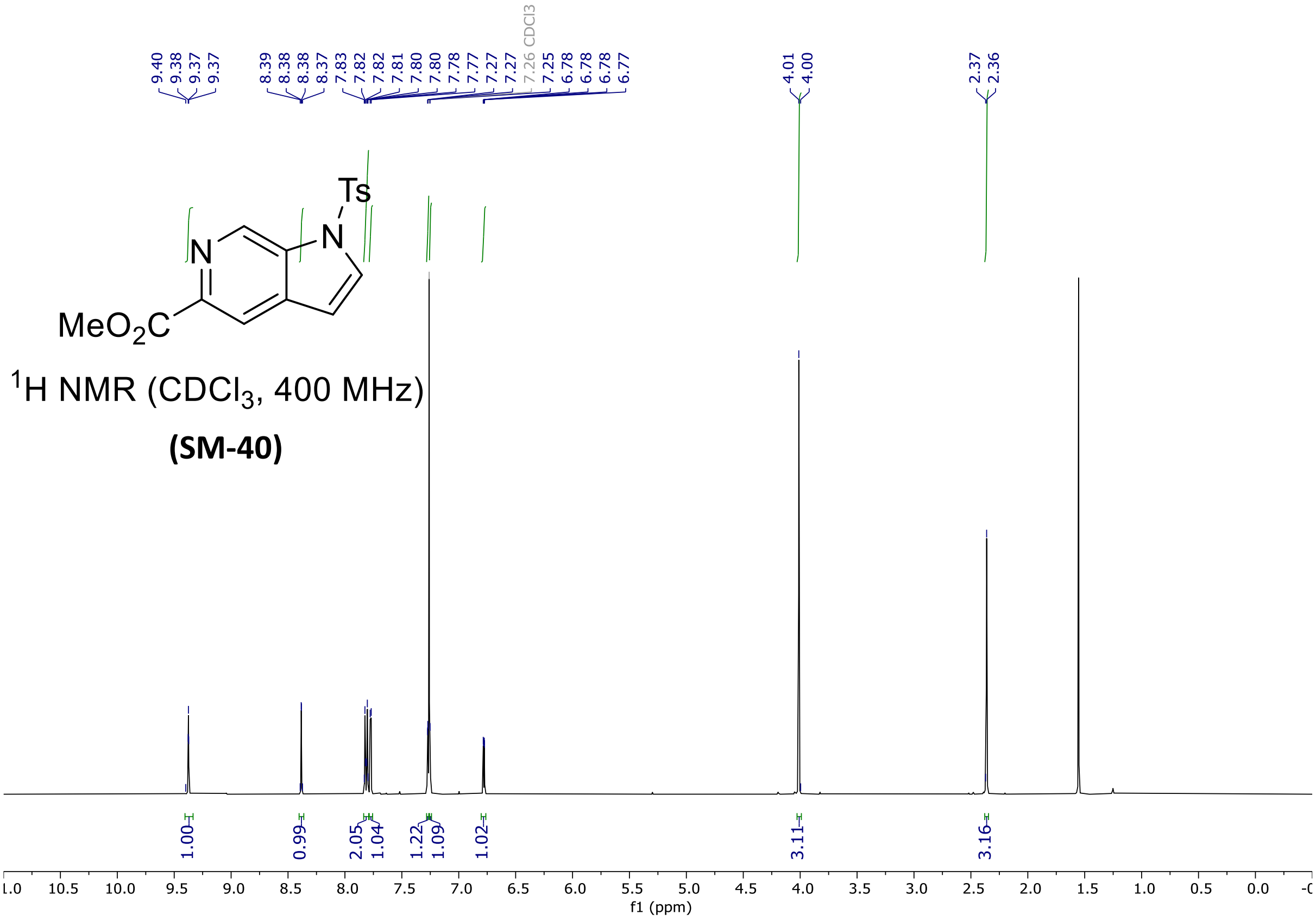

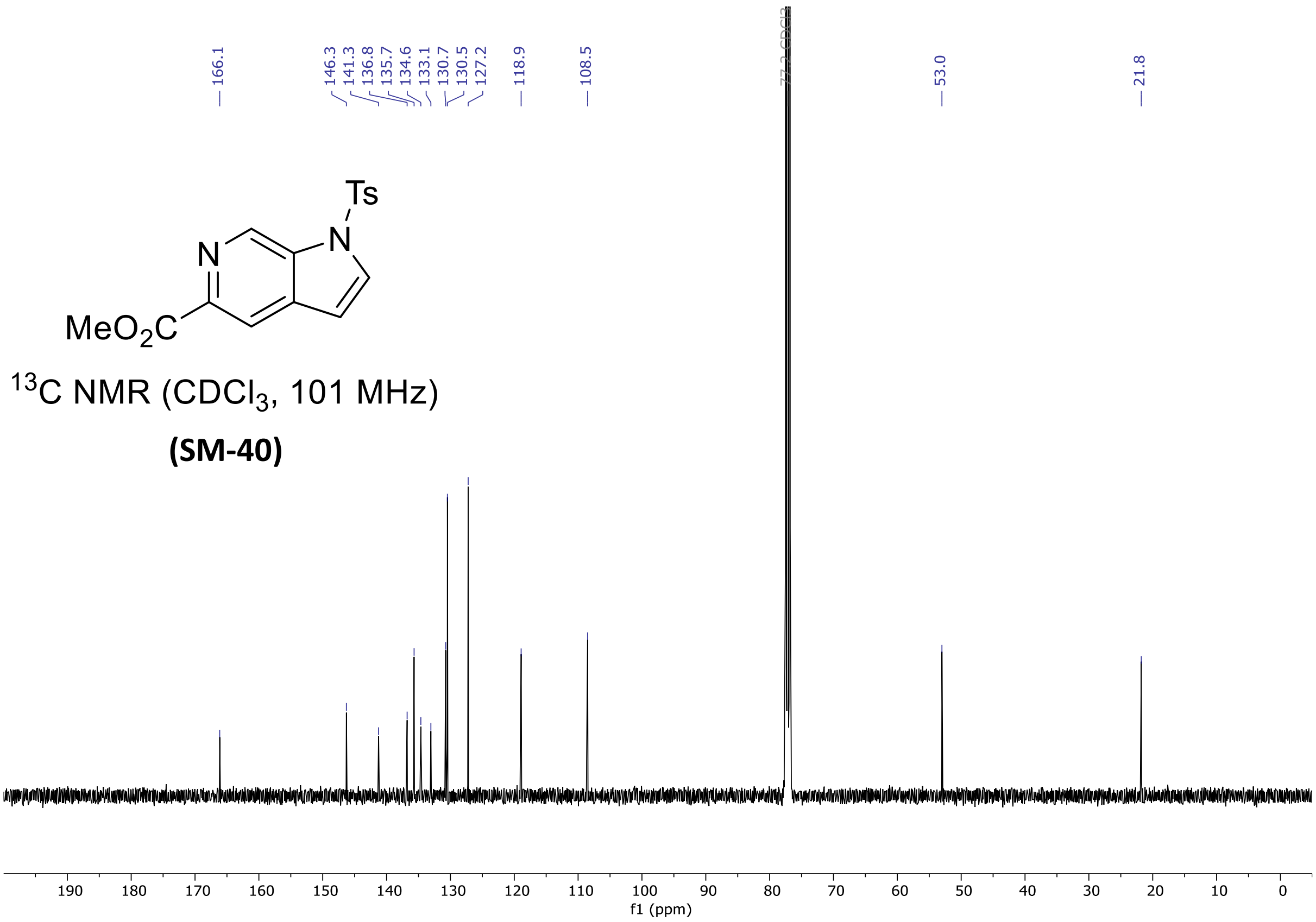

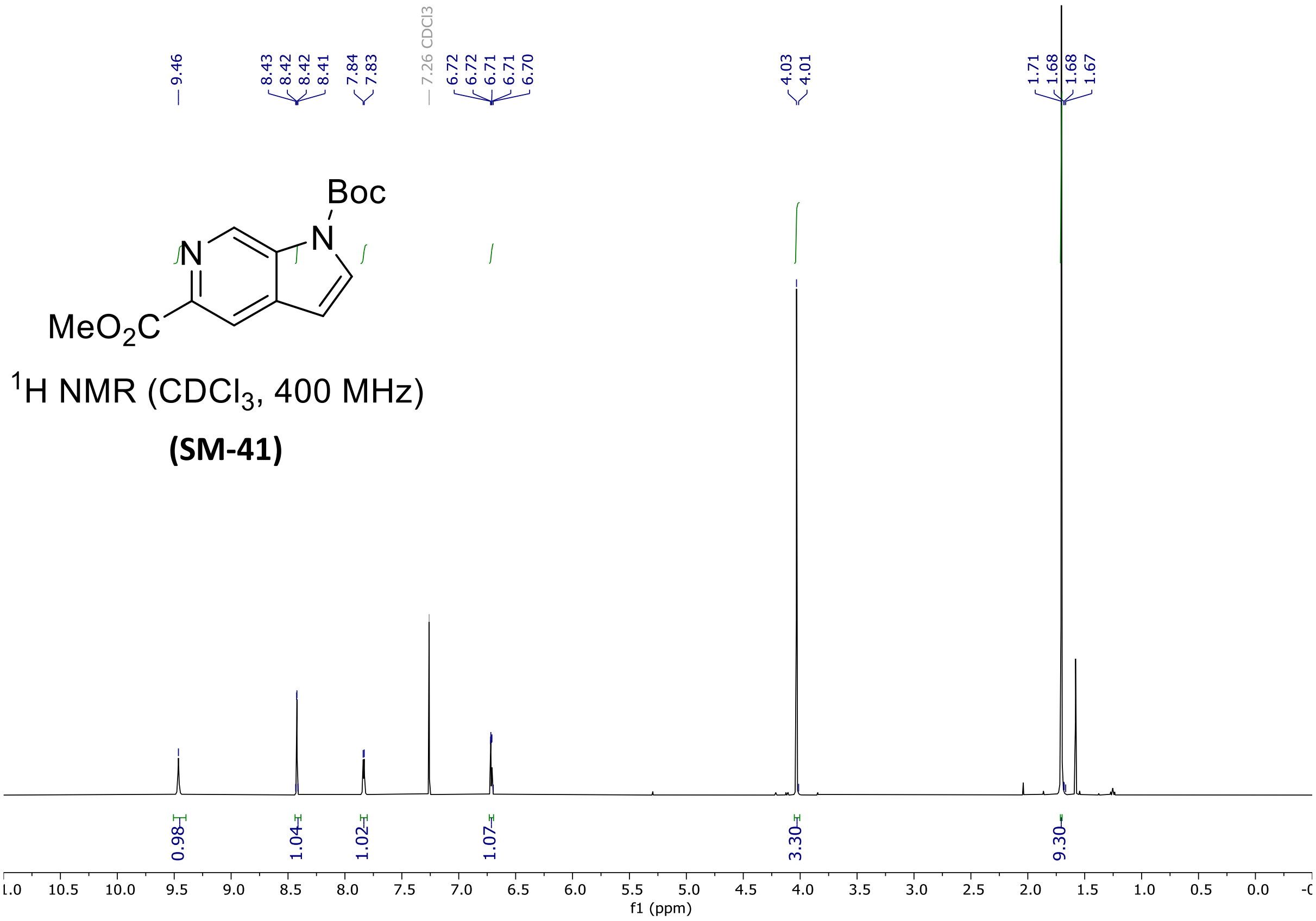
Boc
N
N
MeO2C
1H NMR (CDCl3, 400 MHz)
(SM-41)
9.46
8.43
8.42
8.42
8.41
7.84
7.83
7.26 CDCl3
6.72
6.72
6.71
6.71
6.70
4.03
4.01
1.71
1.68
1.68
1.67
0.98
1.04
1.02
1.07
3.30
9.30
f1 (ppm)

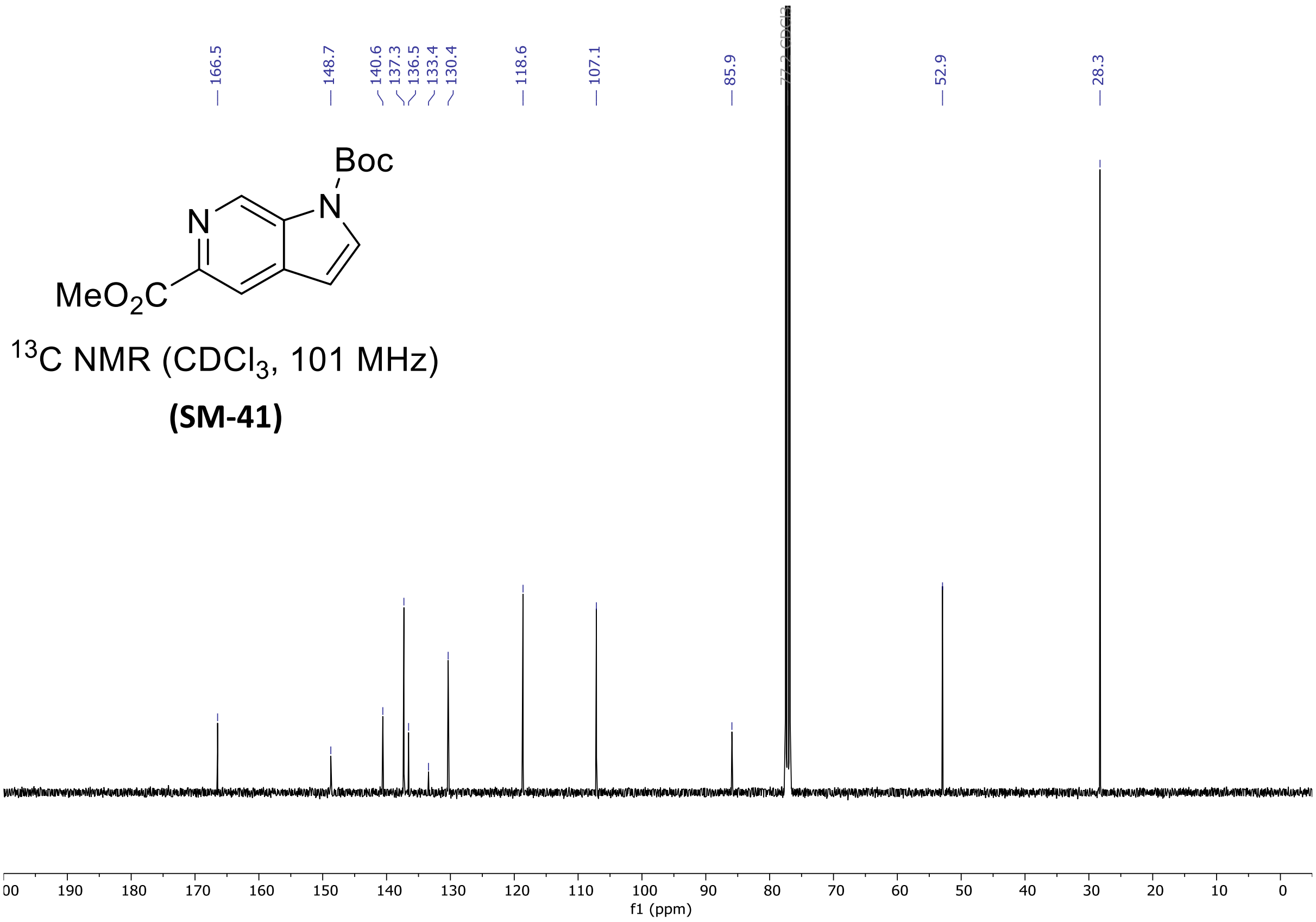
Boc
N
N
MeO2C
13C NMR (CDCl3, 101 MHz)
(SM-41)
166.5
148.7
140.6
137.3
136.5
133.4
130.4
118.6
107.1
85.9
52.9
28.3
f1 (ppm)

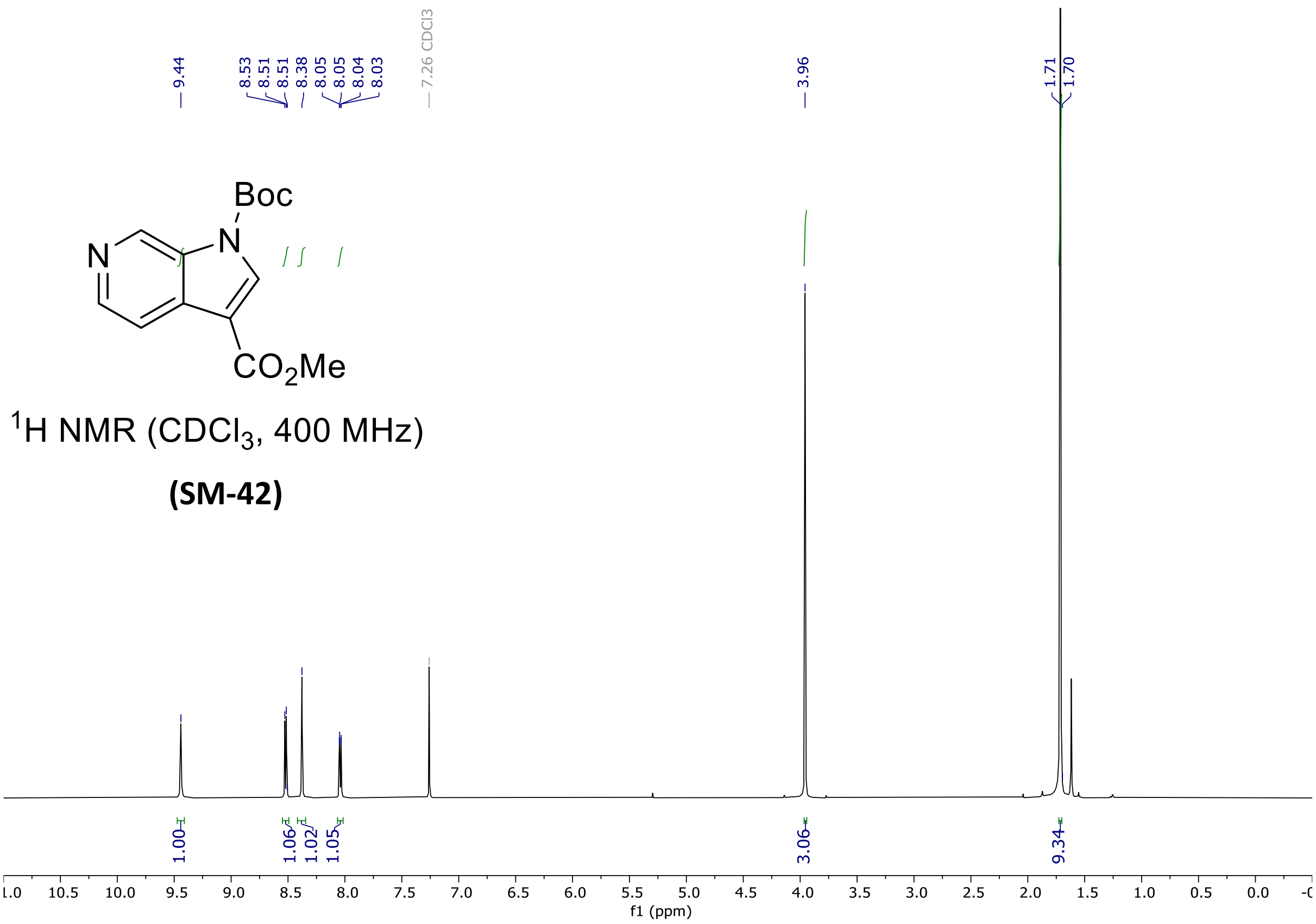
Boc
N
N
CO2Me
1H NMR (CDCl3, 400 MHz)
(SM-42)
9.44
8.53
8.51
8.51
8.38
8.05
8.05
8.04
8.03
7.26 CDCl3
3.96
1.71
1.70
1.00
1.06
1.02
1.05
3.06
9.34
f1 (ppm)

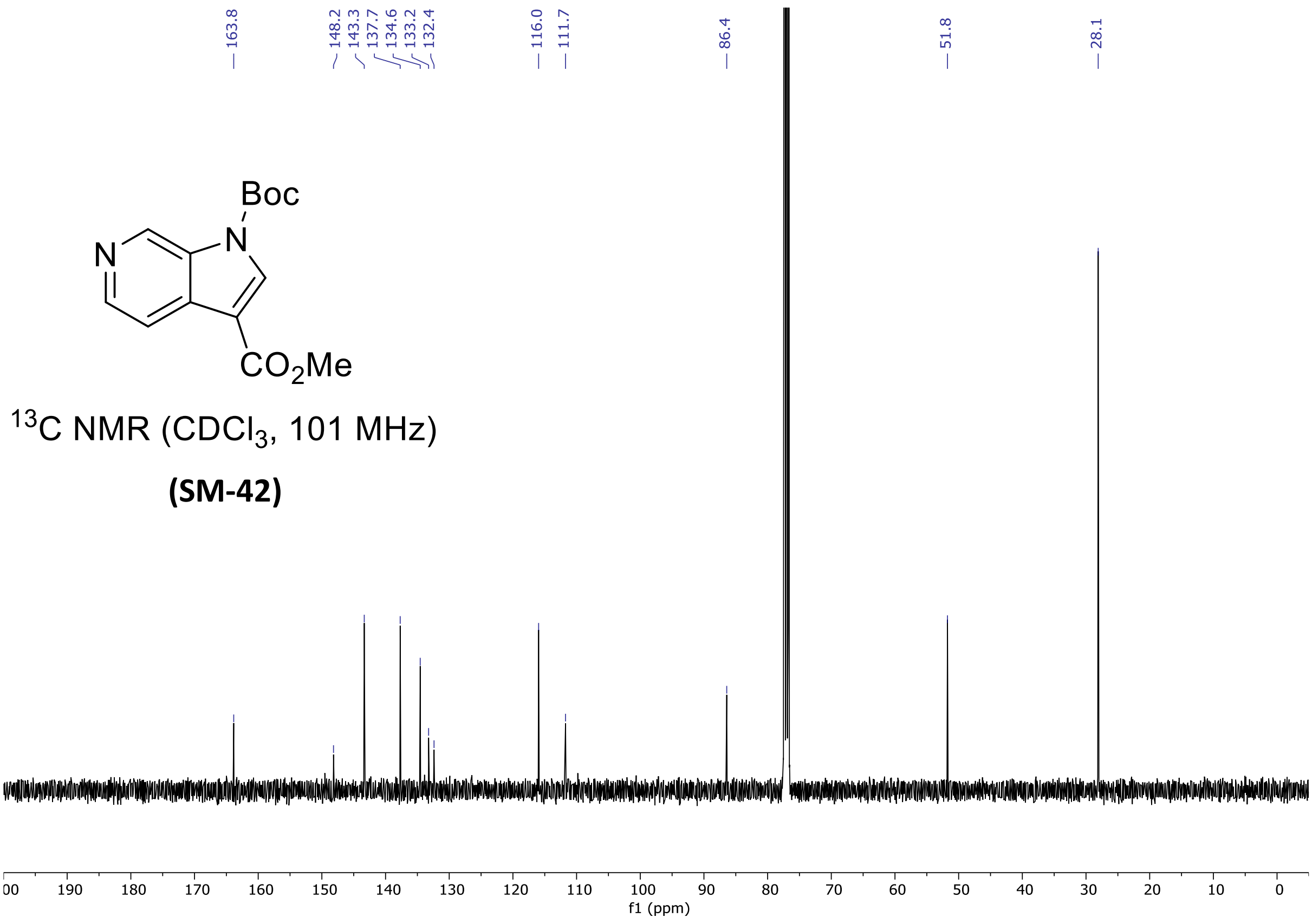
Boc
N
N
CO2Me
13C NMR (CDCl3, 101 MHz)
(SM-42)
163.8
148.2
143.3
137.7
134.6
133.2
132.4
116.0
111.7
86.4
51.8
28.1
f1 (ppm)

Pyrazines hydrogenation

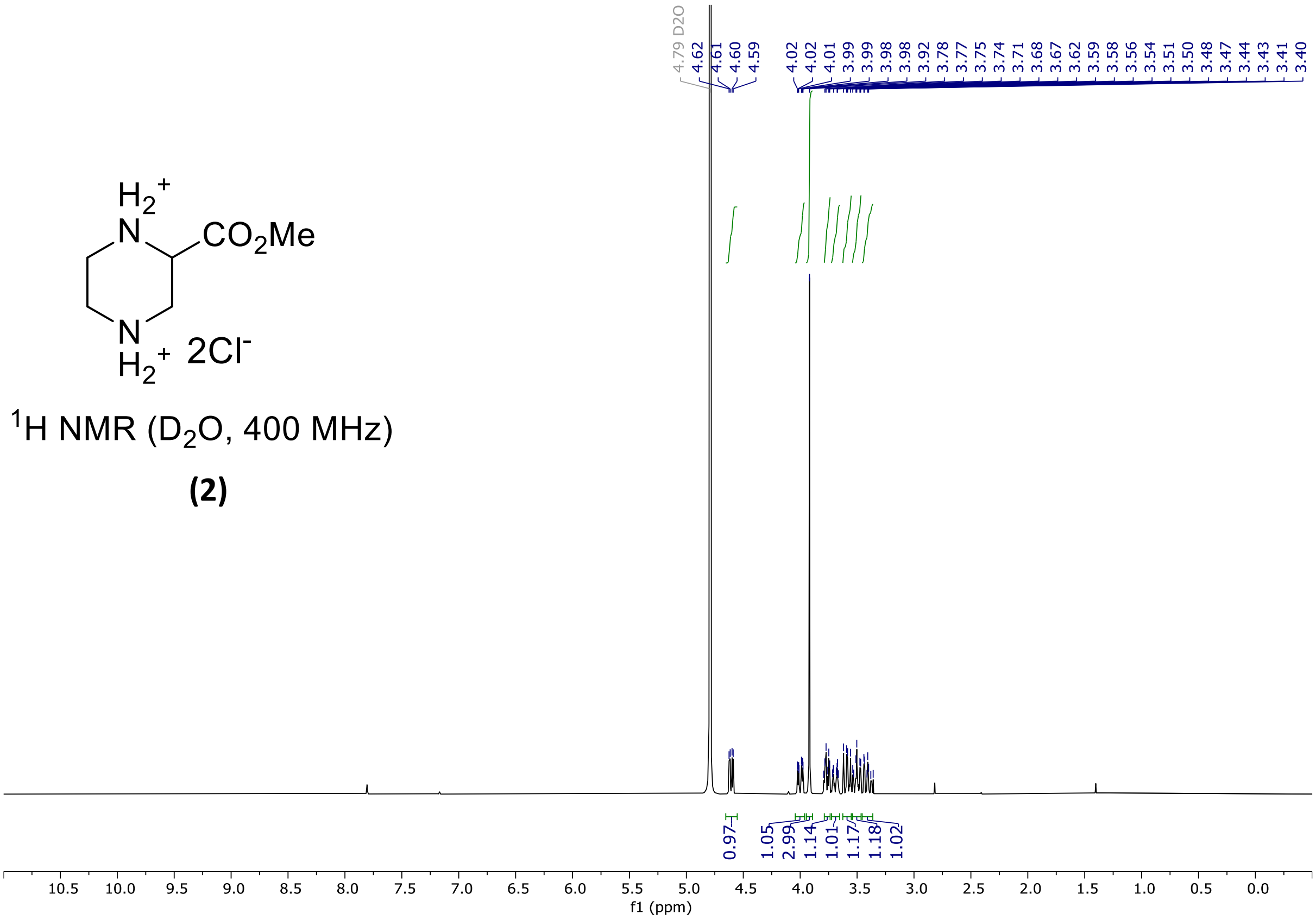

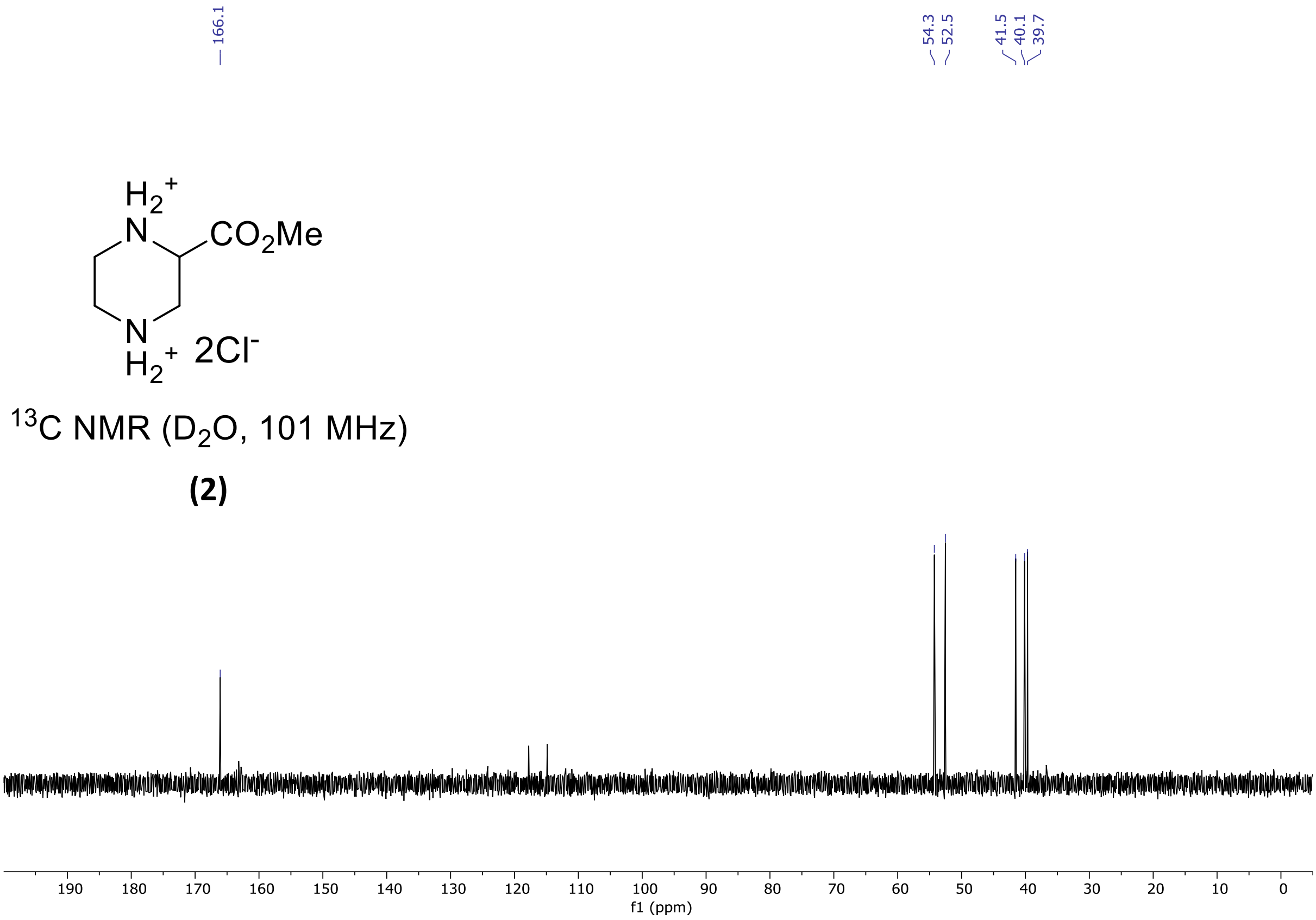

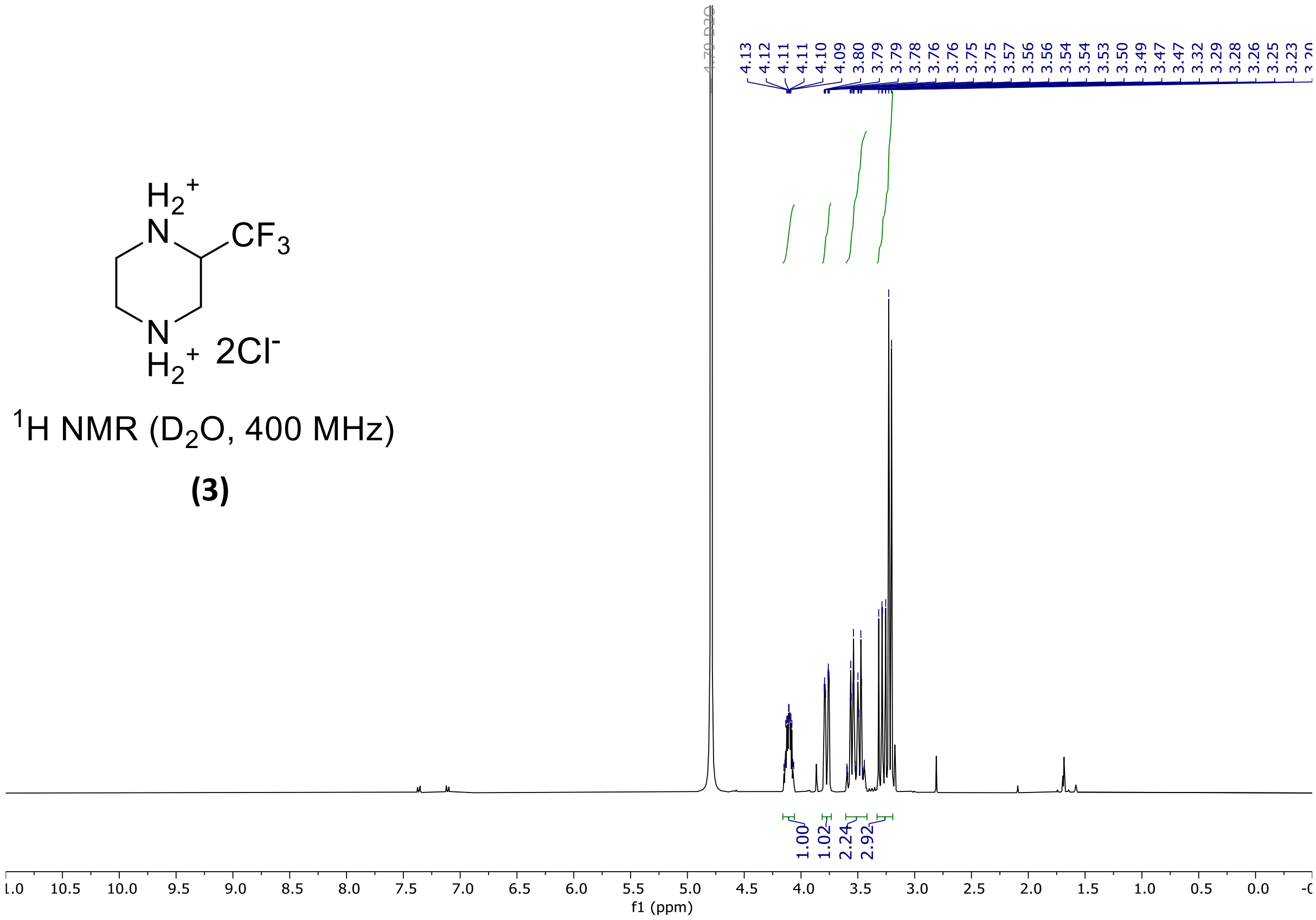

^{1}H NMR (D_2O, 400 MHz)

(3)

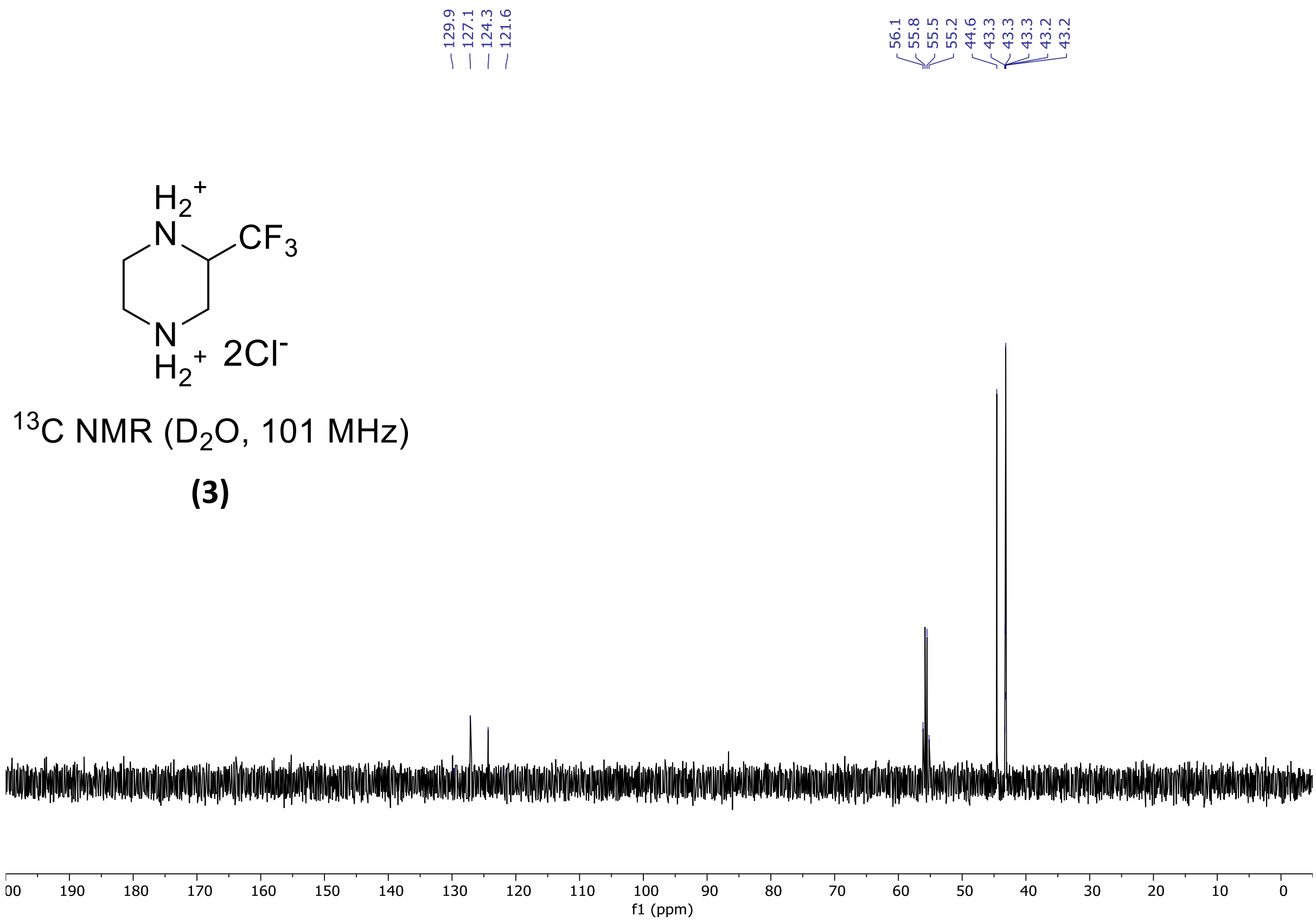

^{13}C NMR (D_2O, 101 MHz)

(3)

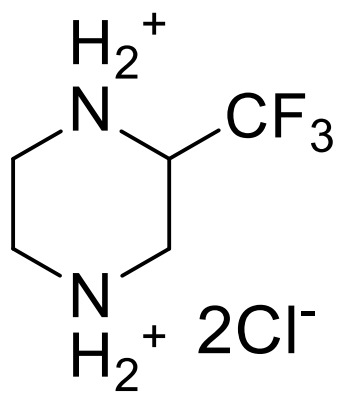

^{19}F NMR (D_2O, 376 MHz)

(3)

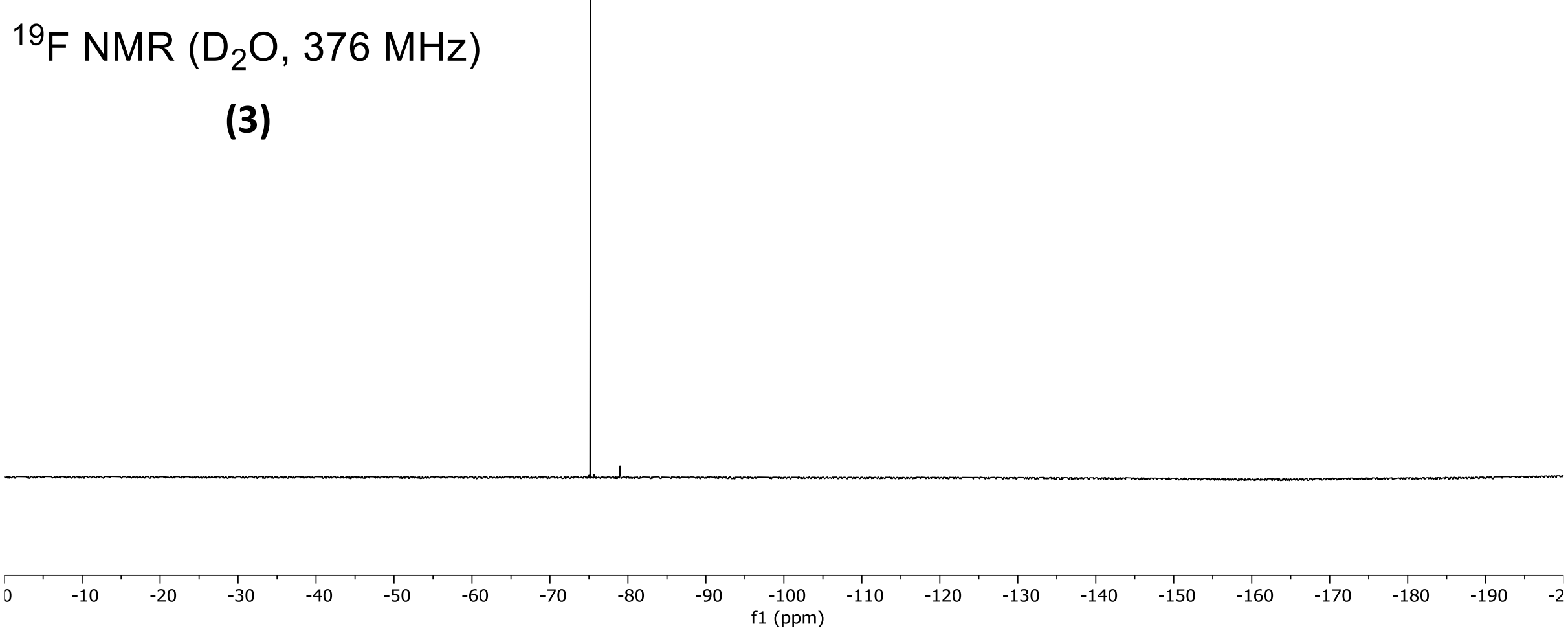

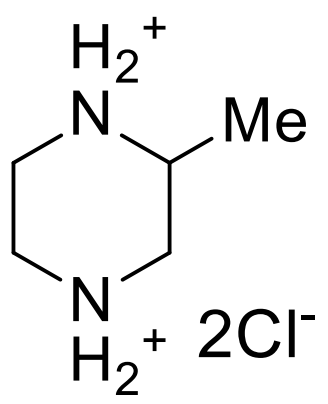

^{1}H NMR (D_2O, 400 MHz)

(4)

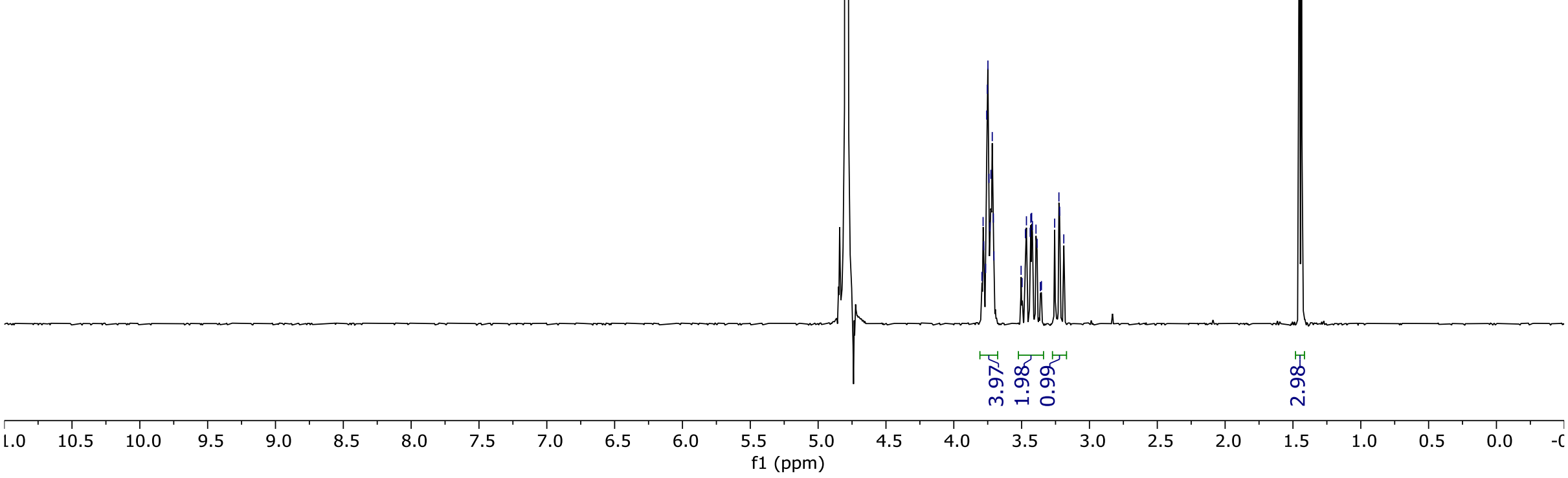

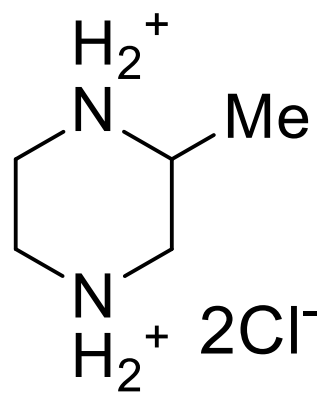

^{13}C NMR (D_2O, 101 MHz)

(4)

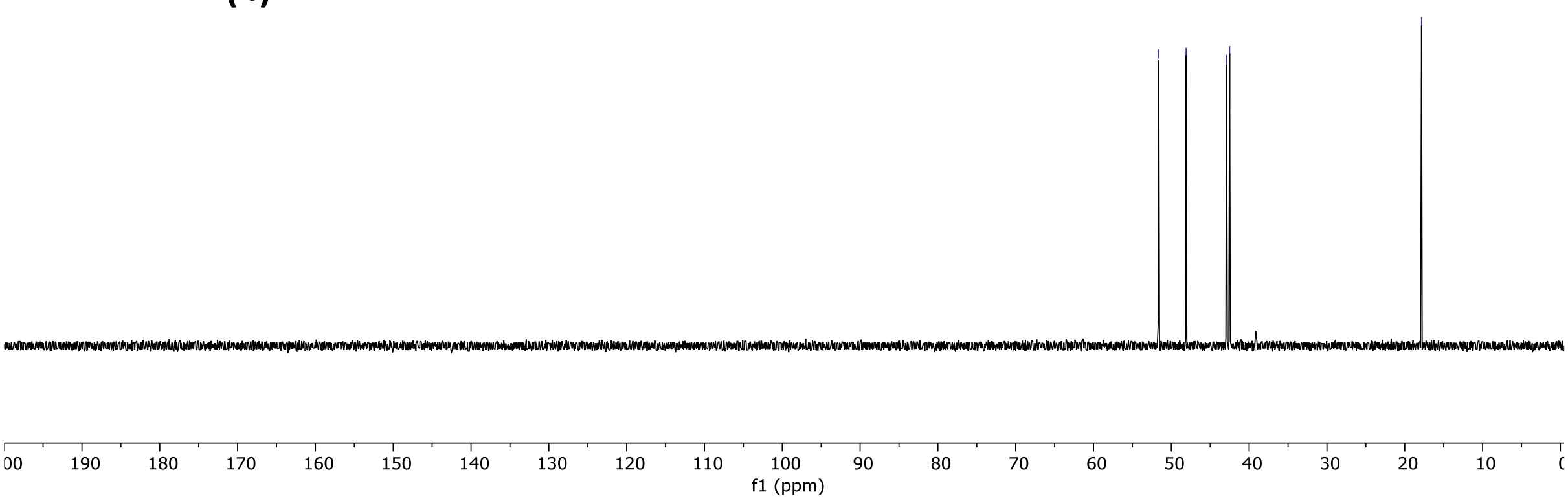

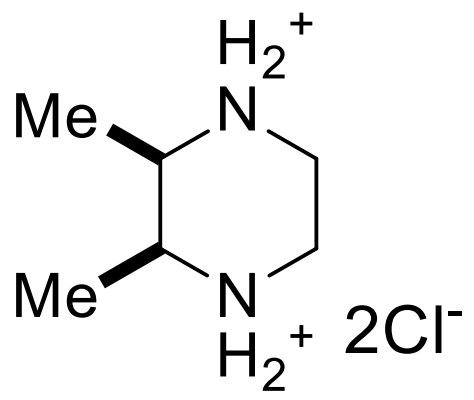

^{1}H NMR (D_2O, 400 MHz)

(5)

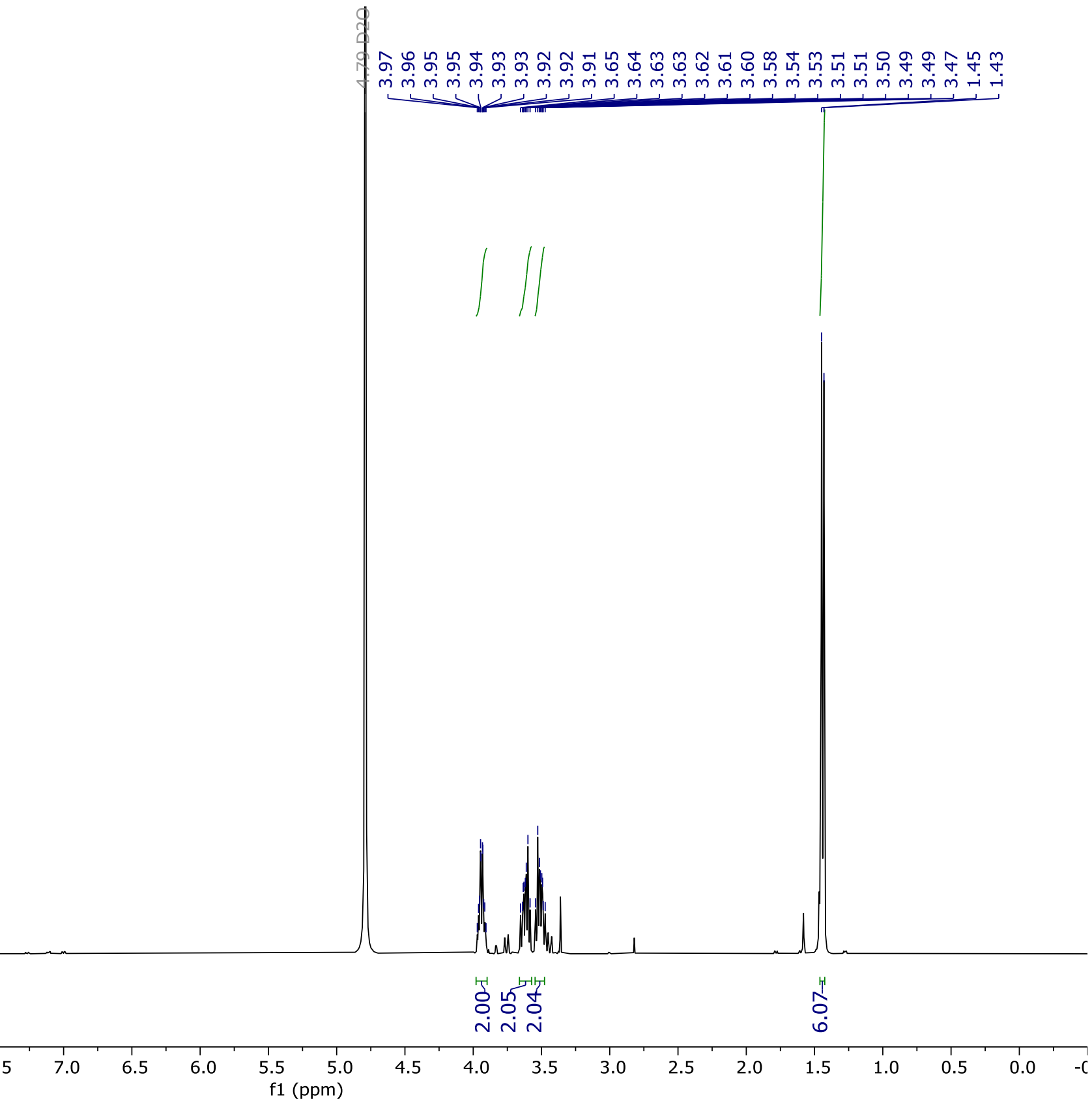

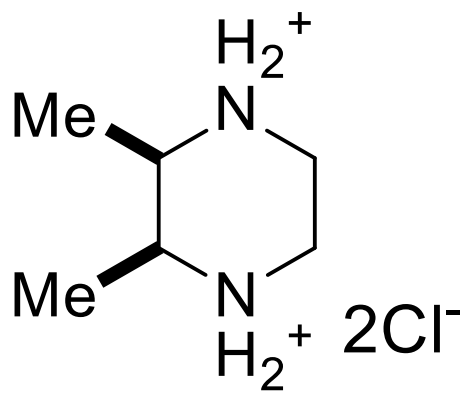

^{13}C NMR (D_2O, 101 MHz)

(5)

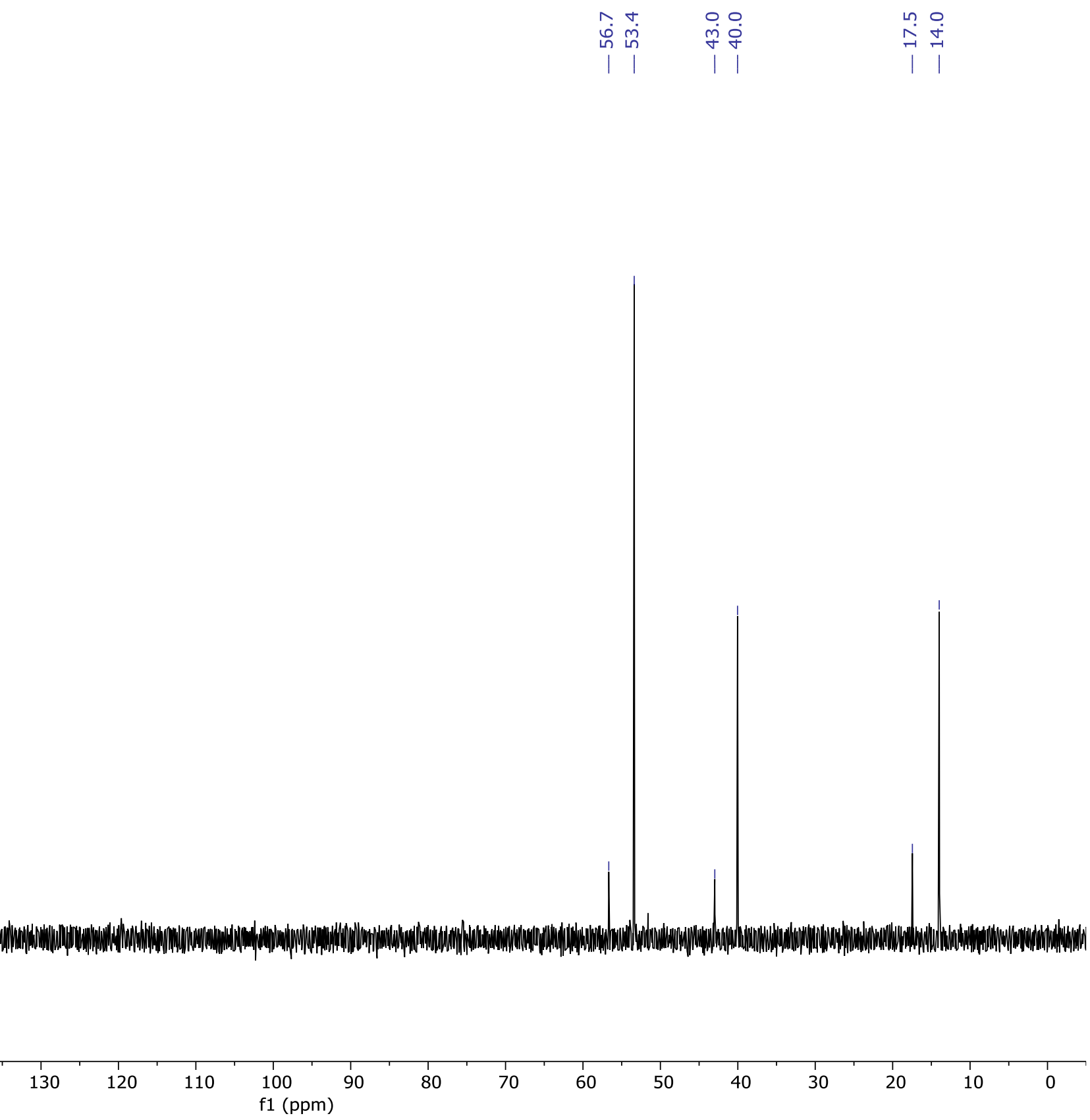

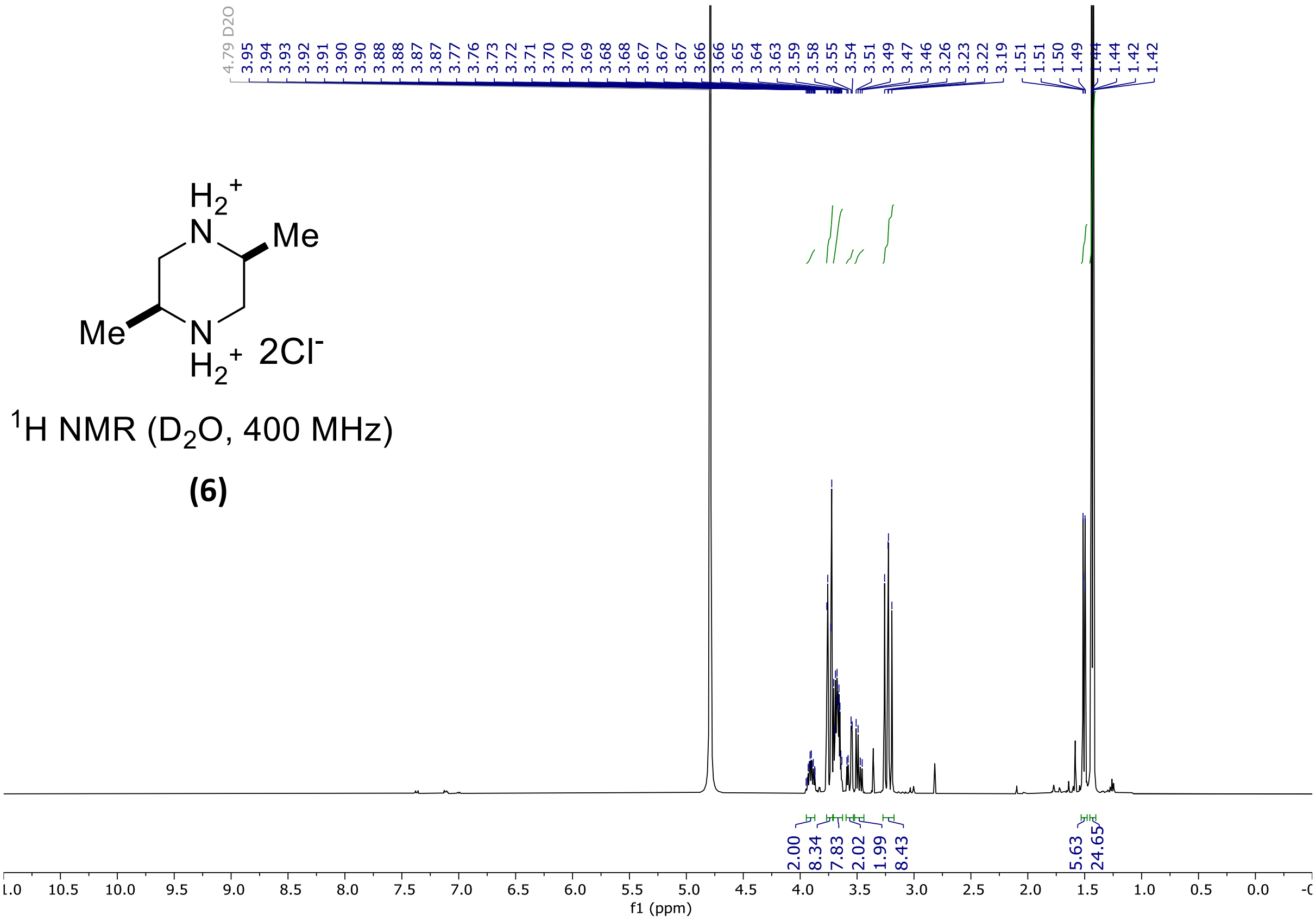

4.79 D2O
3.95 3.94 3.93 3.92 3.91 3.90 3.88 3.88 3.87 3.87 3.77 3.76 3.73 3.72 3.71 3.70 3.70 3.69 3.68 3.67 3.67 3.67 3.66 3.66 3.65 3.64 3.63 3.59 3.58 3.55 3.54 3.51 3.49 3.47 3.46 3.26 3.23 3.22 3.19 1.51 1.51 1.50 1.49 1.44 1.44 1.42 1.42
H_2^+
N
Me
Me
N
H_2^+ 2Cl$^-$
^{1}H NMR (D_2O, 400 MHz)
(6)
2.00
8.34
7.83
2.02
1.99
8.43
5.63
24.65
f1 (ppm)

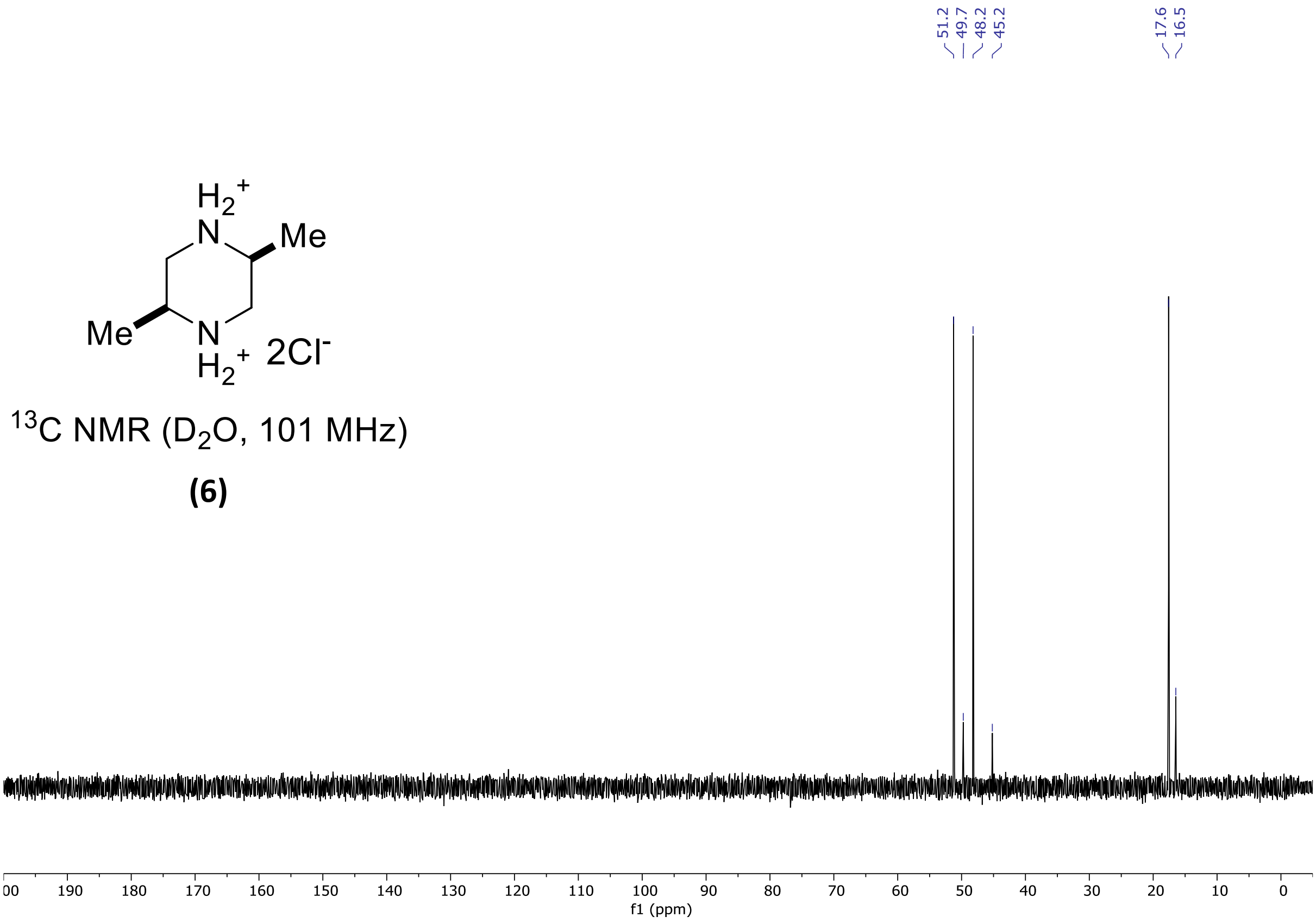

51.2
49.7
48.2
45.2
17.6
16.5
H_2^+
N
Me
Me
N
H_2^+ 2Cl$^-$
^{13}C NMR (D_2O, 101 MHz)
(6)
f1 (ppm)

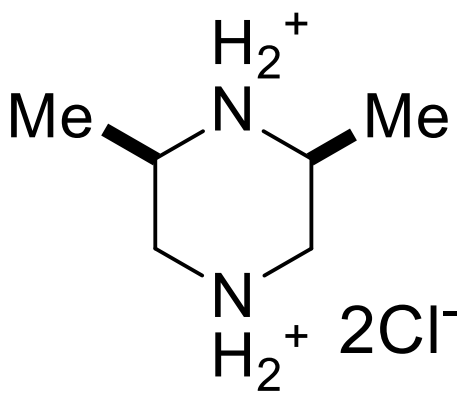

^{1}H NMR (D_2O, 400 MHz)

(7)

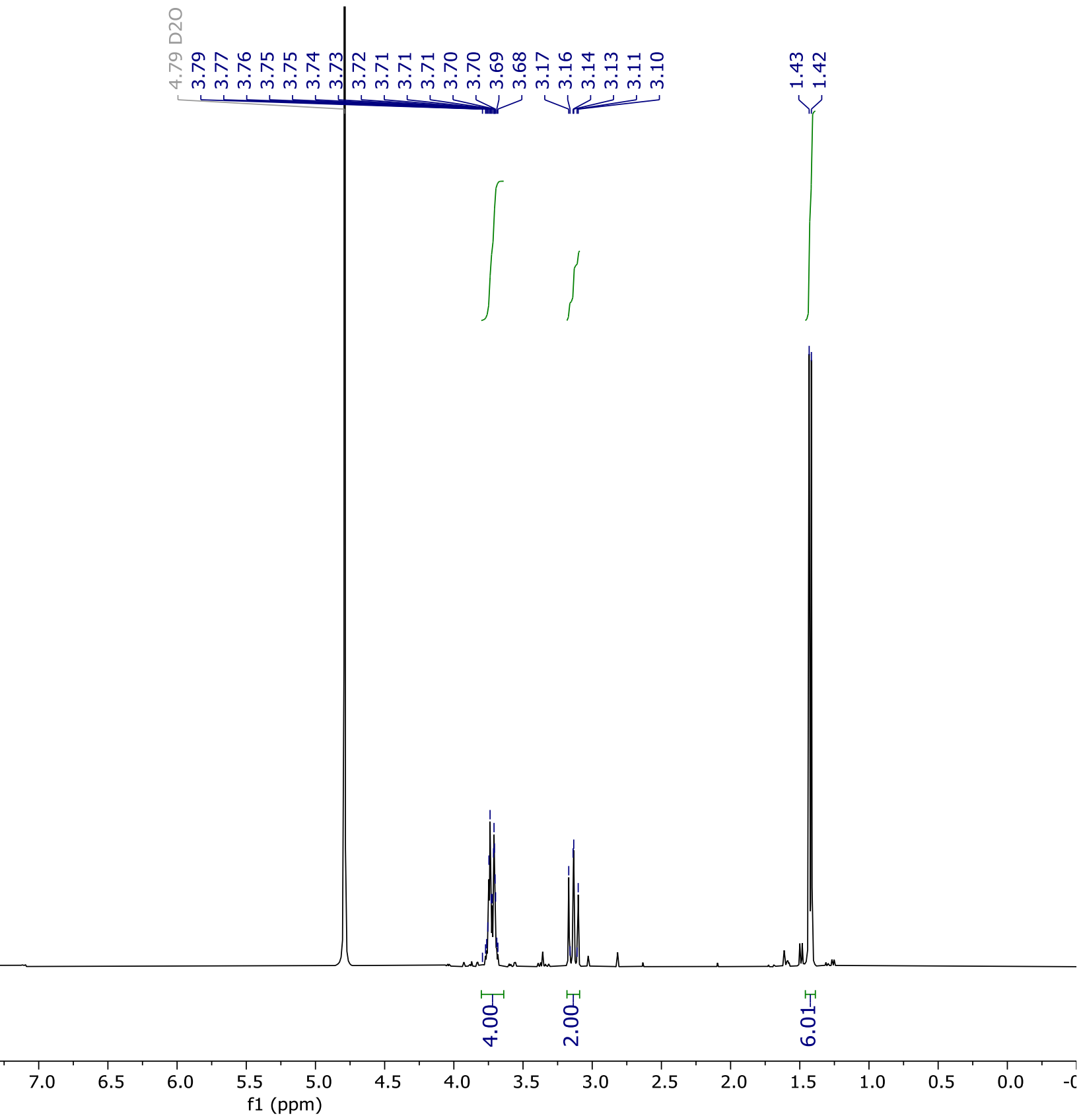

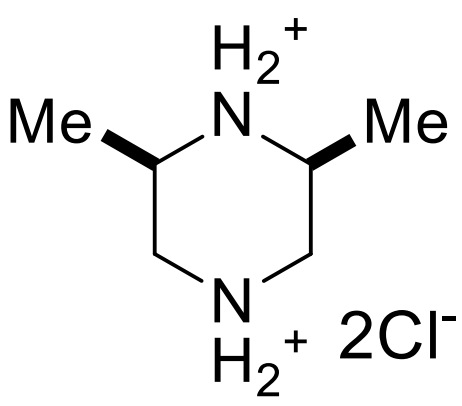

^{13}C NMR (D_2O, 101 MHz)

(7)

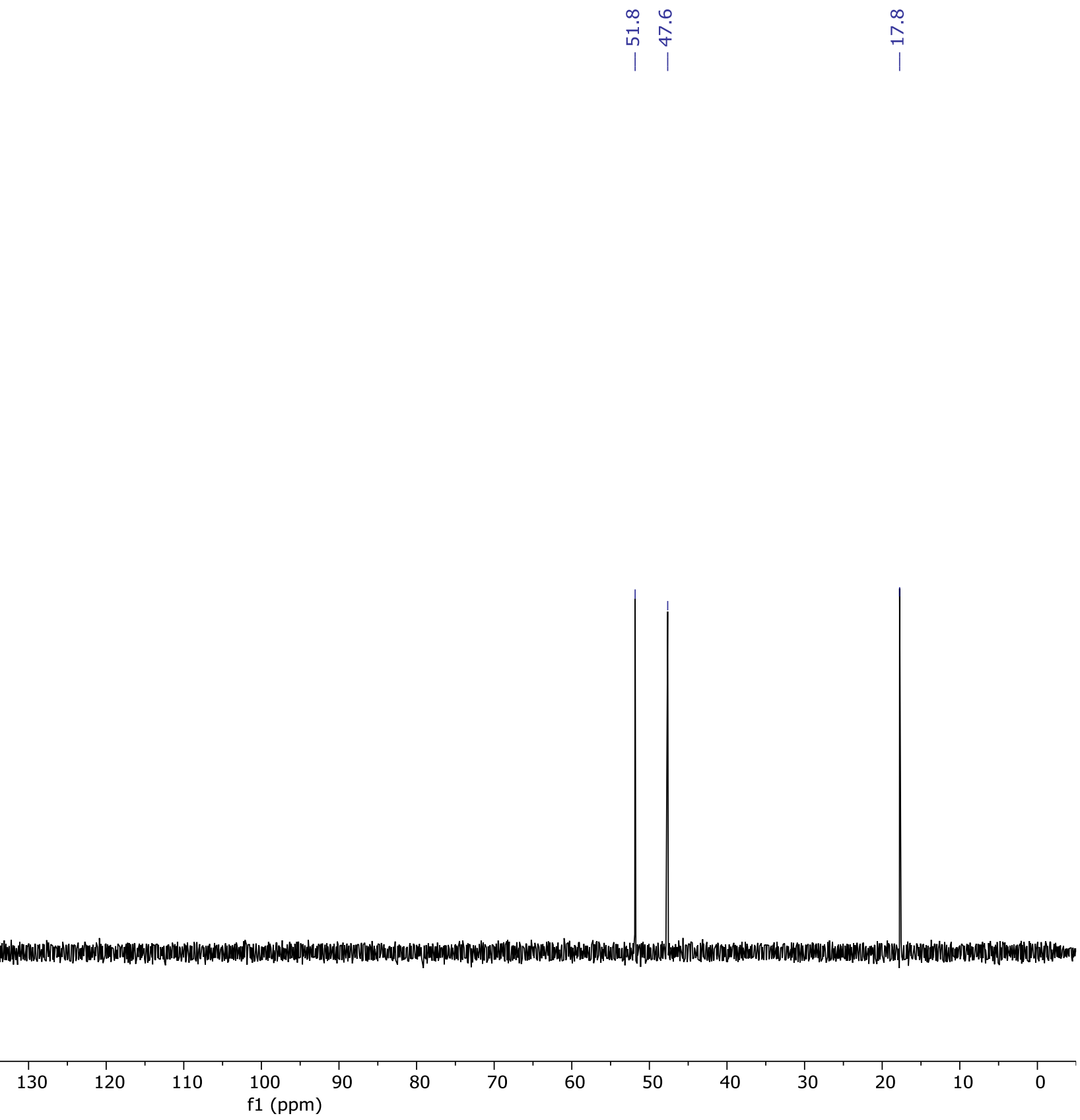

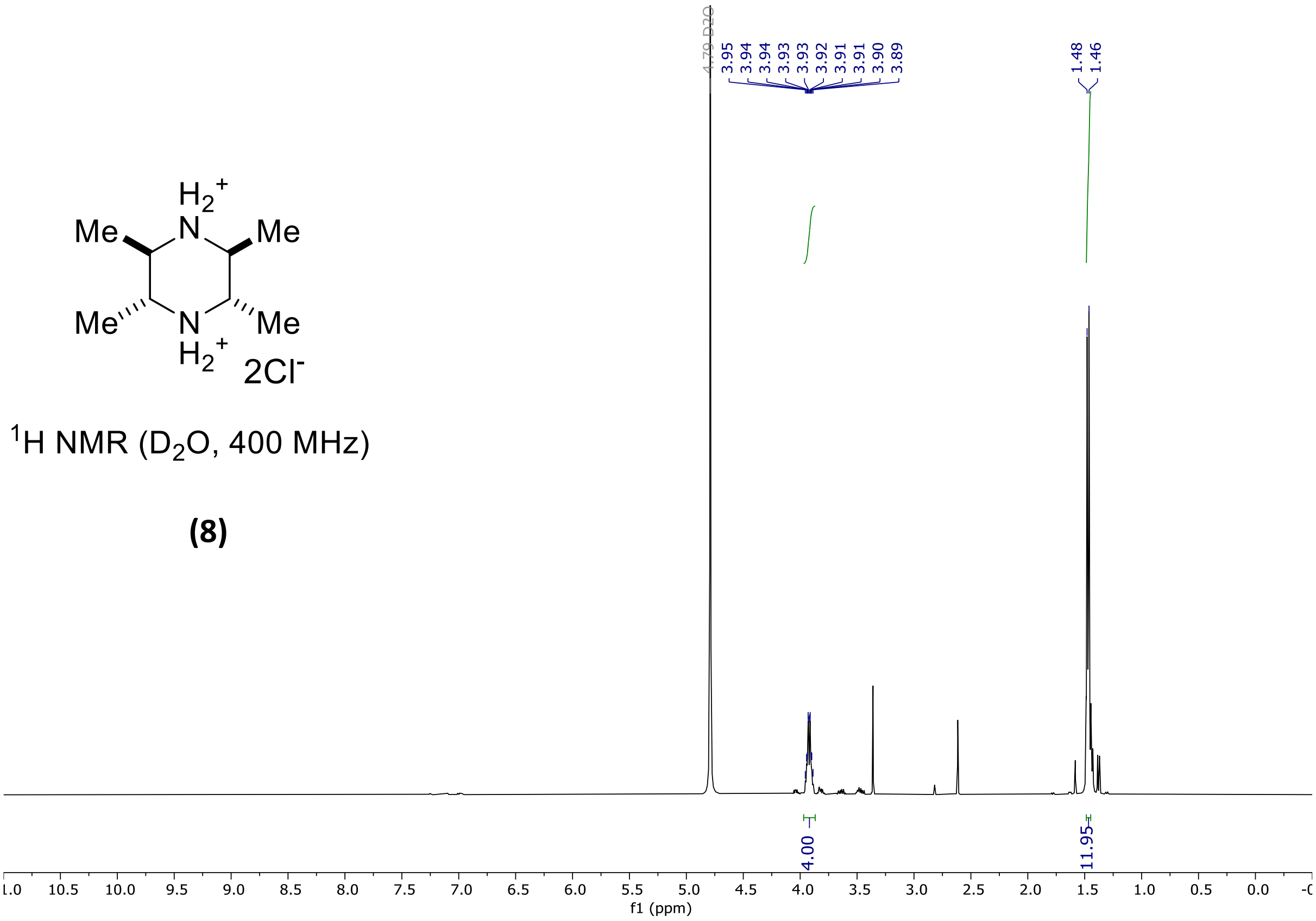

^{1}H NMR (D_2O, 400 MHz)

(8)

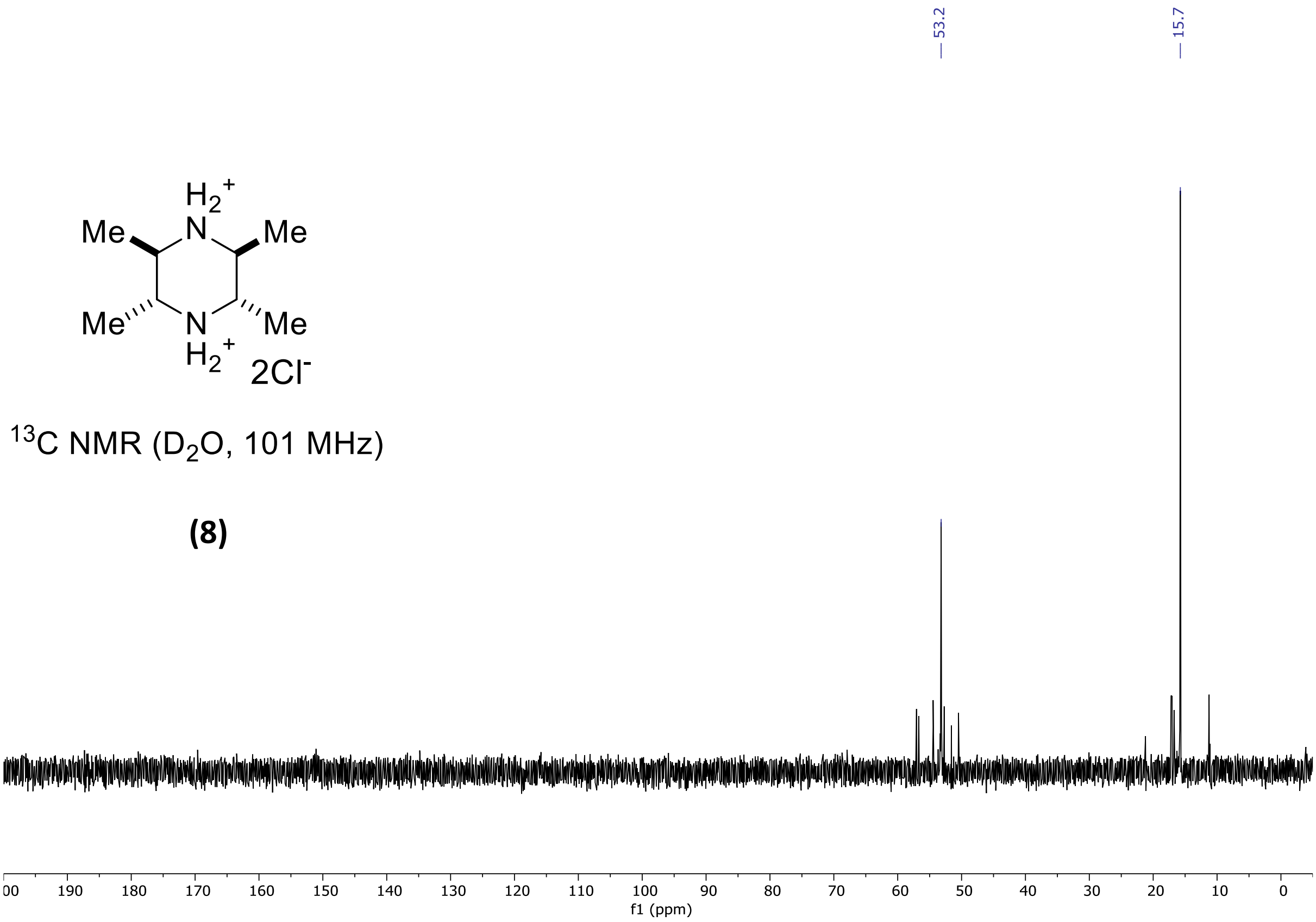

^{13}C NMR (D_2O, 101 MHz)

(8)

Pyrimidines hydrogenation

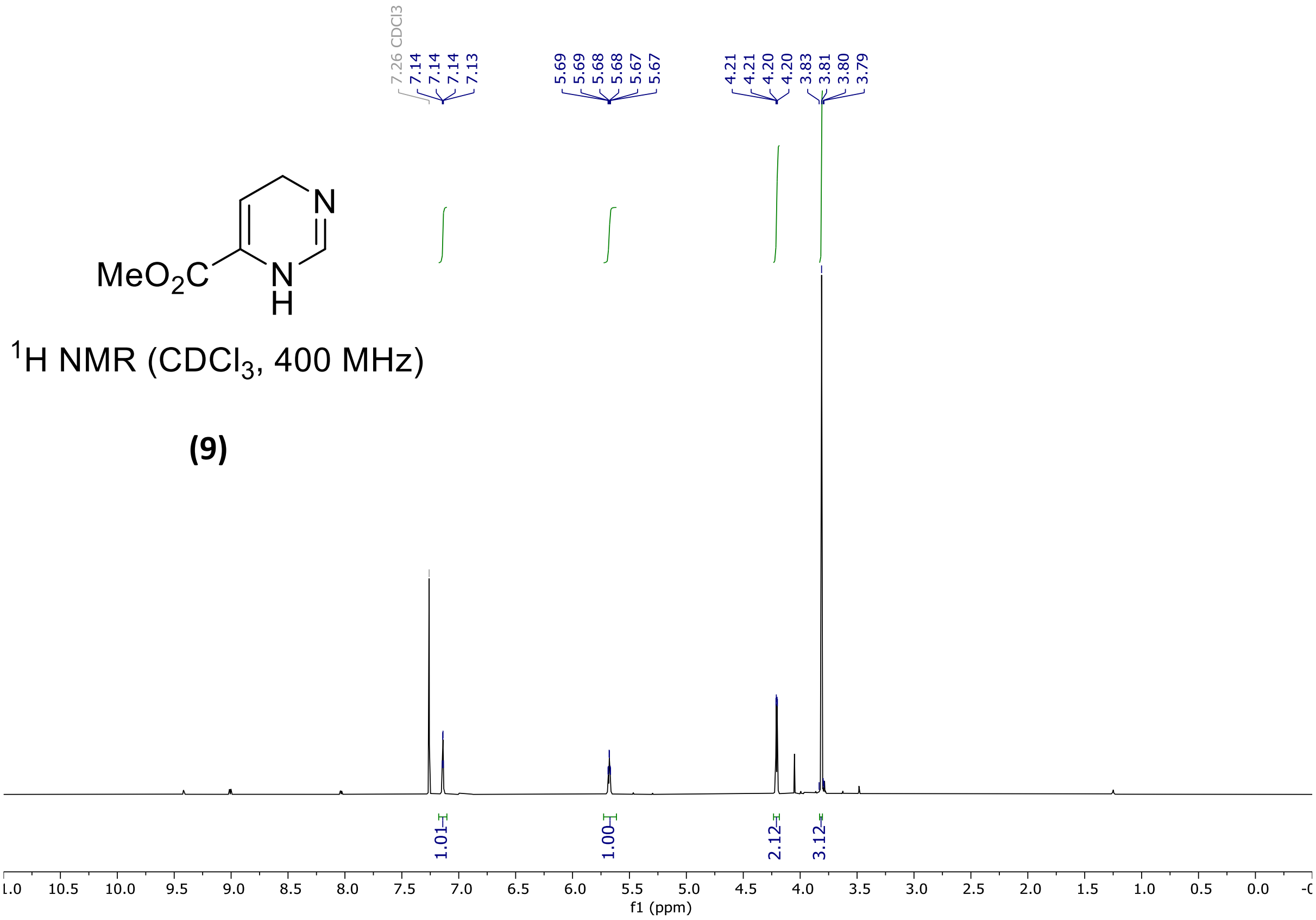

^{1}H NMR ($CDCl_3$, 400 MHz)

(9)

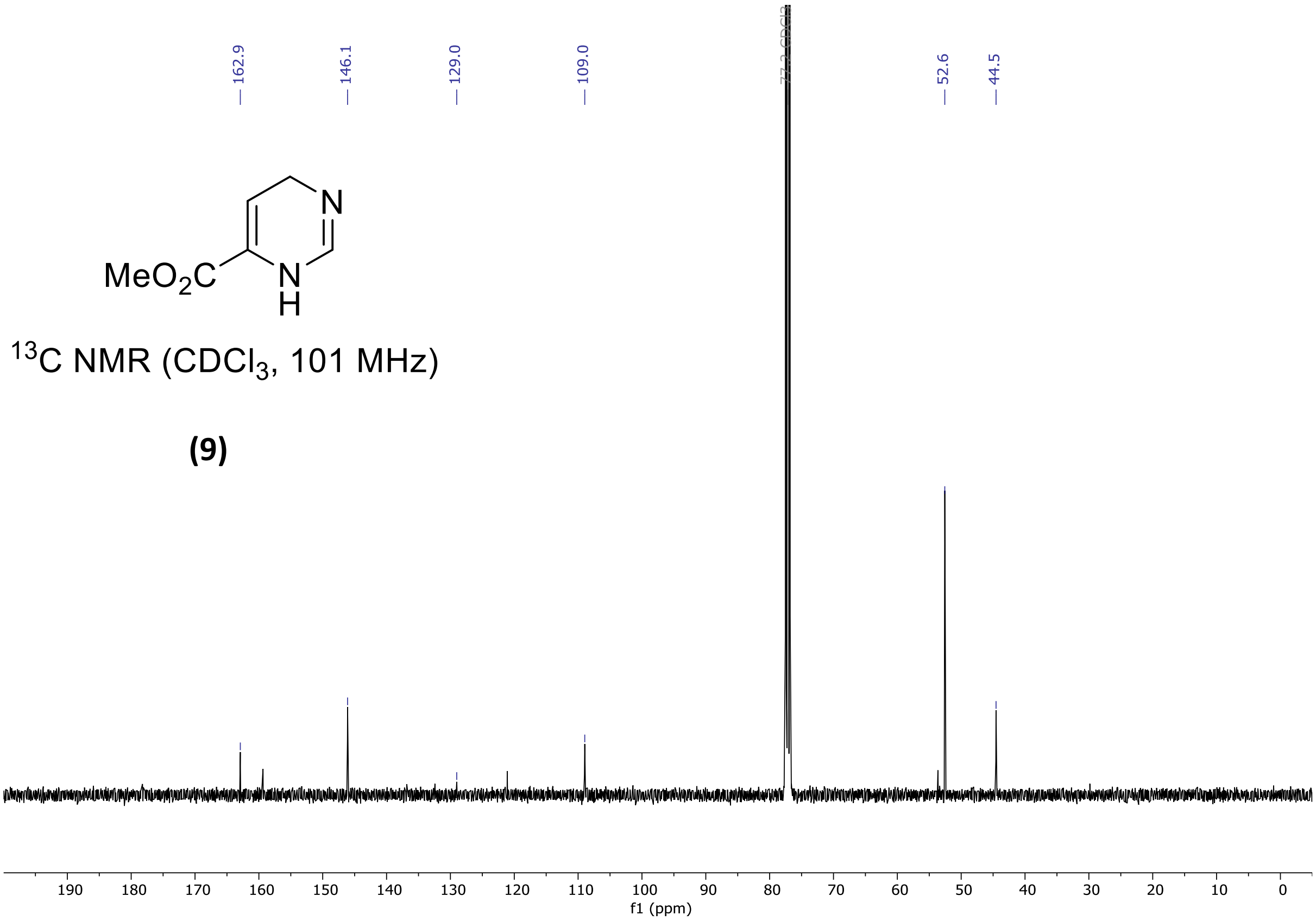

^{13}C NMR ($CDCl_3$, 101 MHz)

(9)

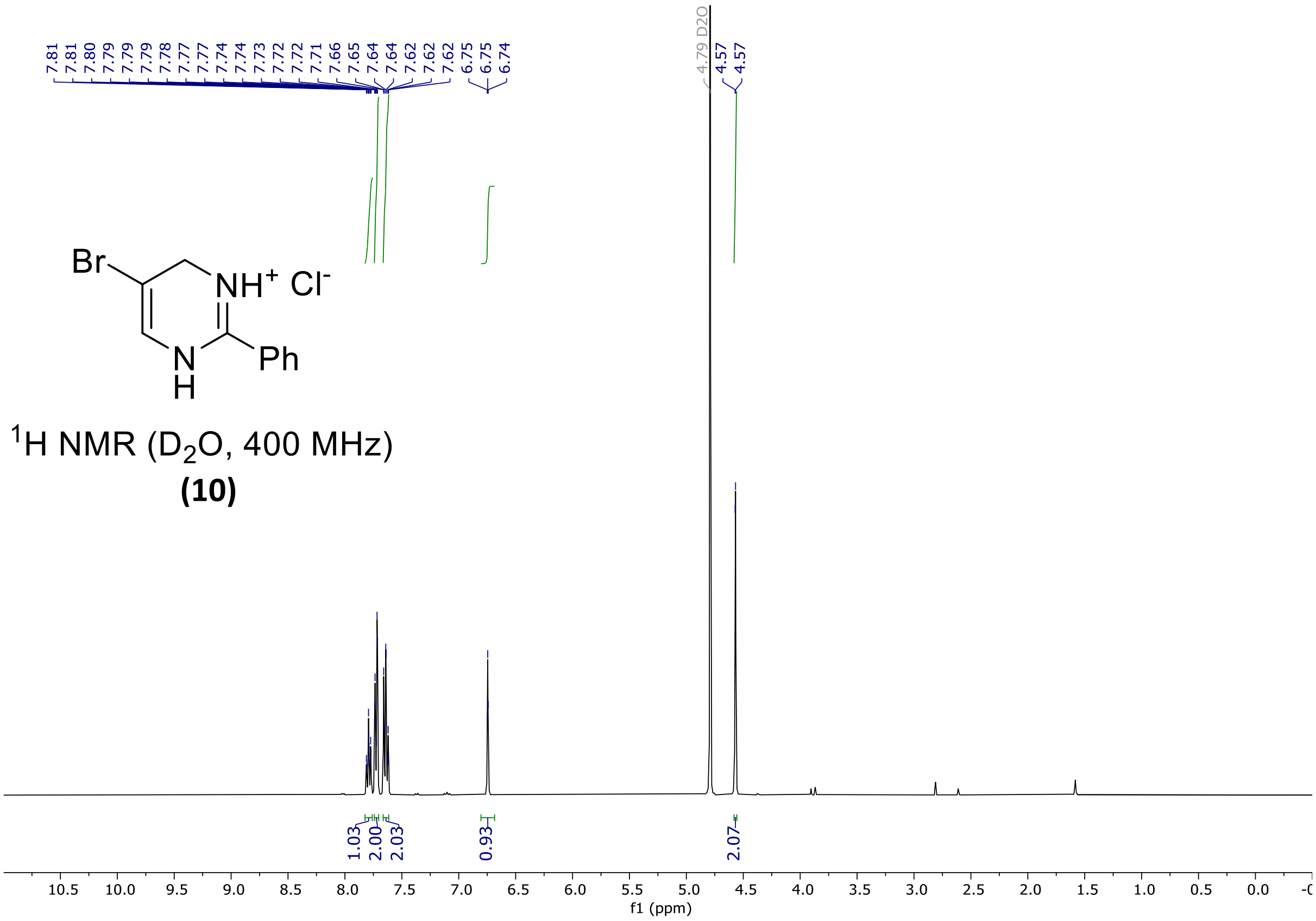

^{1}H NMR (D_2O, 400 MHz)

(10)

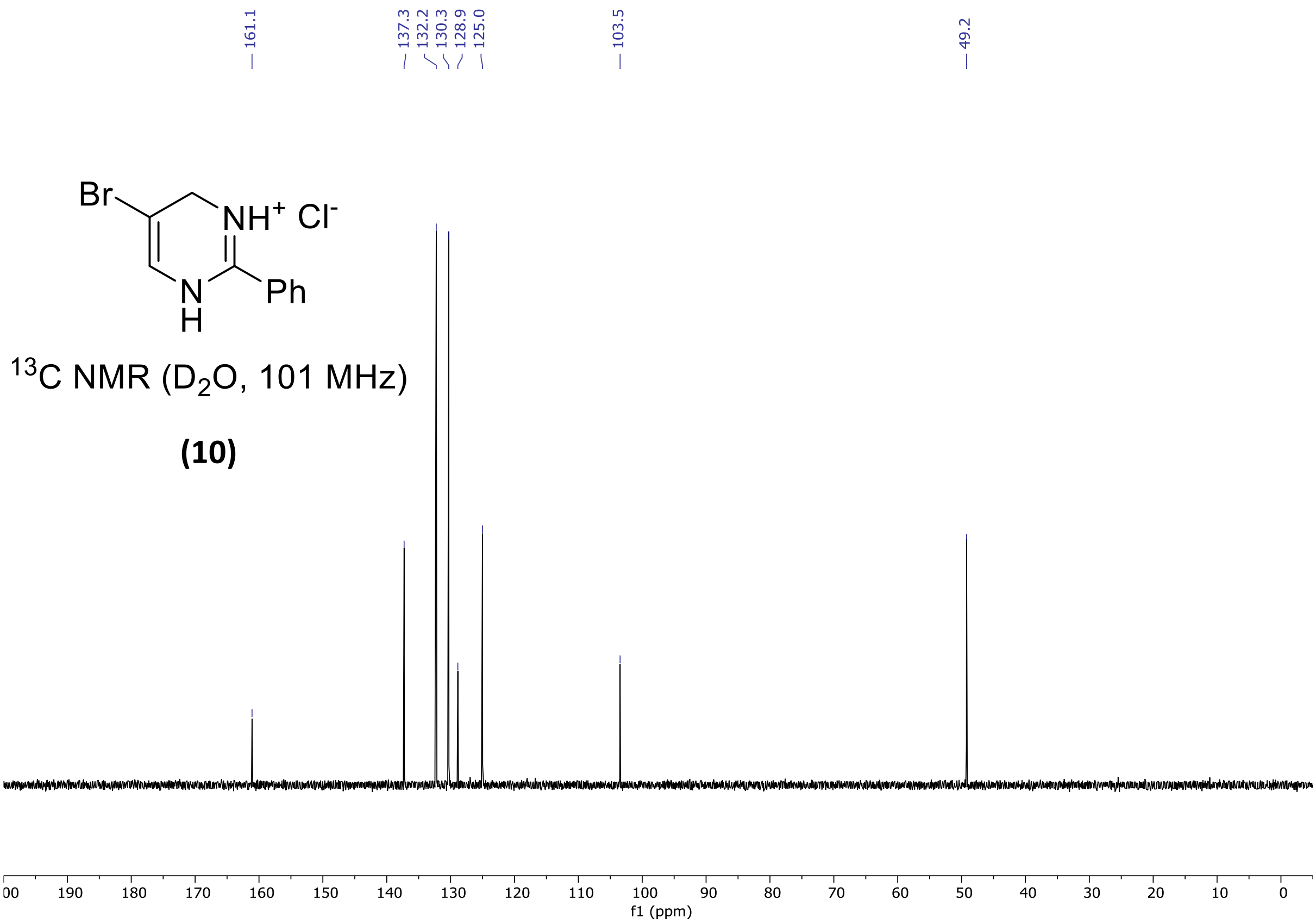

^{13}C NMR (D_2O, 101 MHz)

(10)

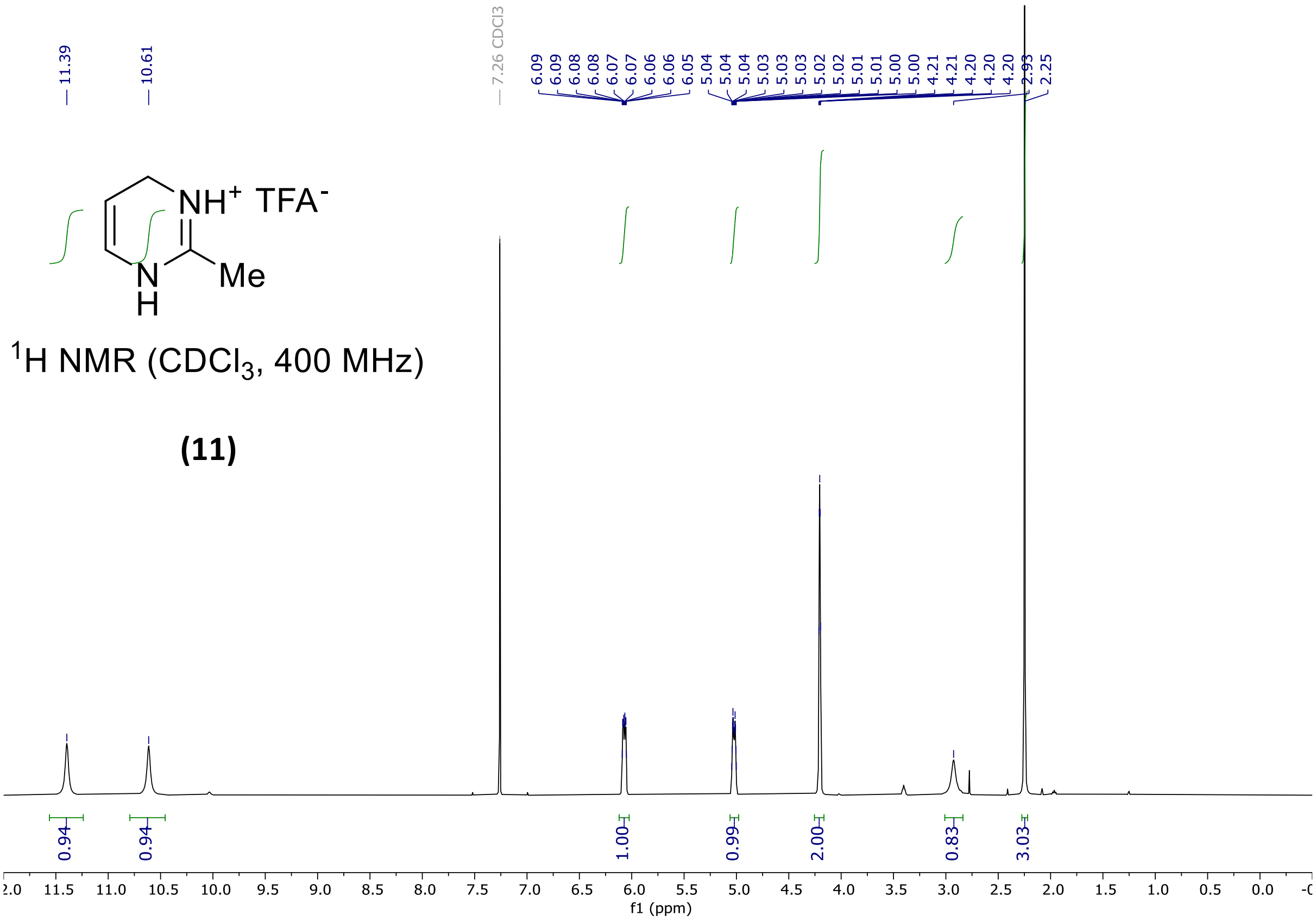
11.39
10.61
7.26 CDCl3
6.09 6.09 6.08 6.08 6.07 6.07 6.06 6.06 6.05
5.04 5.04 5.04 5.03 5.03 5.03 5.02 5.02 5.01 5.01 5.00 5.00
4.21 4.21 4.20 4.20 4.20
2.93
2.25
NH+ TFA-
N
H
Me
1H NMR (CDCl3, 400 MHz)
(11)
0.94
0.94
1.00
0.99
2.00
0.83
3.03
f1 (ppm)

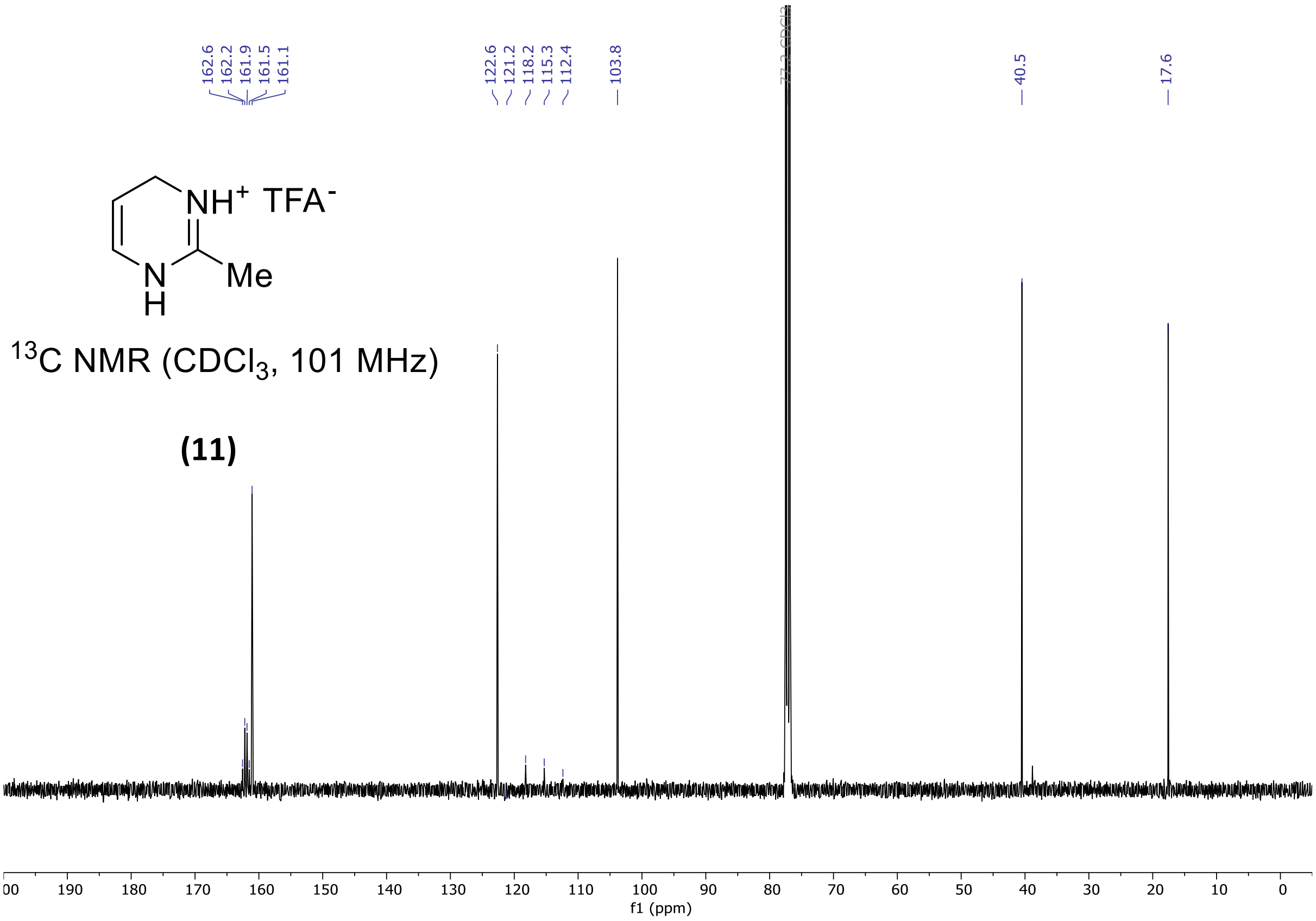
162.6 162.2 161.9 161.5 161.1
122.6 121.2 118.2 115.3 112.4
103.8
77.2 CDCl3
40.5
17.6
NH+ TFA-
N
H
Me
13C NMR (CDCl3, 101 MHz)
(11)
f1 (ppm)

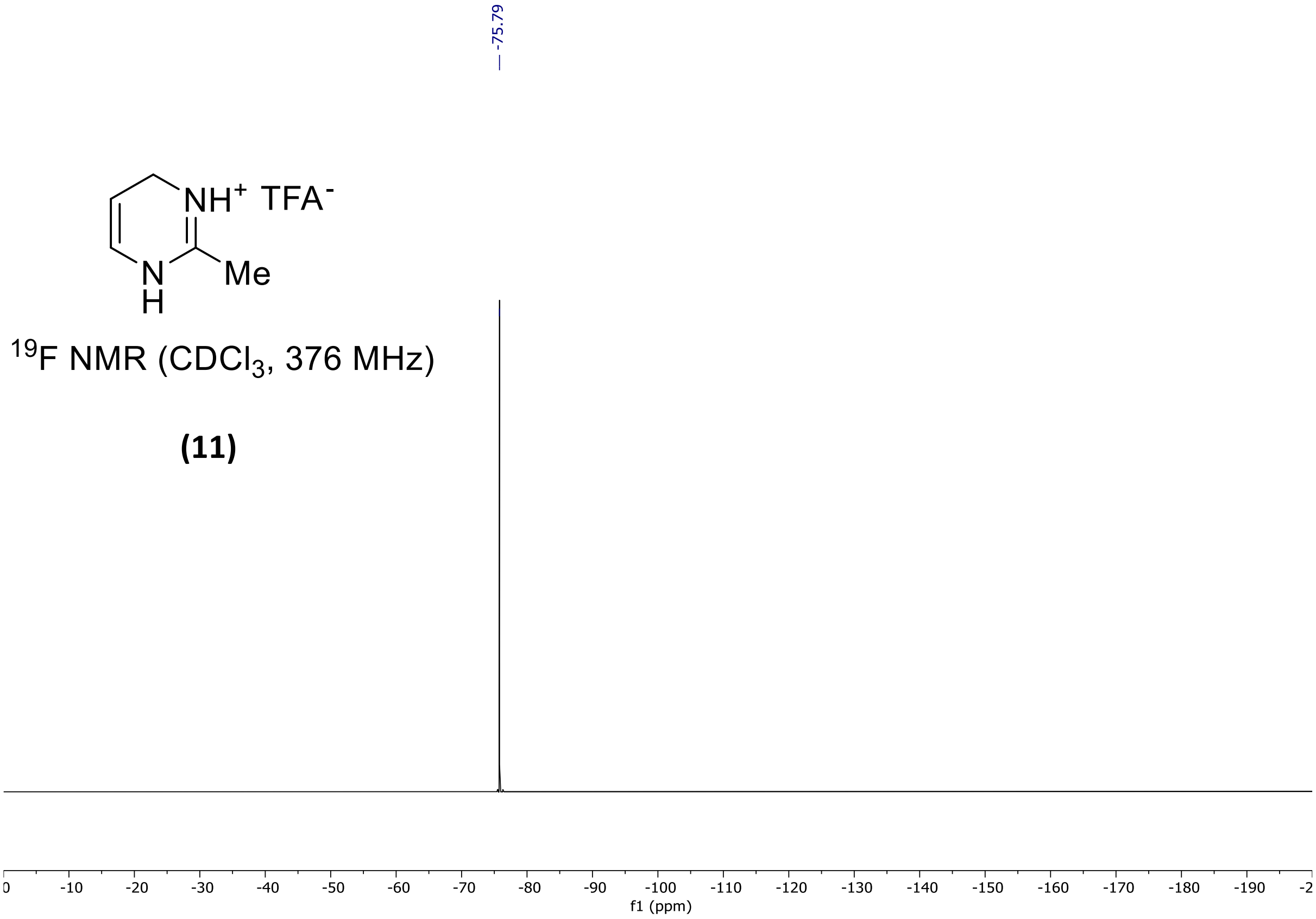

^{19}F NMR ($CDCl_3$, 376 MHz)

(11)

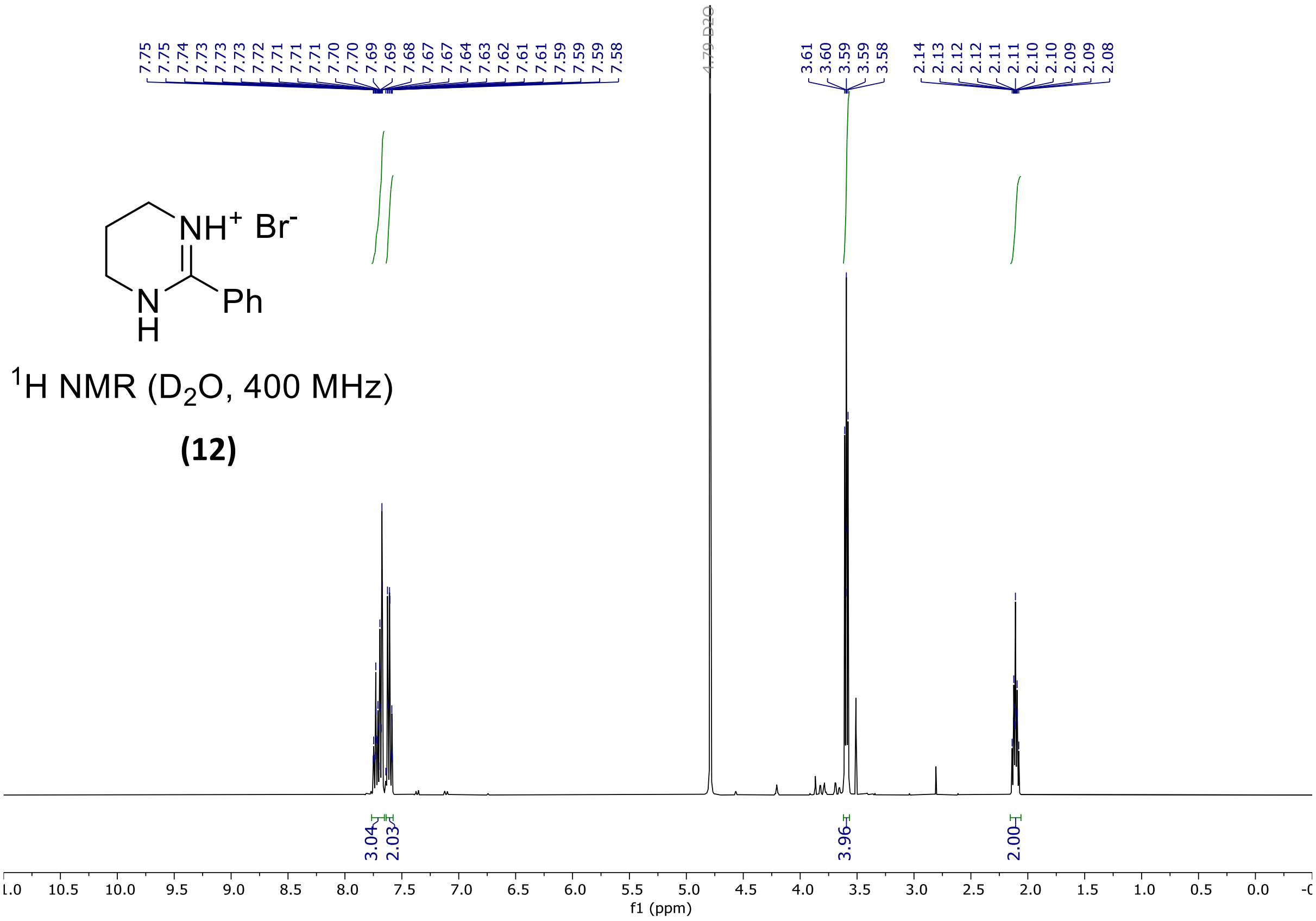

^{1}H NMR (D_2O, 400 MHz)

(12)

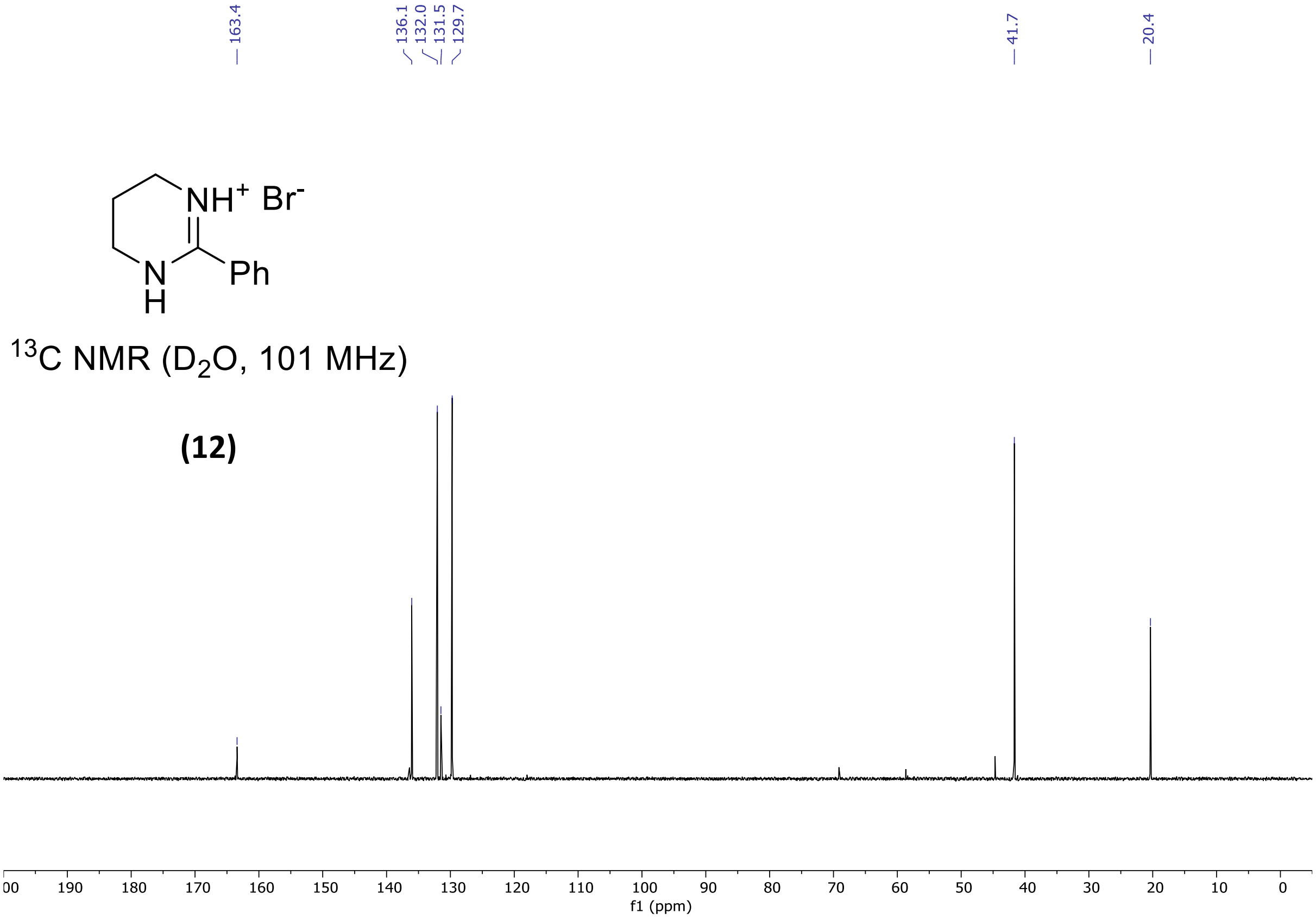
163.4
136.1
132.0
131.5
129.7
41.7
20.4
NH+ Br-
N
H
Ph
13C NMR (D2O, 101 MHz)
(12)
00 190 180 170 160 150 140 130 120 110 100 90 80 70 60 50 40 30 20 10 0
f1 (ppm)

Pyridazines hydrogenation

^{1}H NMR (D_2O, 400 MHz)

(14)

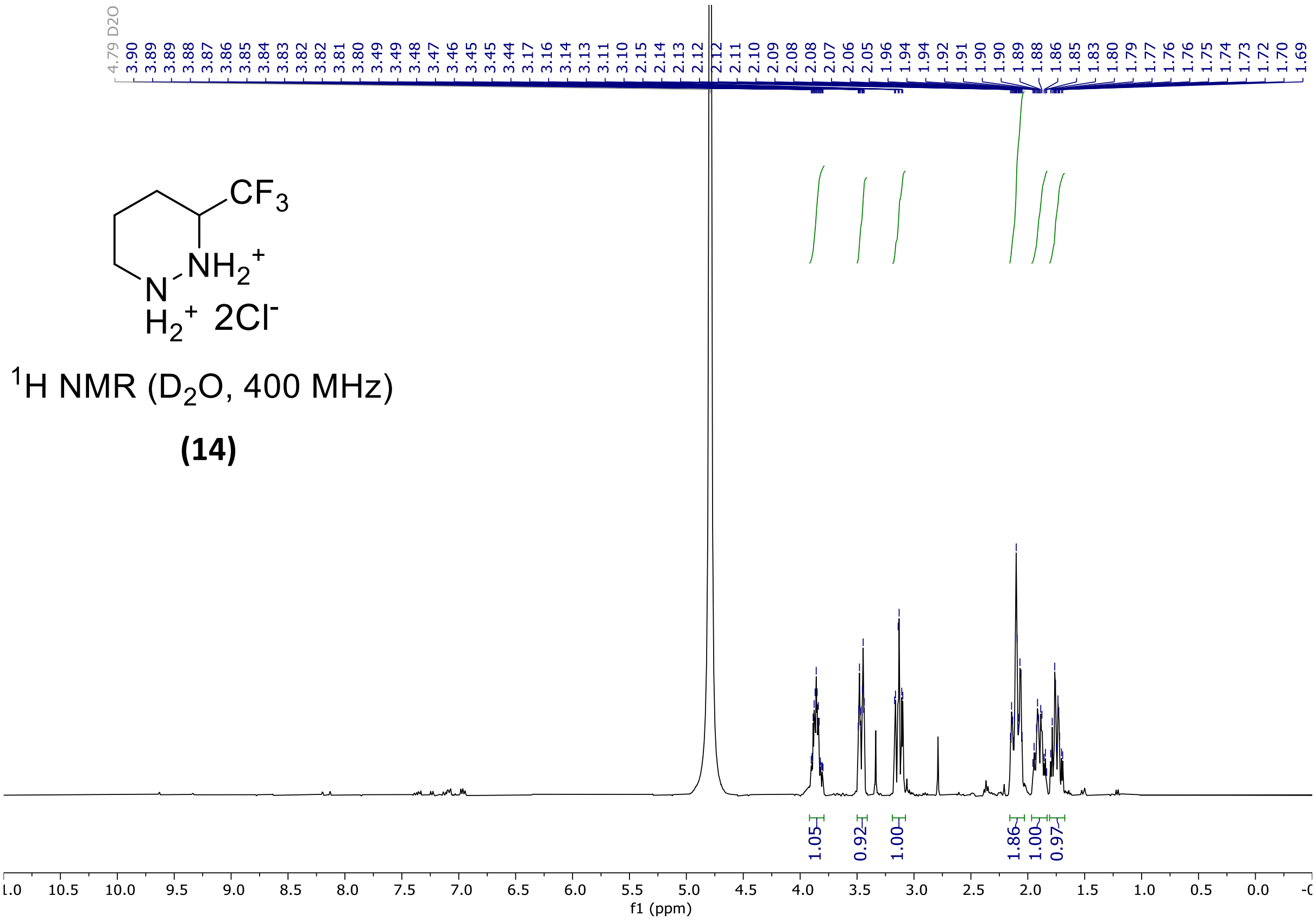

^{13}C NMR (D_2O, 101 MHz)

(14)

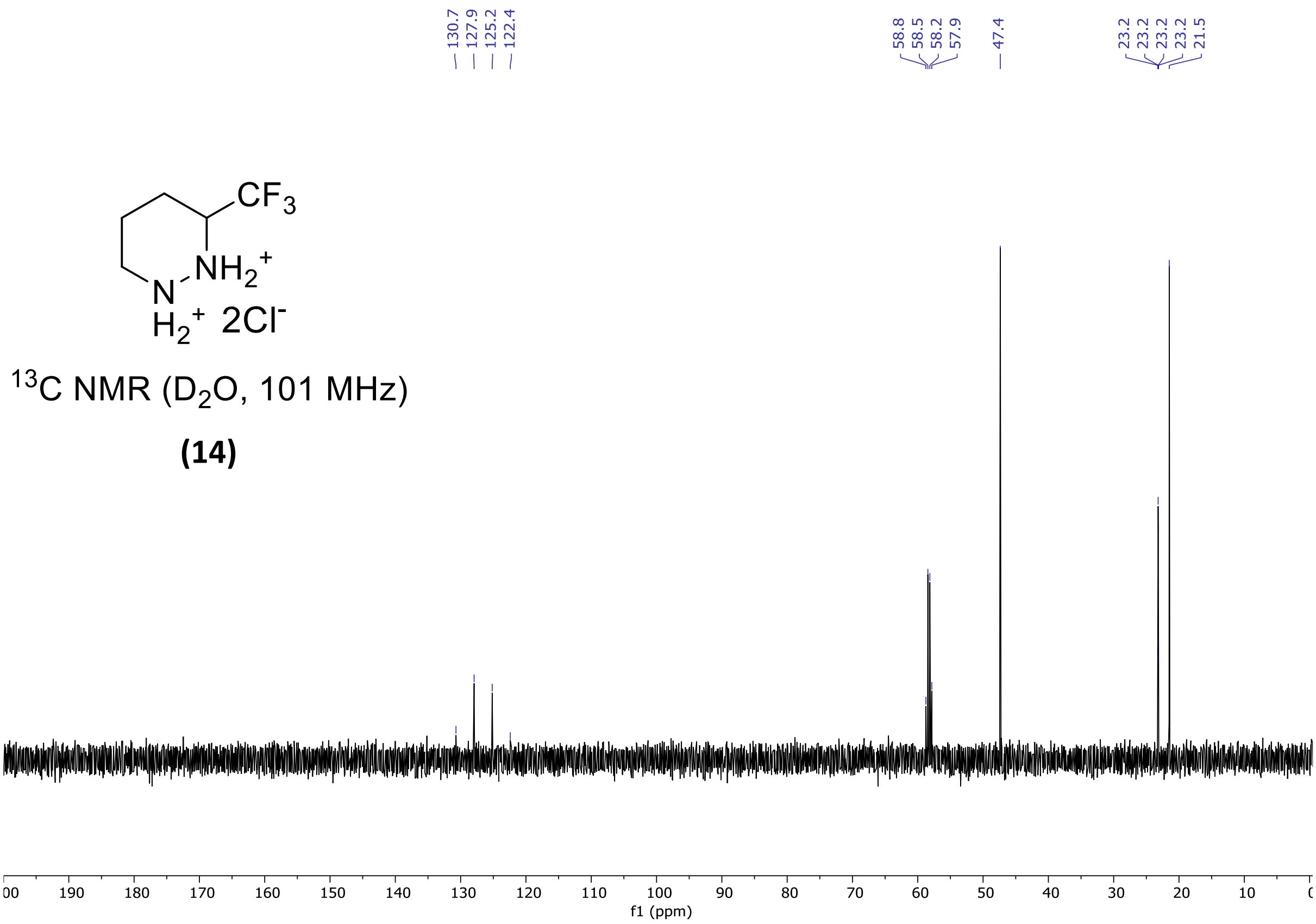

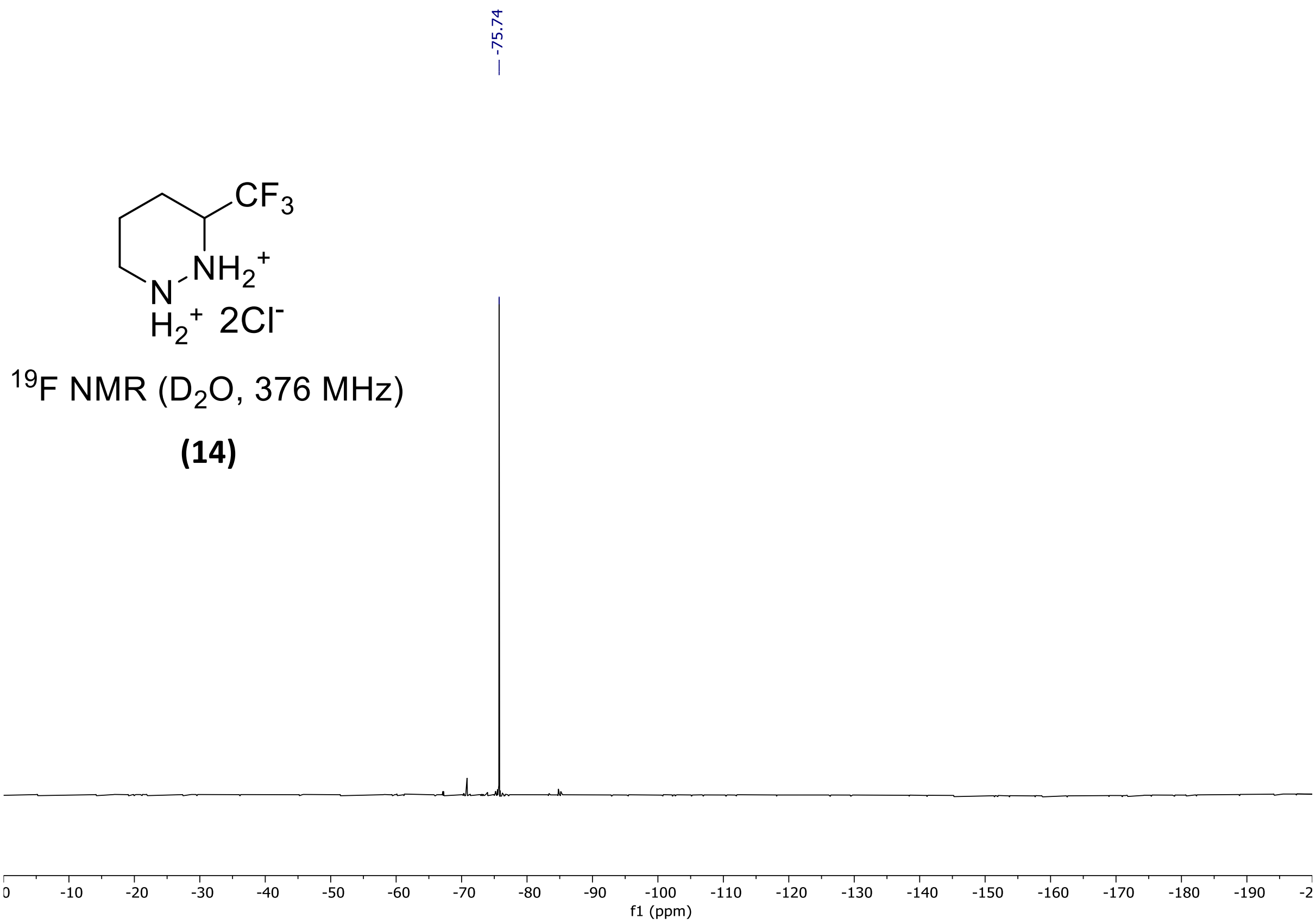

^{19}F NMR (D_2O, 376 MHz)

(14)

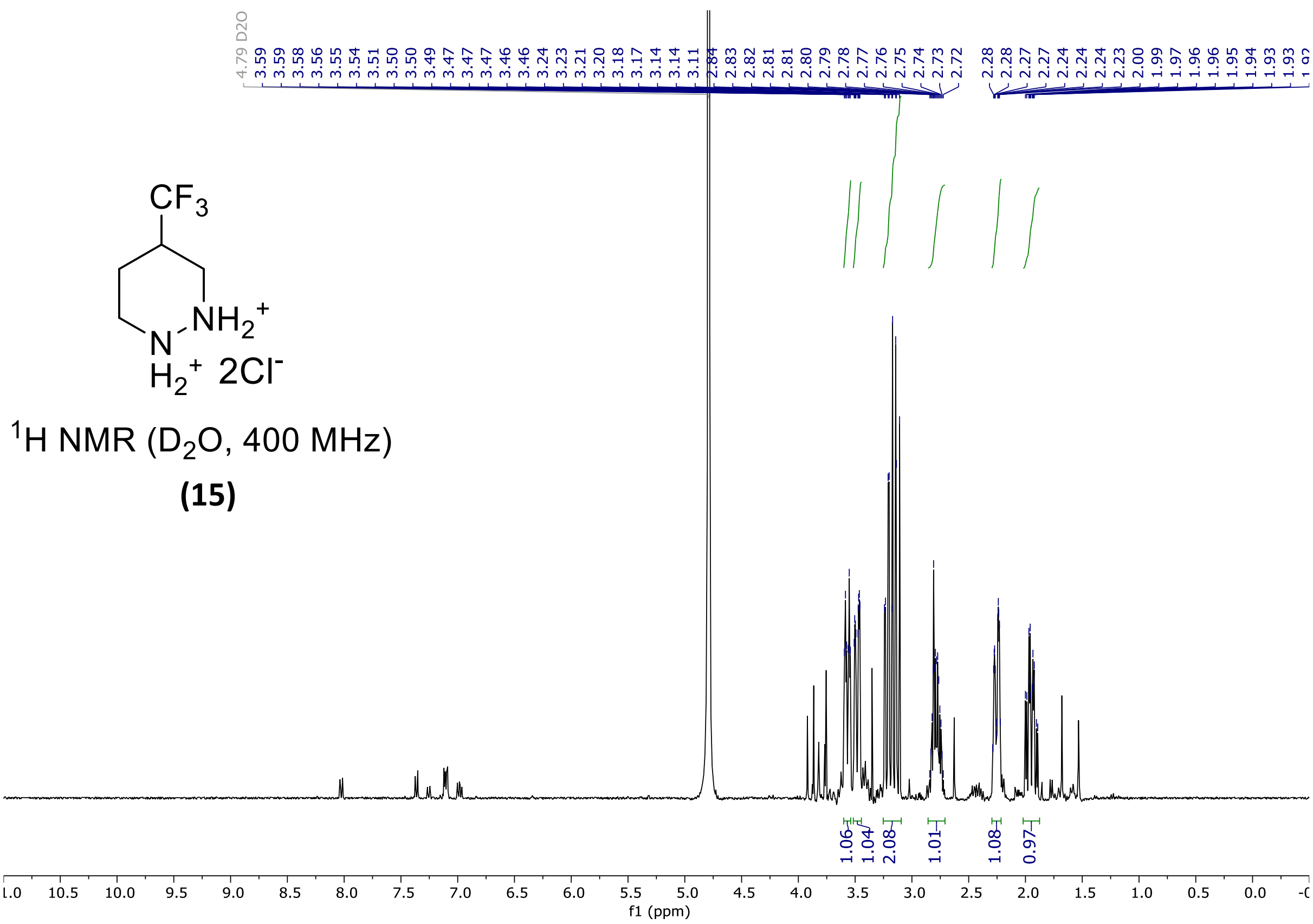

^{1}H NMR (D_2O, 400 MHz)

(15)

^{13}C NMR (D_2O, 101 MHz)

(15)

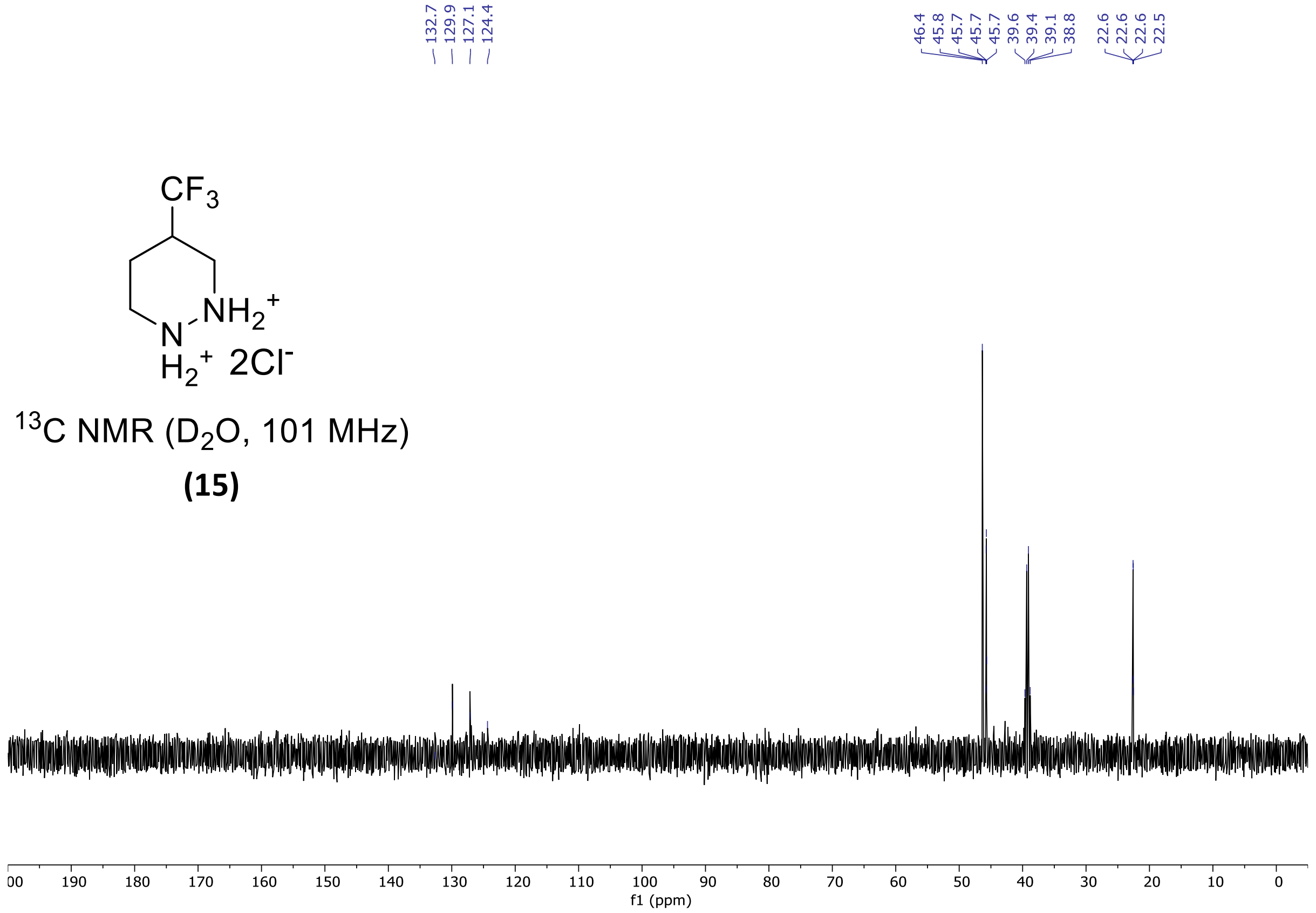

^{19}F NMR (D_2O, 376 MHz)

(15)

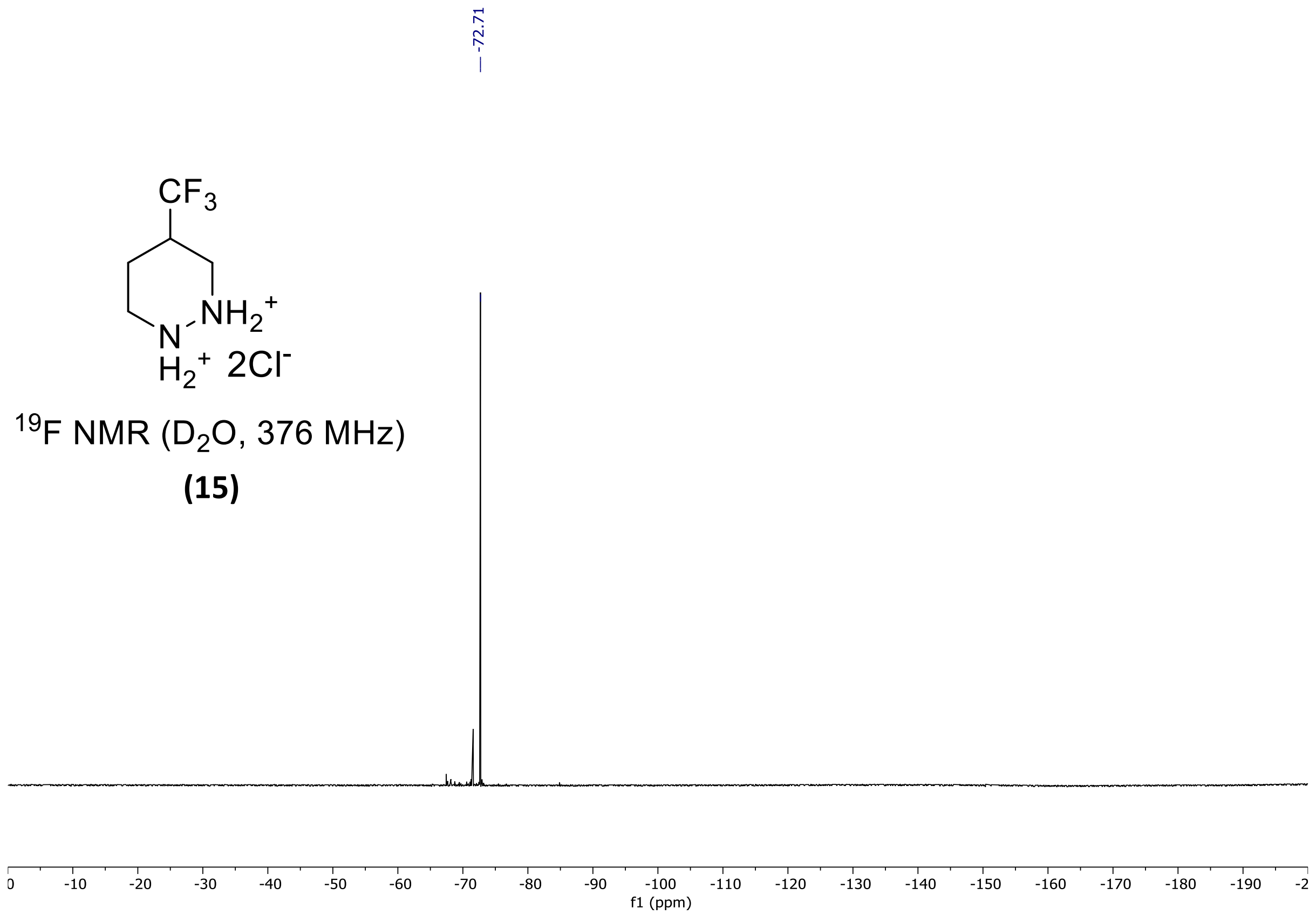

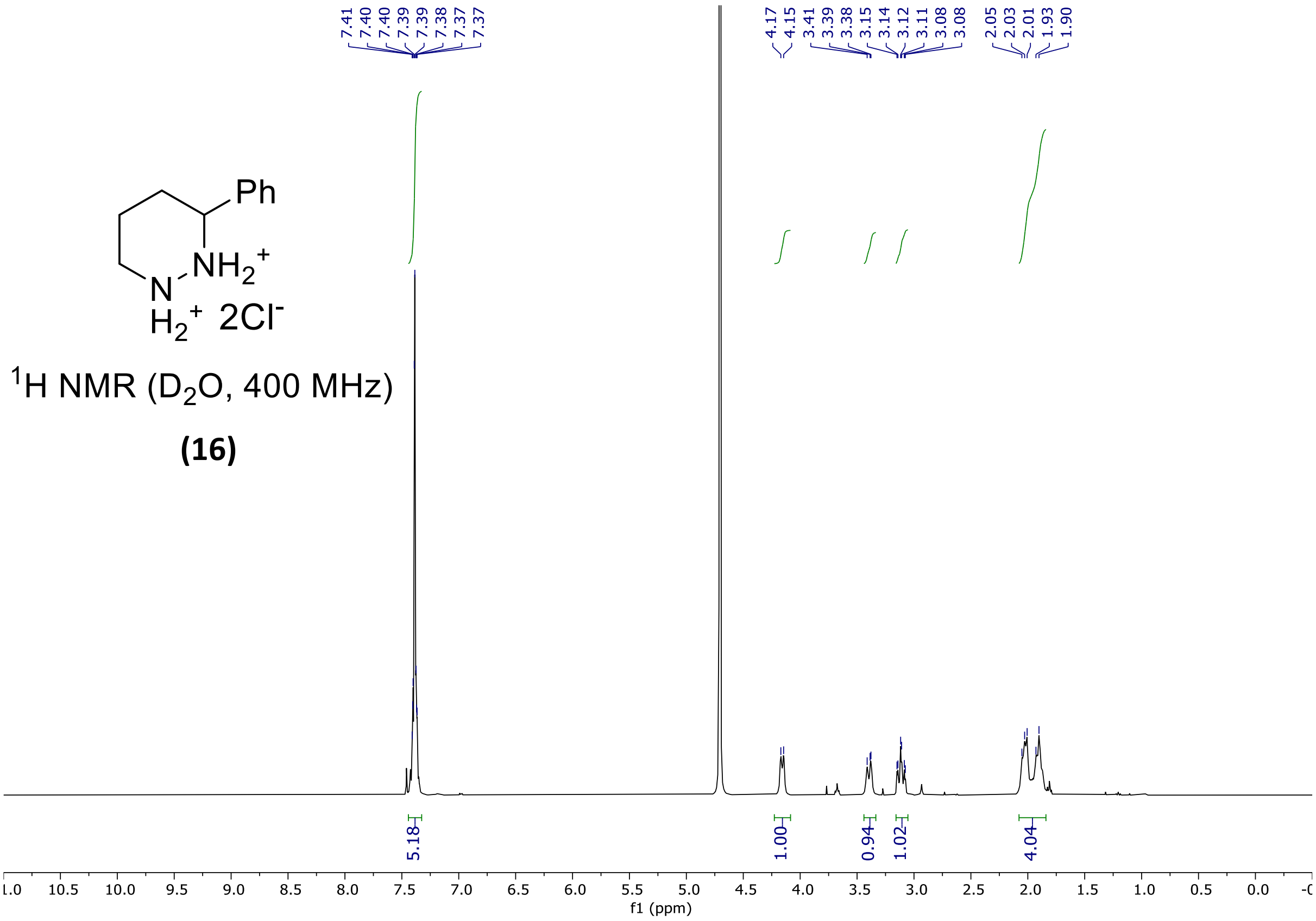

7.41
7.40
7.40
7.39
7.39
7.38
7.37
7.37
4.17
4.15
3.41
3.39
3.38
3.15
3.14
3.12
3.11
3.08
3.08
2.05
2.03
2.01
1.93
1.90
Ph
NH2+
N
H2+ 2Cl-
1H NMR (D2O, 400 MHz)
(16)
5.18
1.00
0.94
1.02
4.04
f1 (ppm)

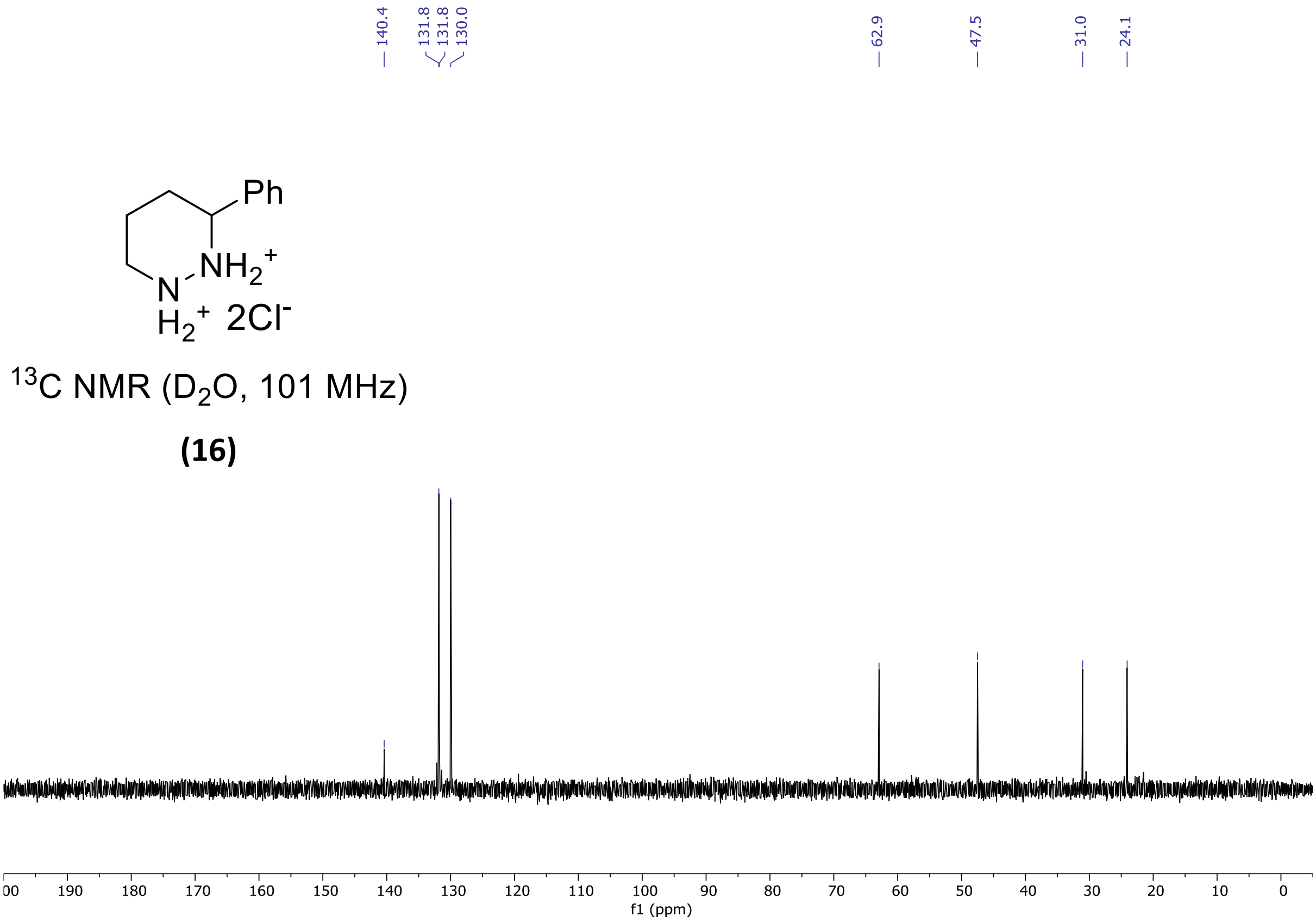

140.4
131.8
131.8
130.0
62.9
47.5
31.0
24.1
Ph
NH2+
N
H2+ 2Cl-
13C NMR (D2O, 101 MHz)
(16)
f1 (ppm)

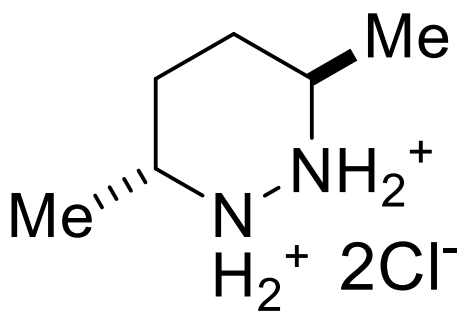

^{1}H NMR (D_2O, 400 MHz)

(17)

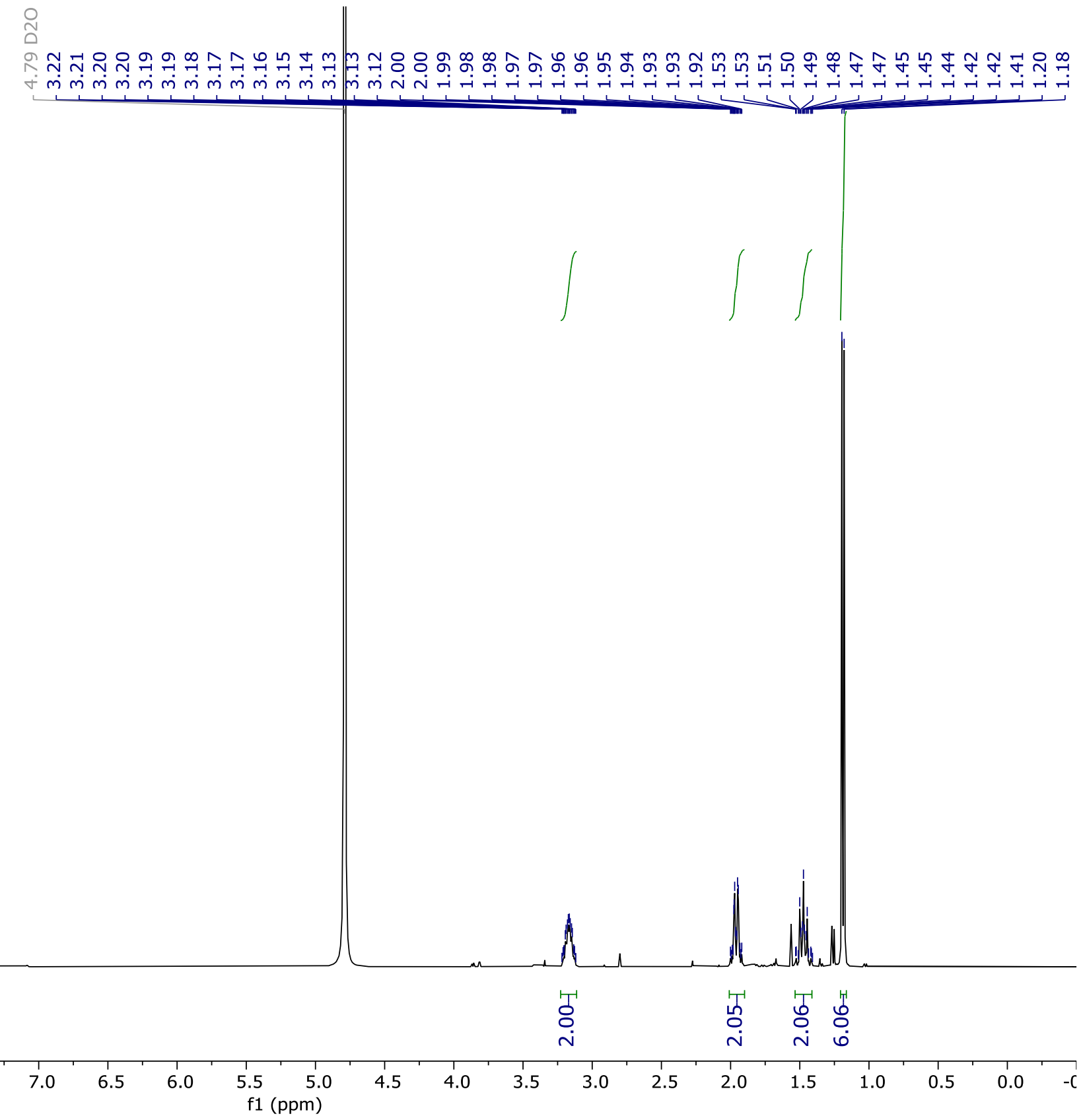

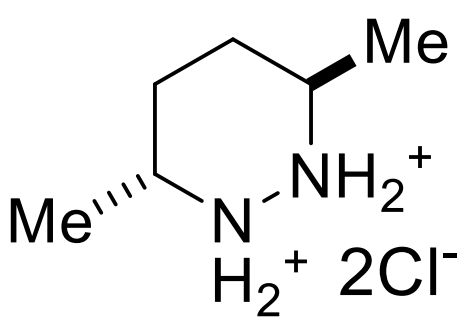

^{13}C NMR (D_2O, 101 MHz)

(17)

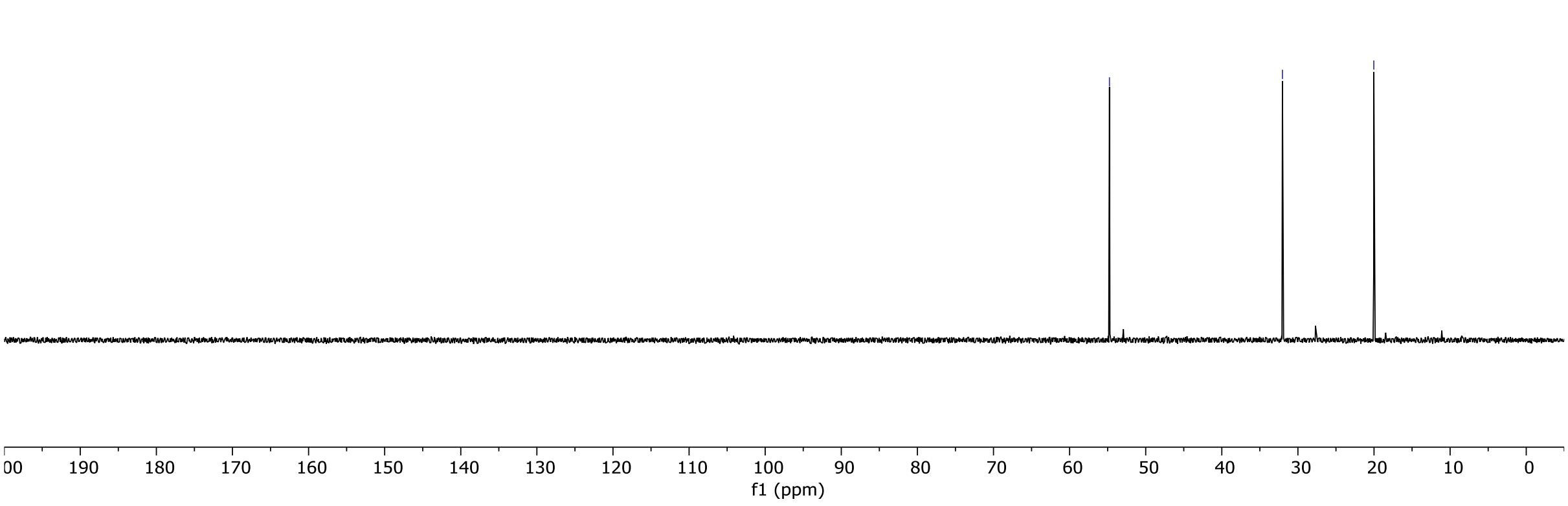

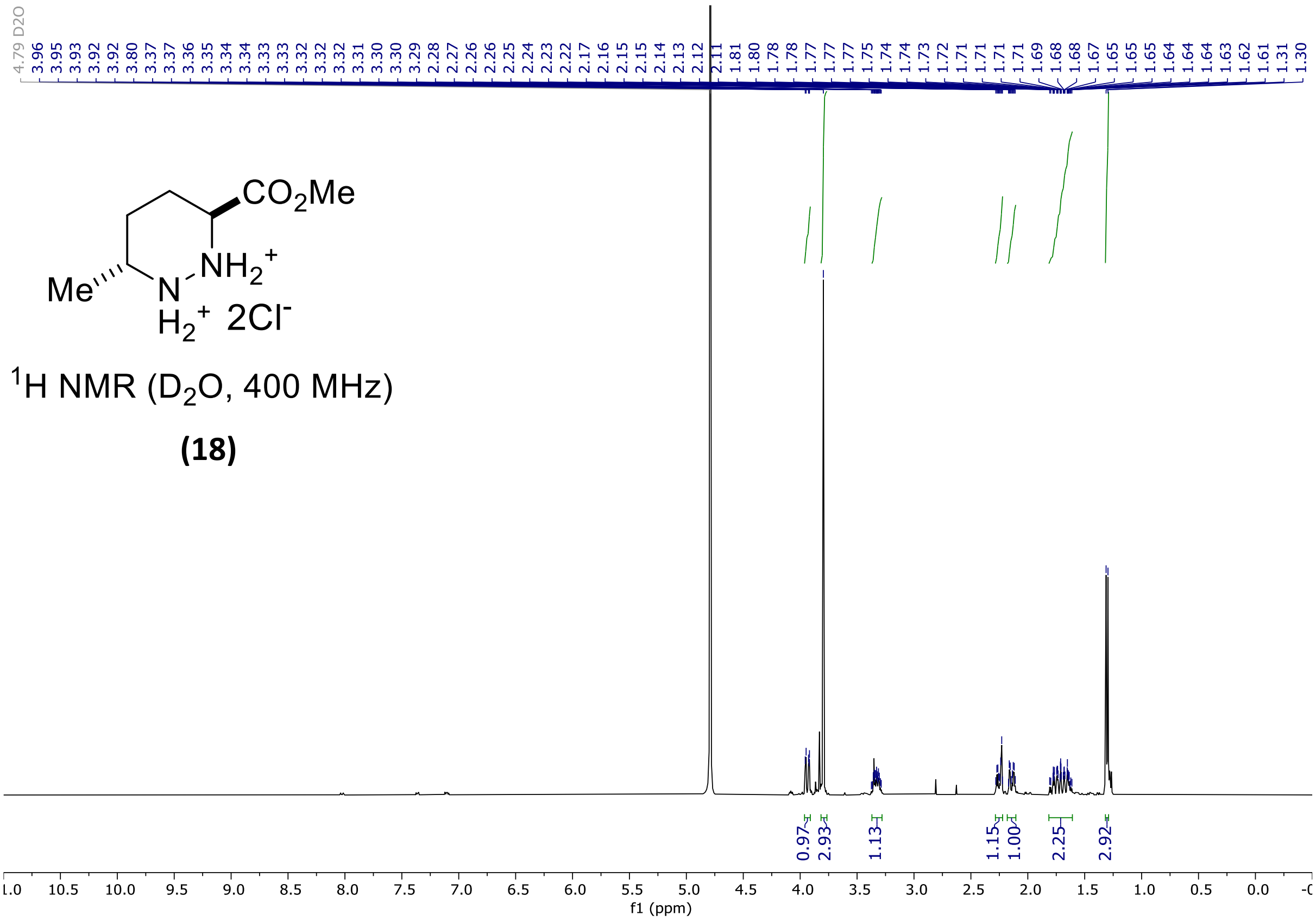

^{1}H NMR (D_2O, 400 MHz)

(18)

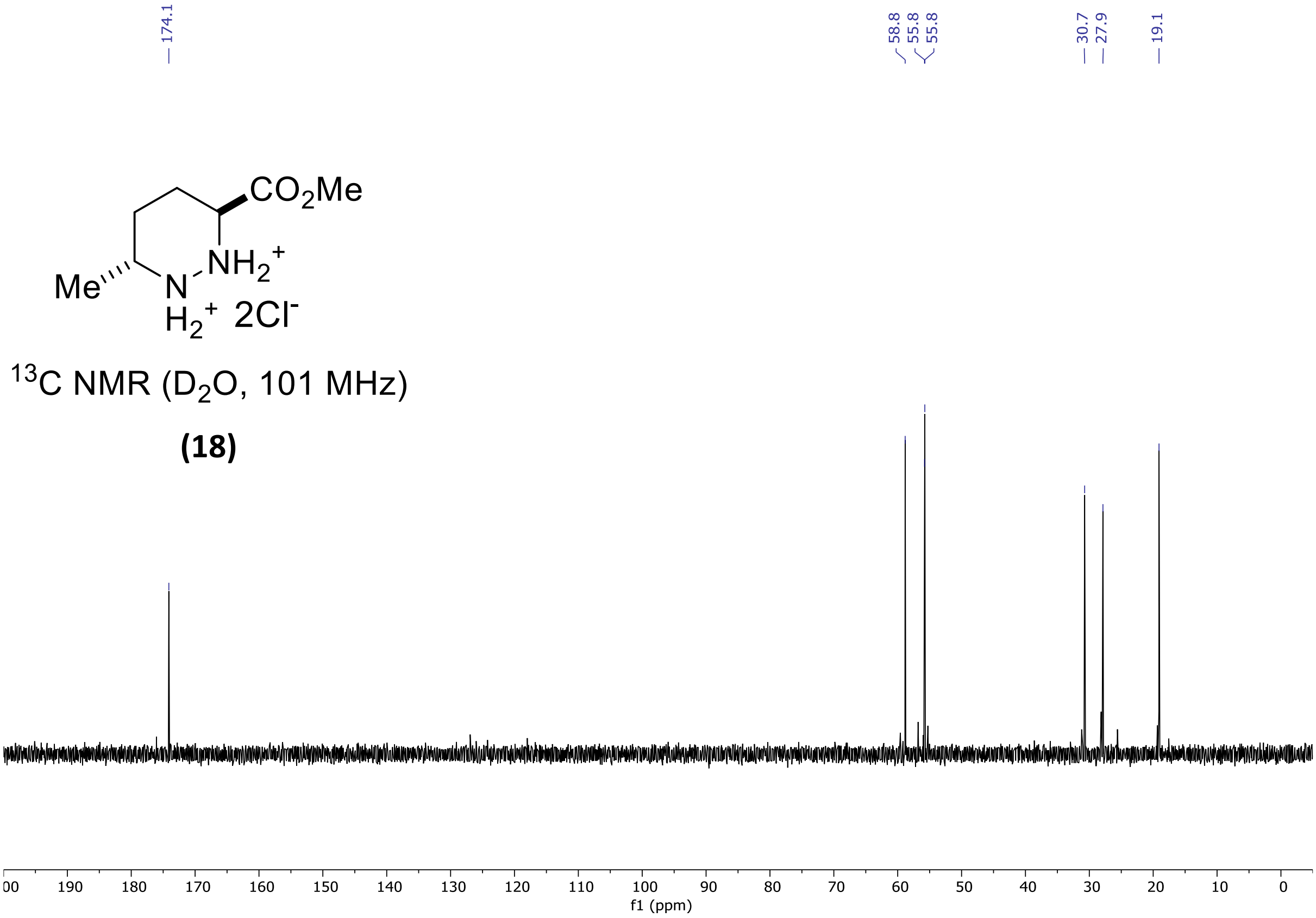

^{13}C NMR (D_2O, 101 MHz)

(18)

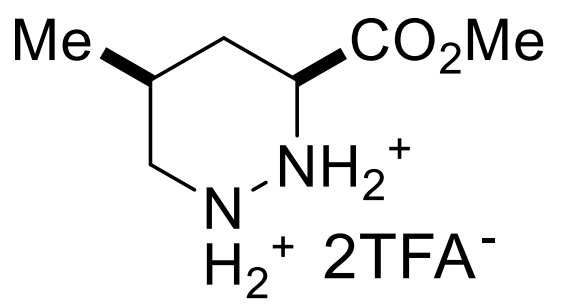

^{1}H NMR (D_2O, 400 MHz)

(19)

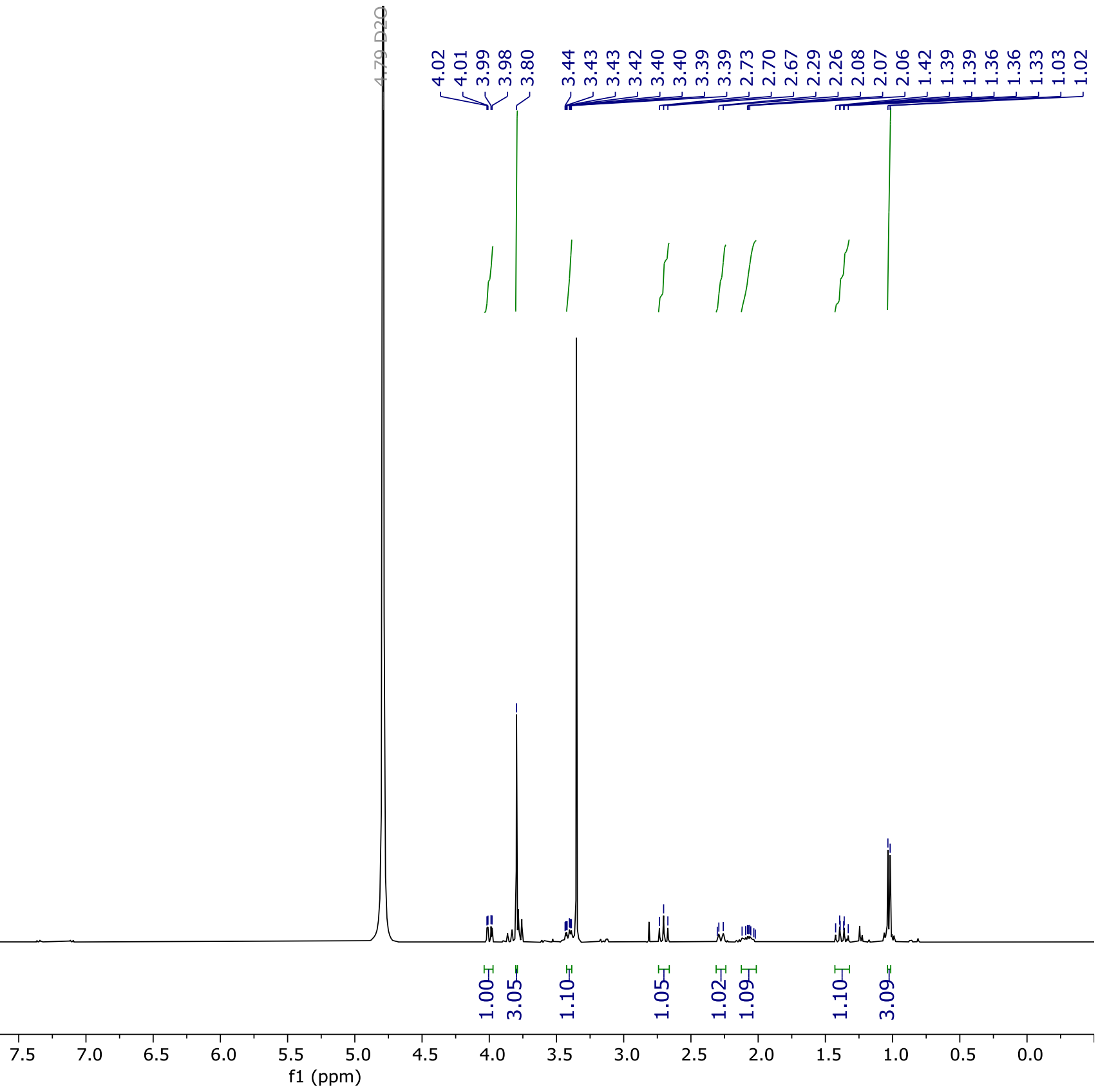

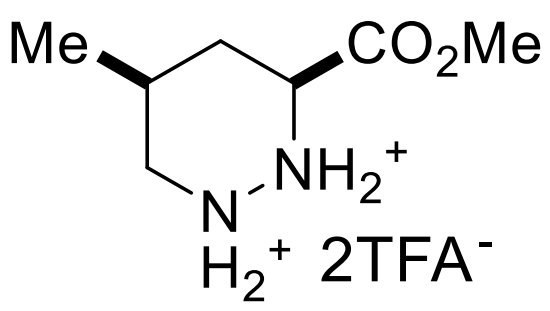

^{13}C NMR (D_2O, 101 MHz)

(19)

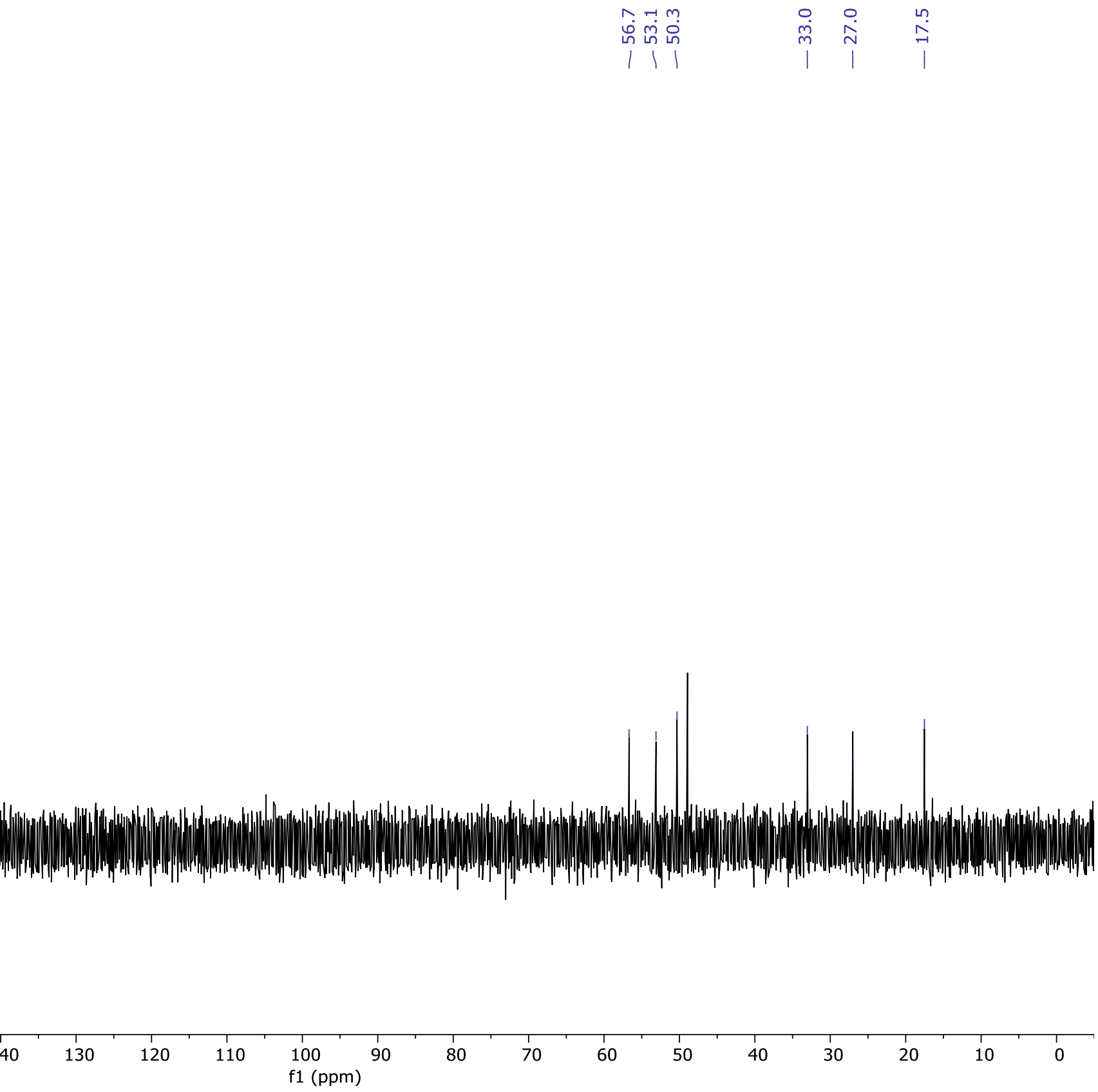

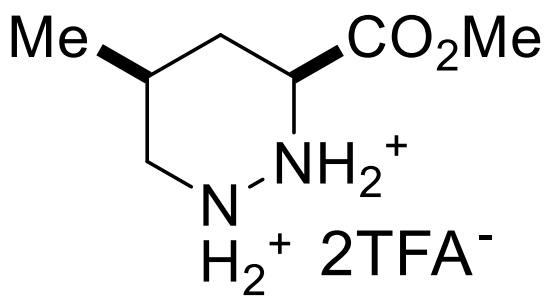

^{13}C NMR (D_2O, 101 MHz)

(19)

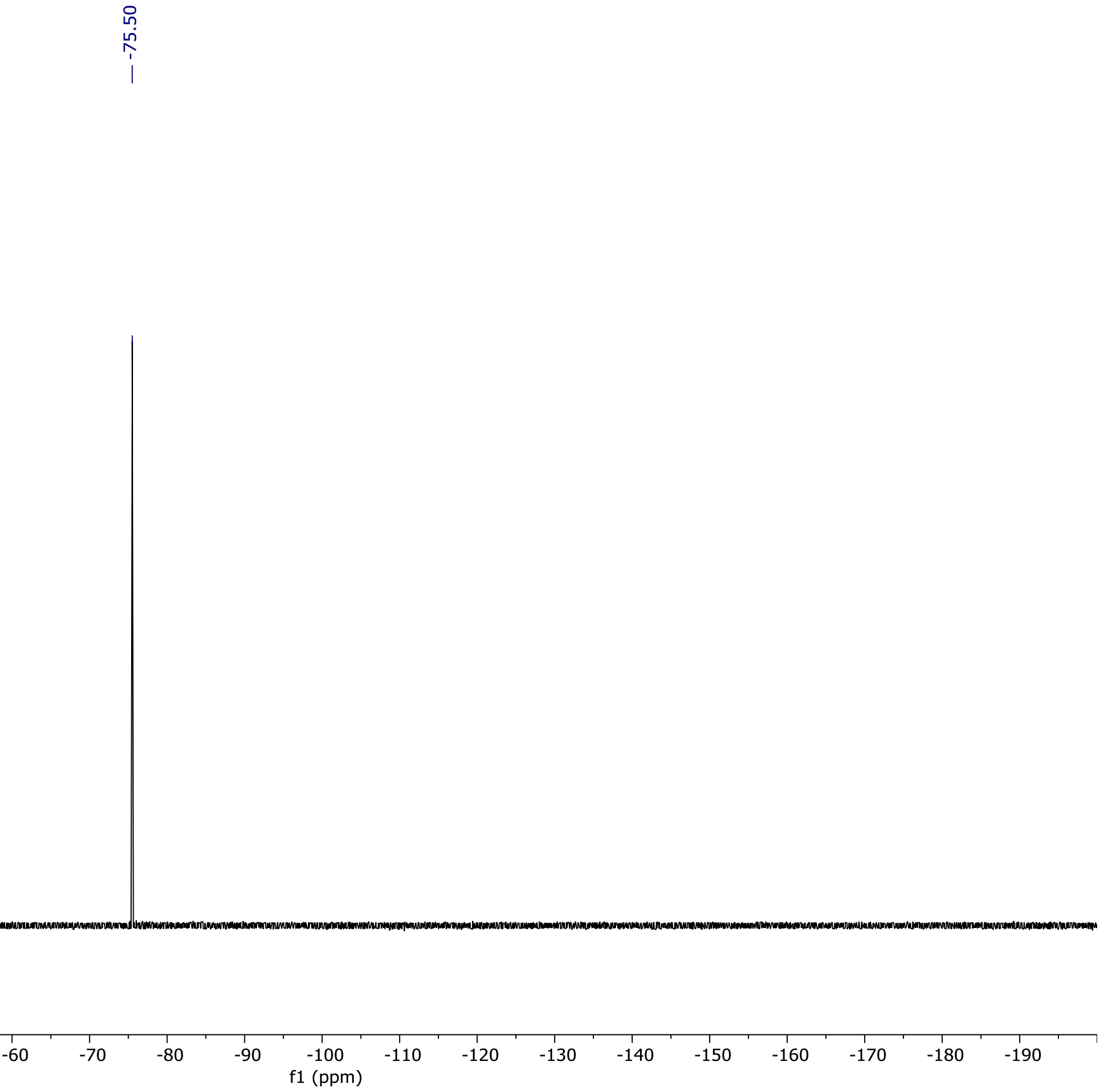

[6,6]-fused heteroarenes hydrogenation

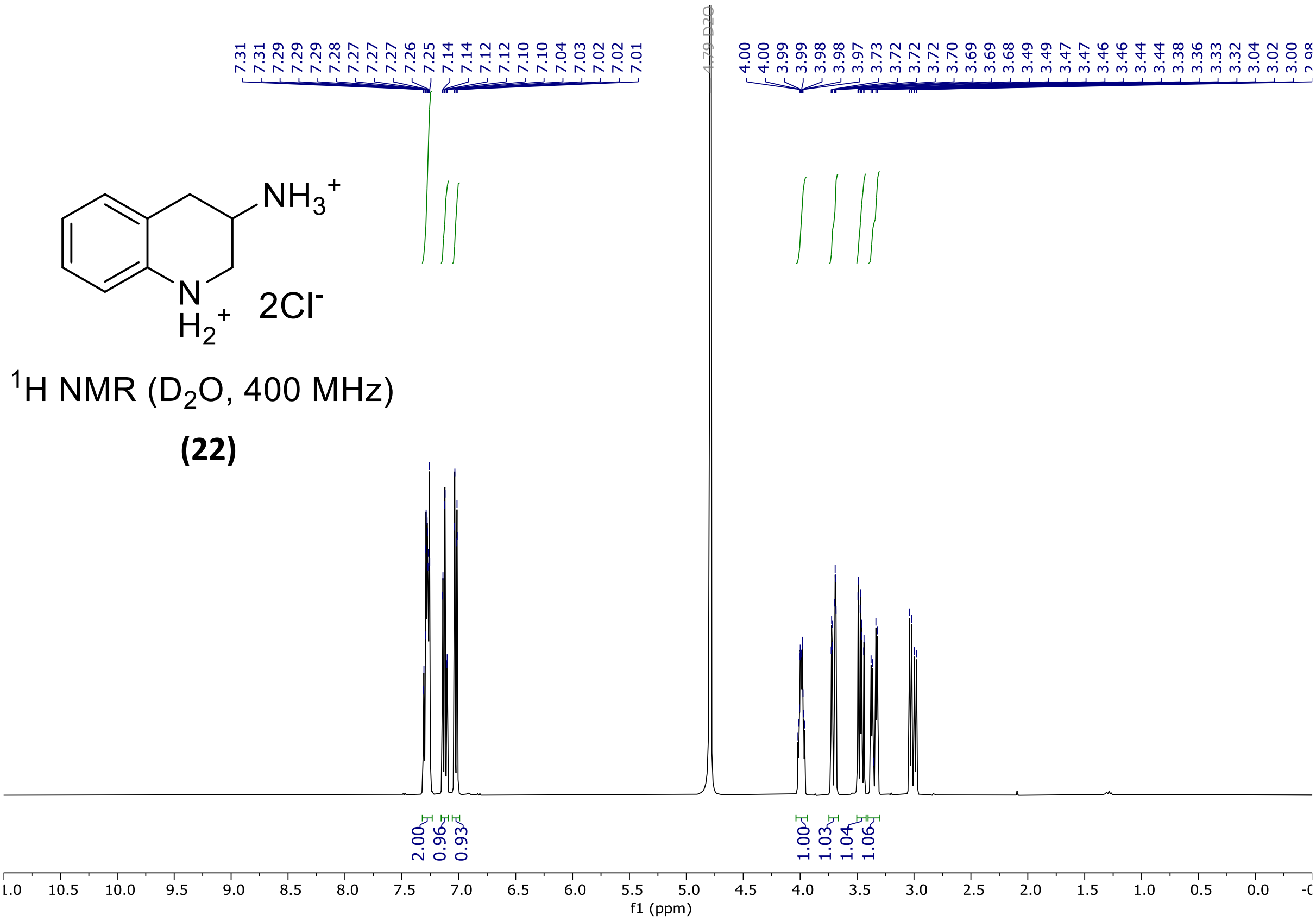

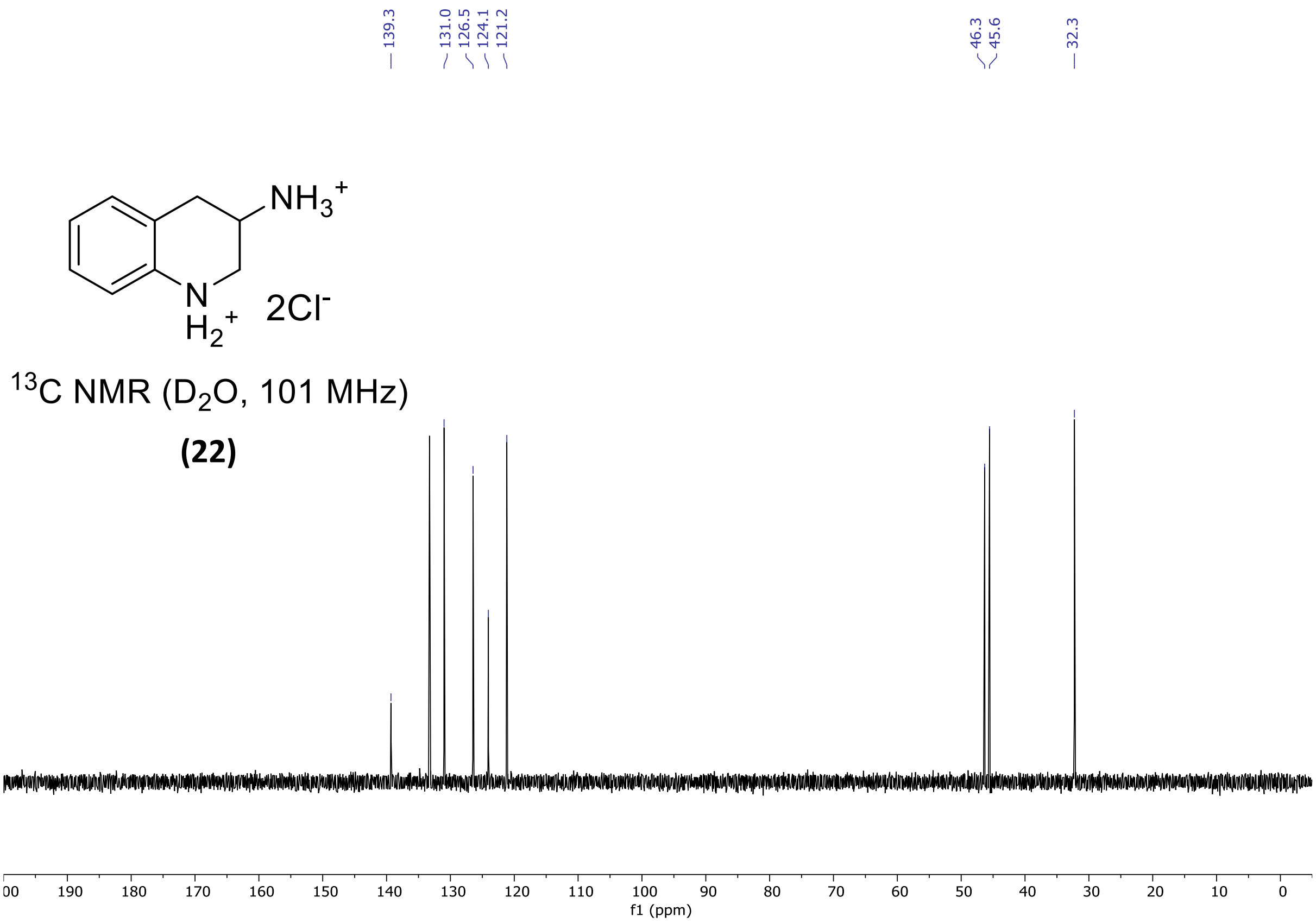

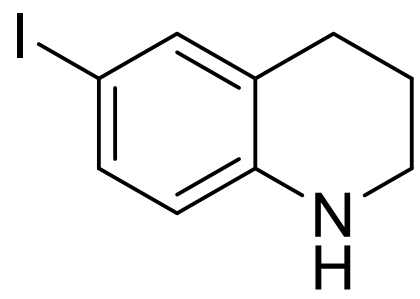

^{1}H NMR ($CDCl_3$, 400 MHz)

(25)

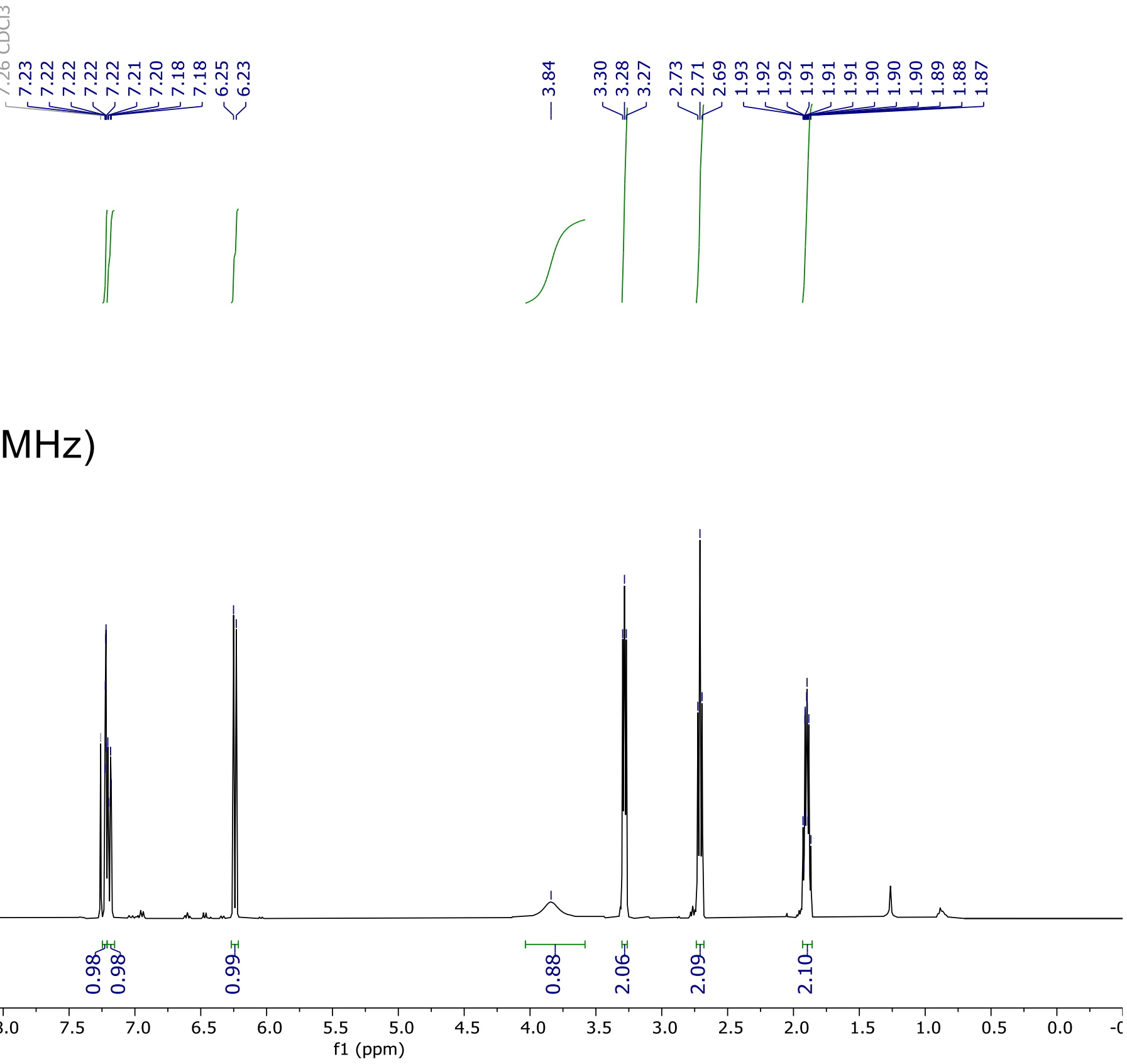

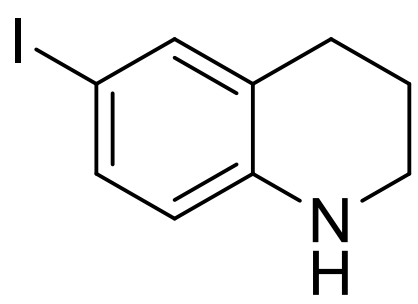

^{13}C NMR ($CDCl_3$, 101 MHz)

(25)

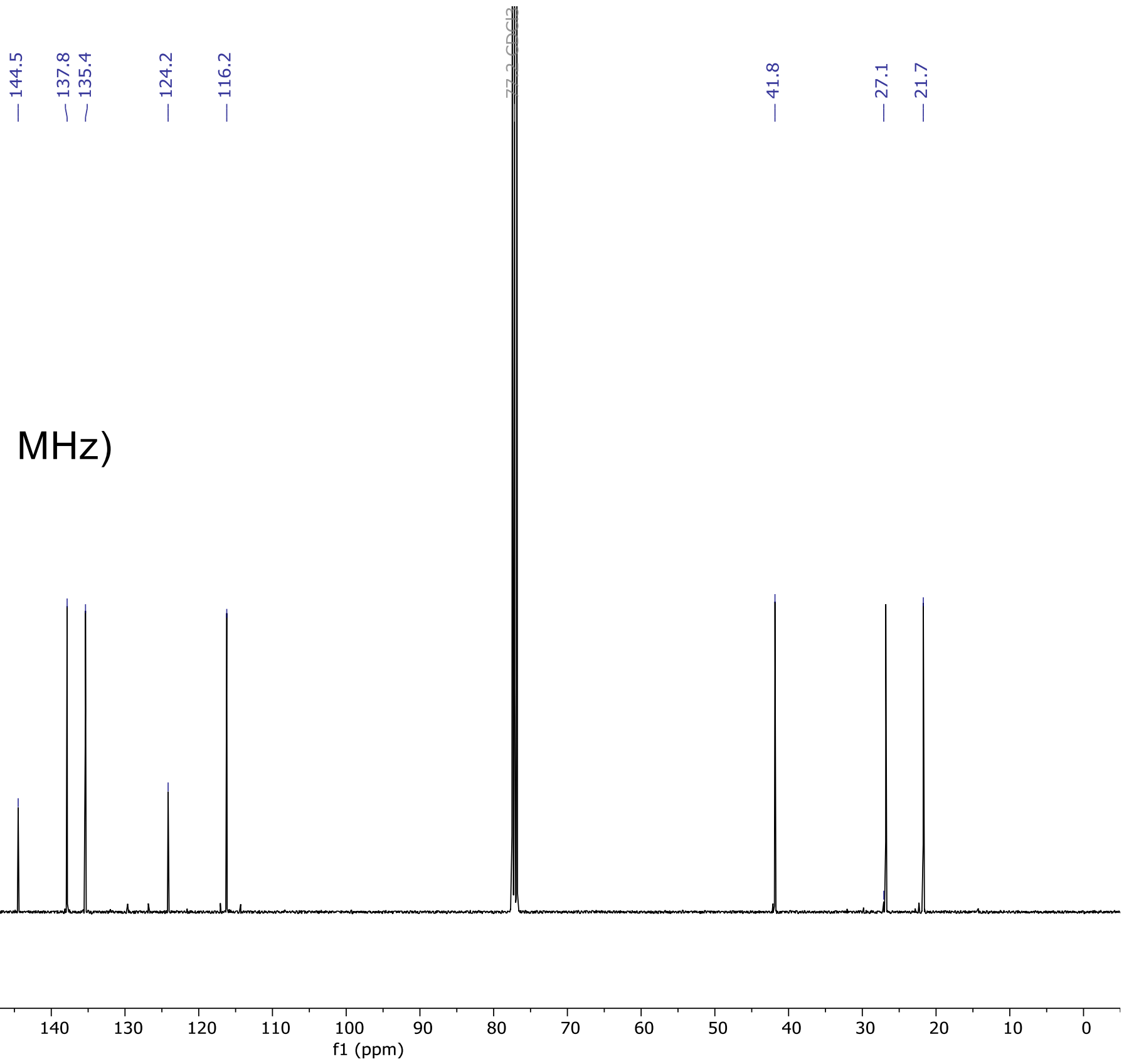

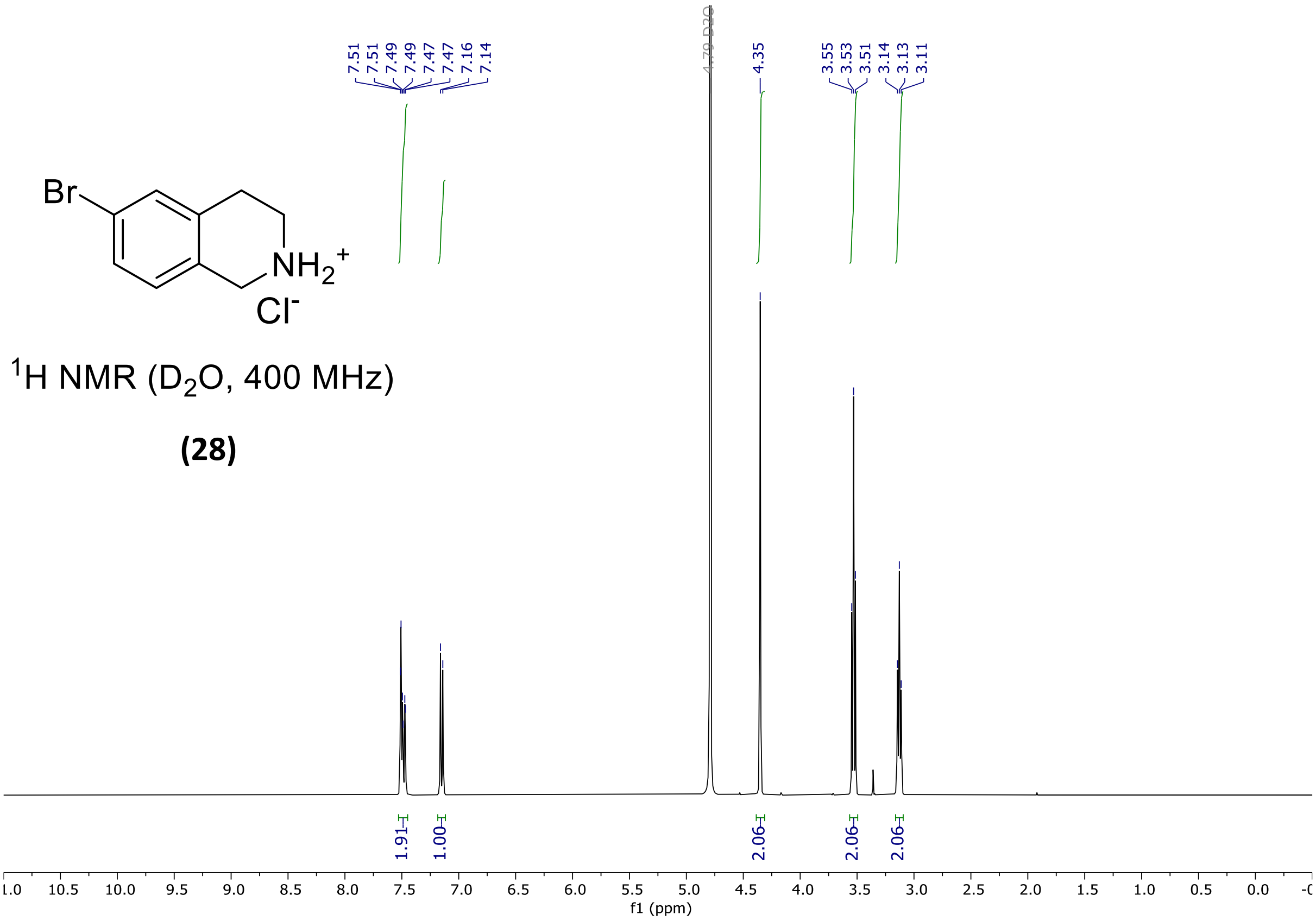

^1H NMR (D_2O, 400 MHz)

(28)

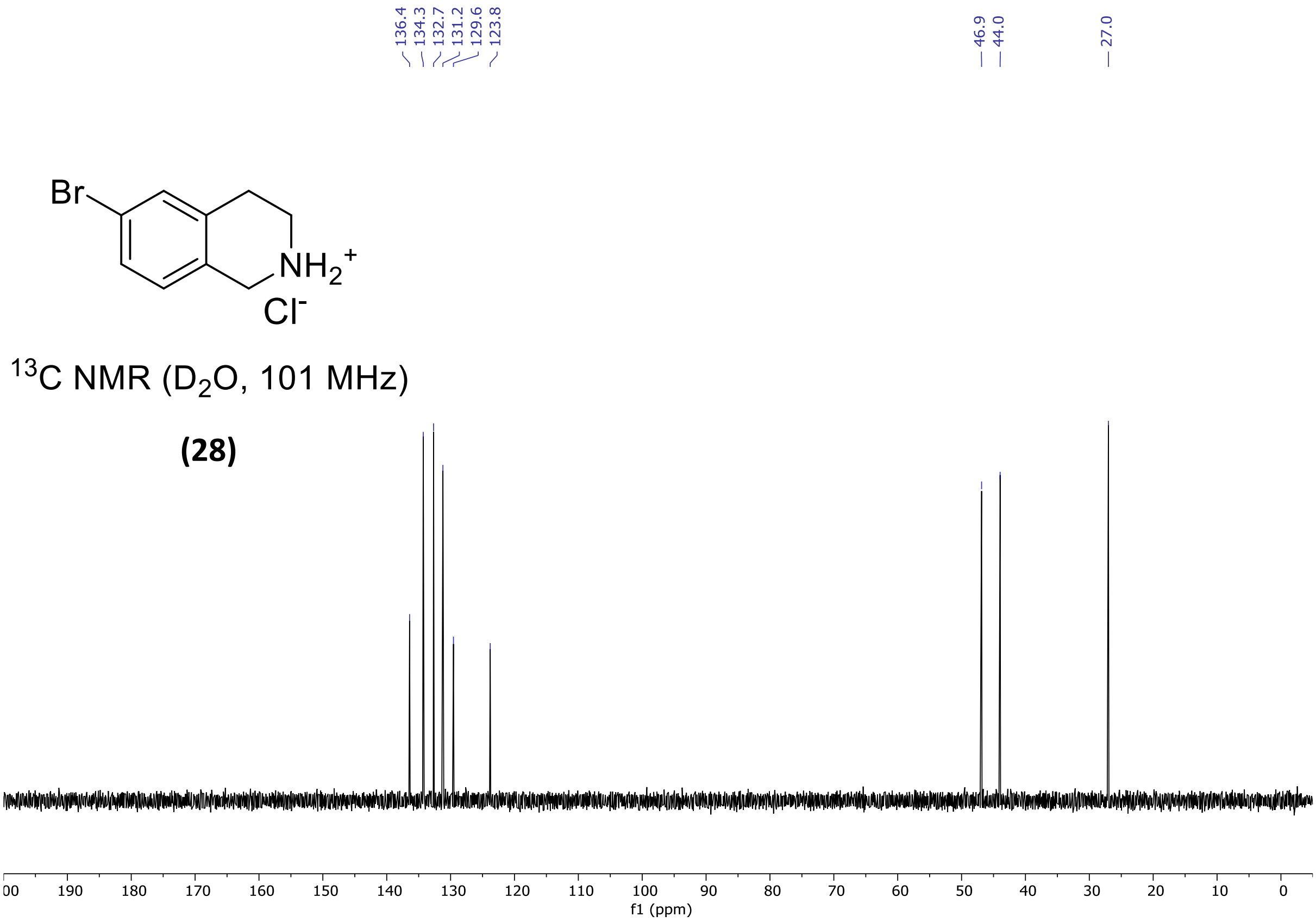

^{13}C NMR (D_2O, 101 MHz)

(28)

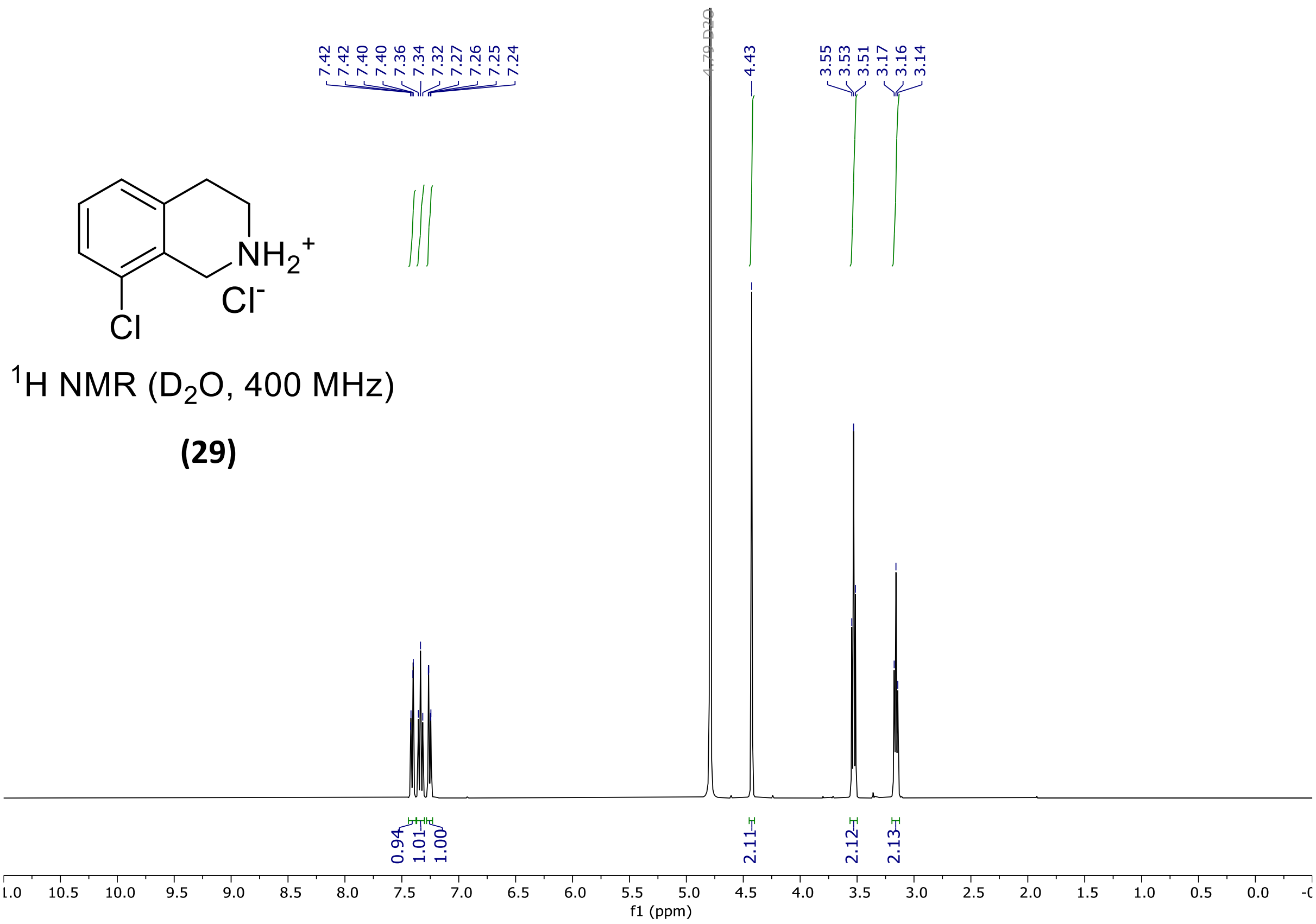

^{1}H NMR (D_2O, 400 MHz)

(29)

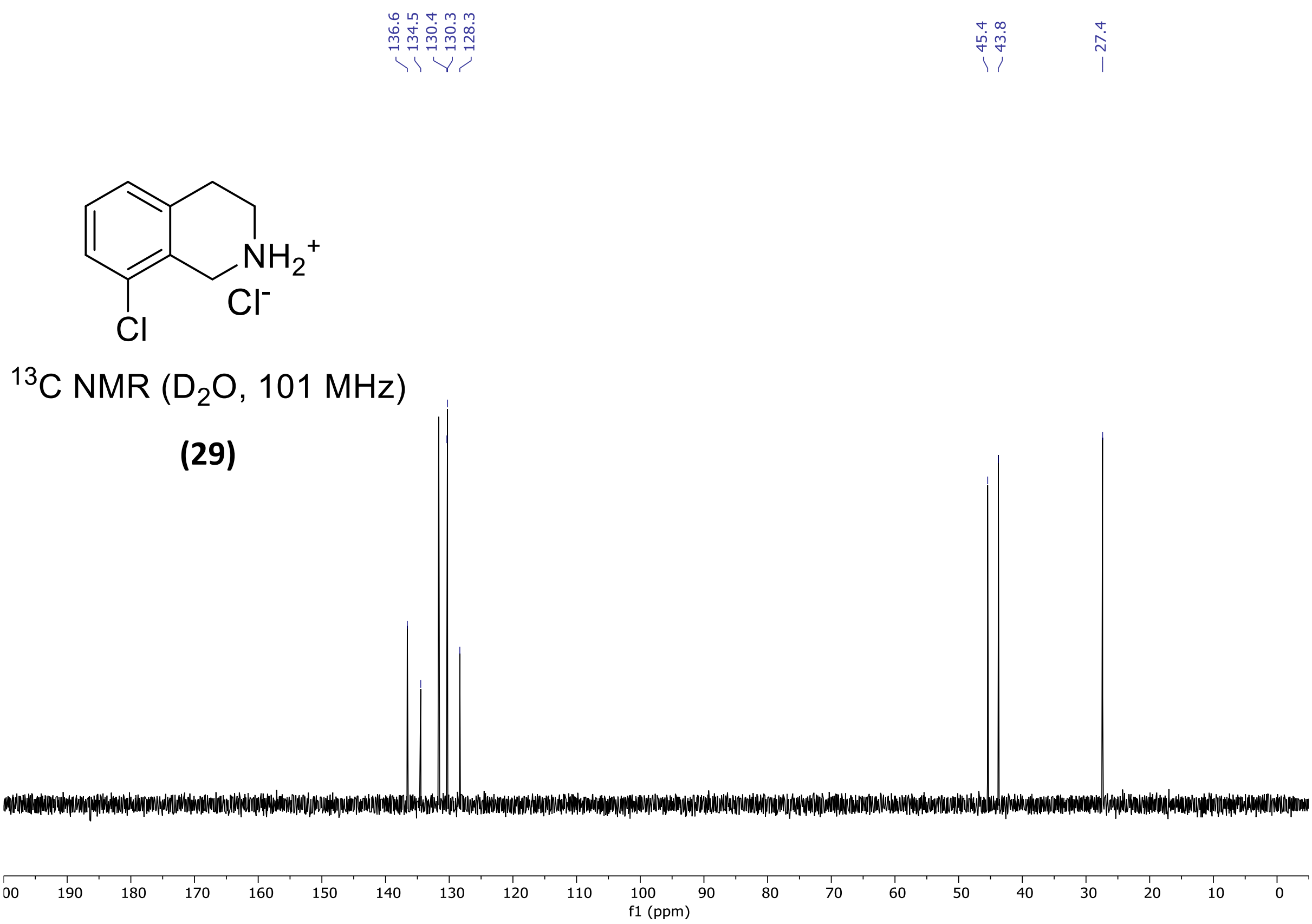

^{13}C NMR (D_2O, 101 MHz)

(29)

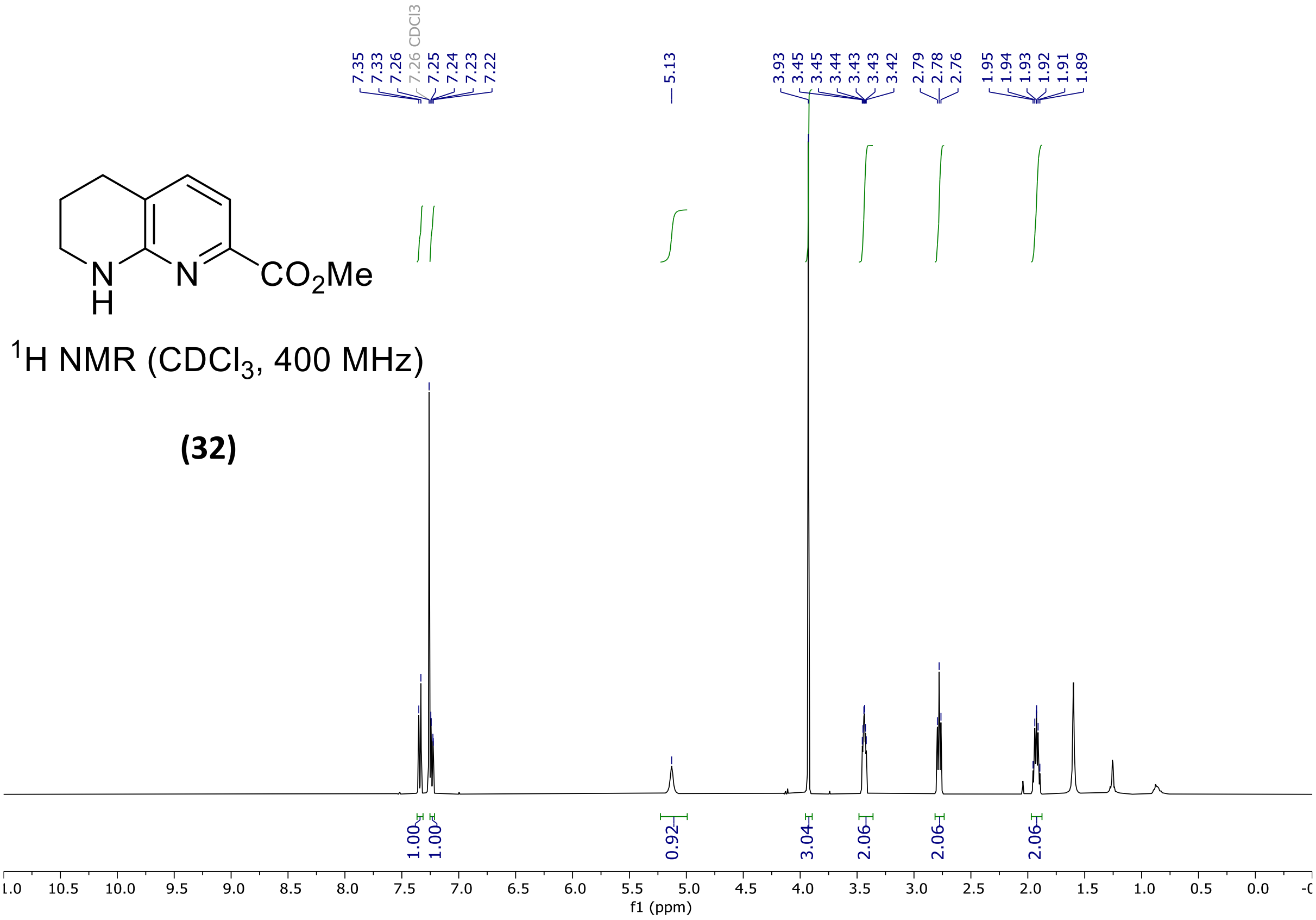

^{1}H NMR ($CDCl_3$, 400 MHz)

(32)

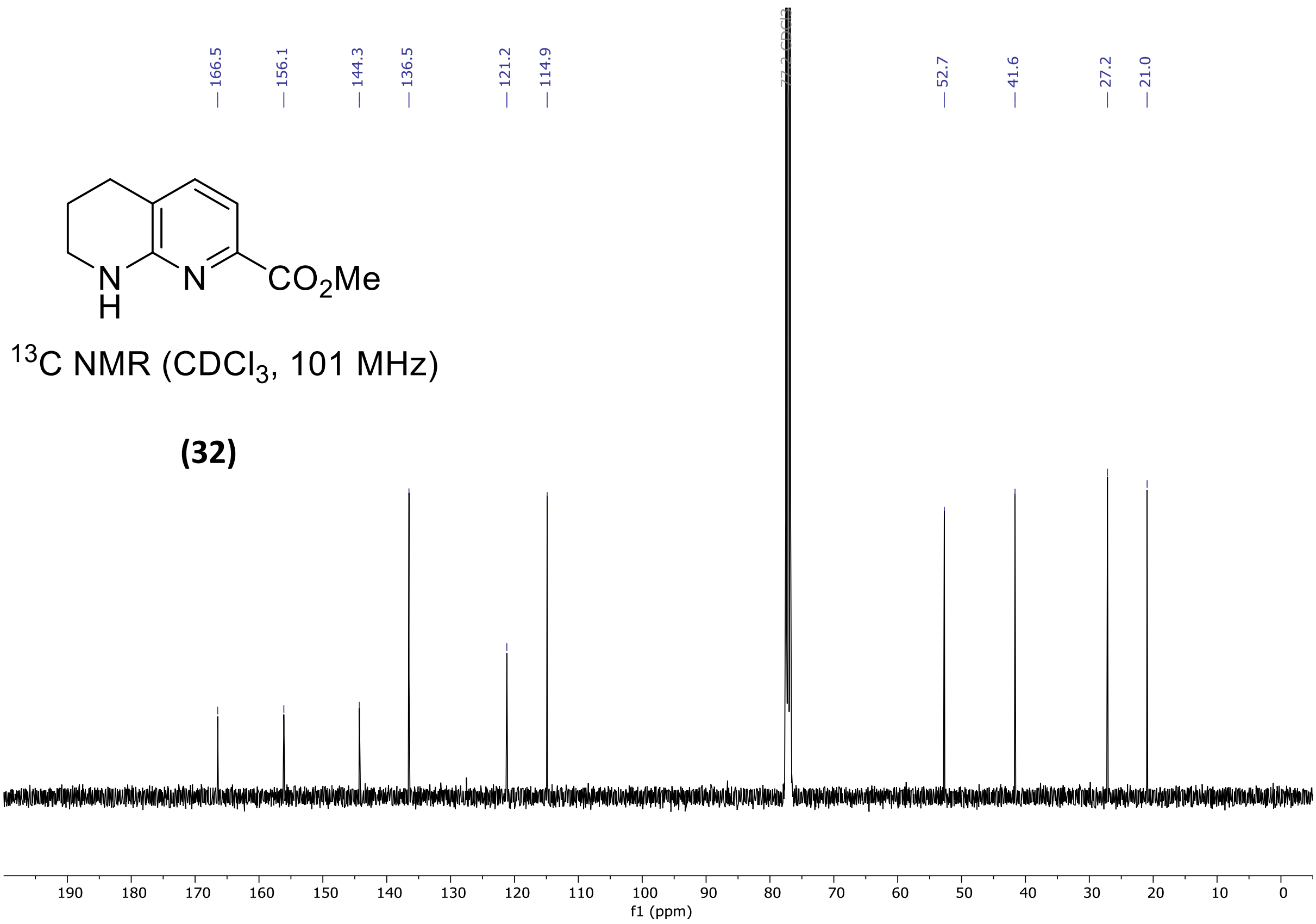

^{13}C NMR ($CDCl_3$, 101 MHz)

(32)

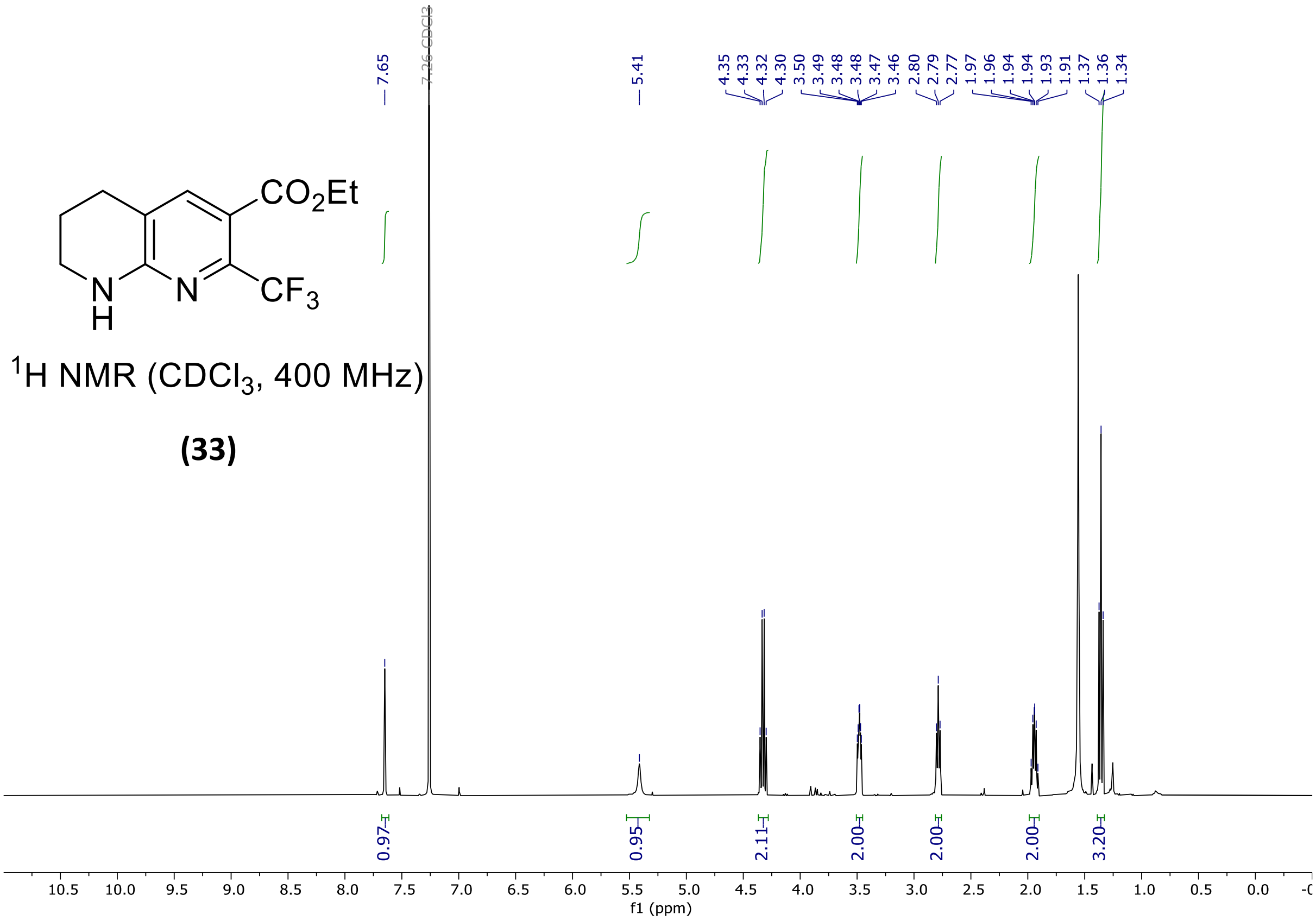

^{1}H NMR ($CDCl_3$, 400 MHz)

(33)

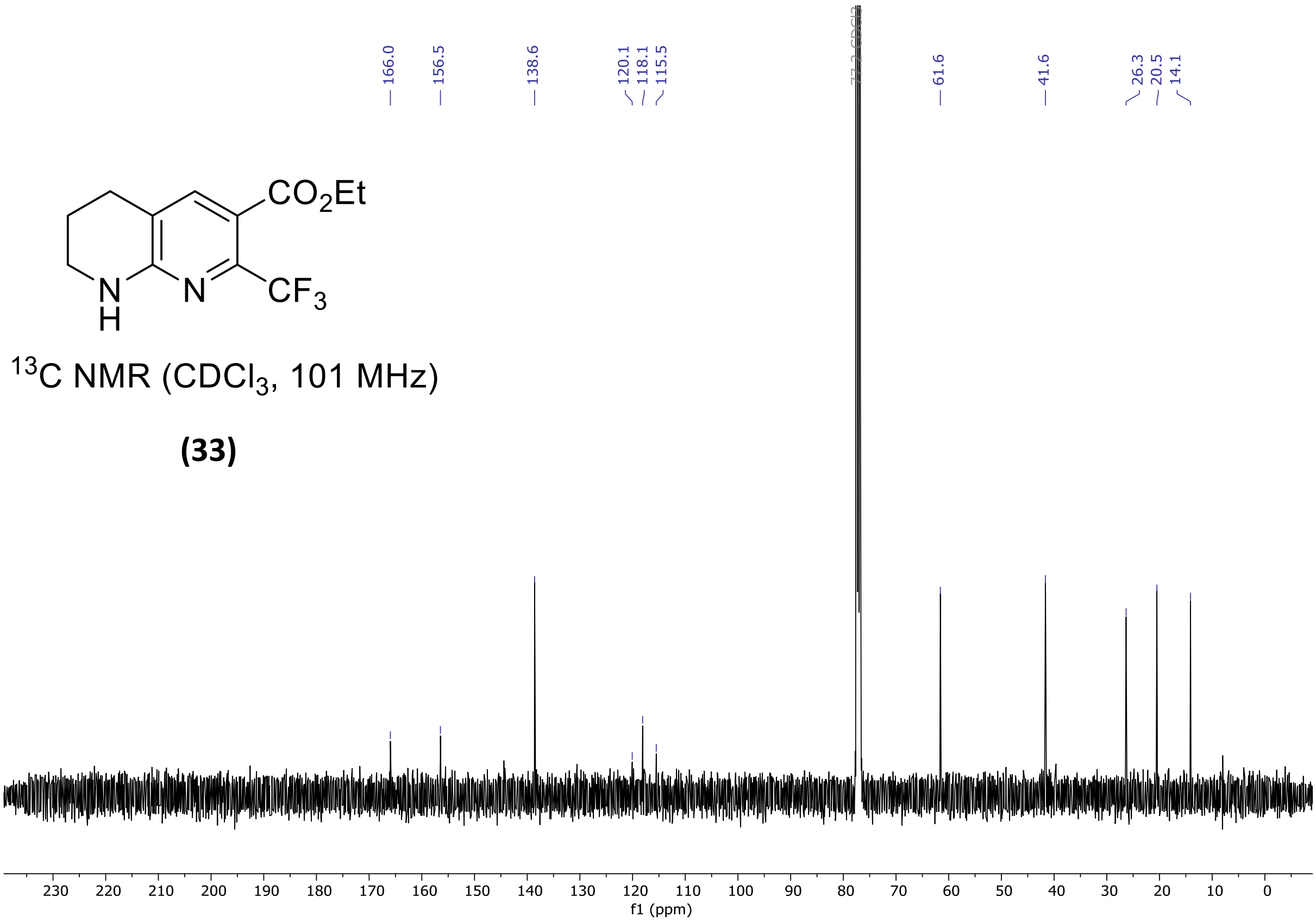

^{13}C NMR ($CDCl_3$, 101 MHz)

(33)

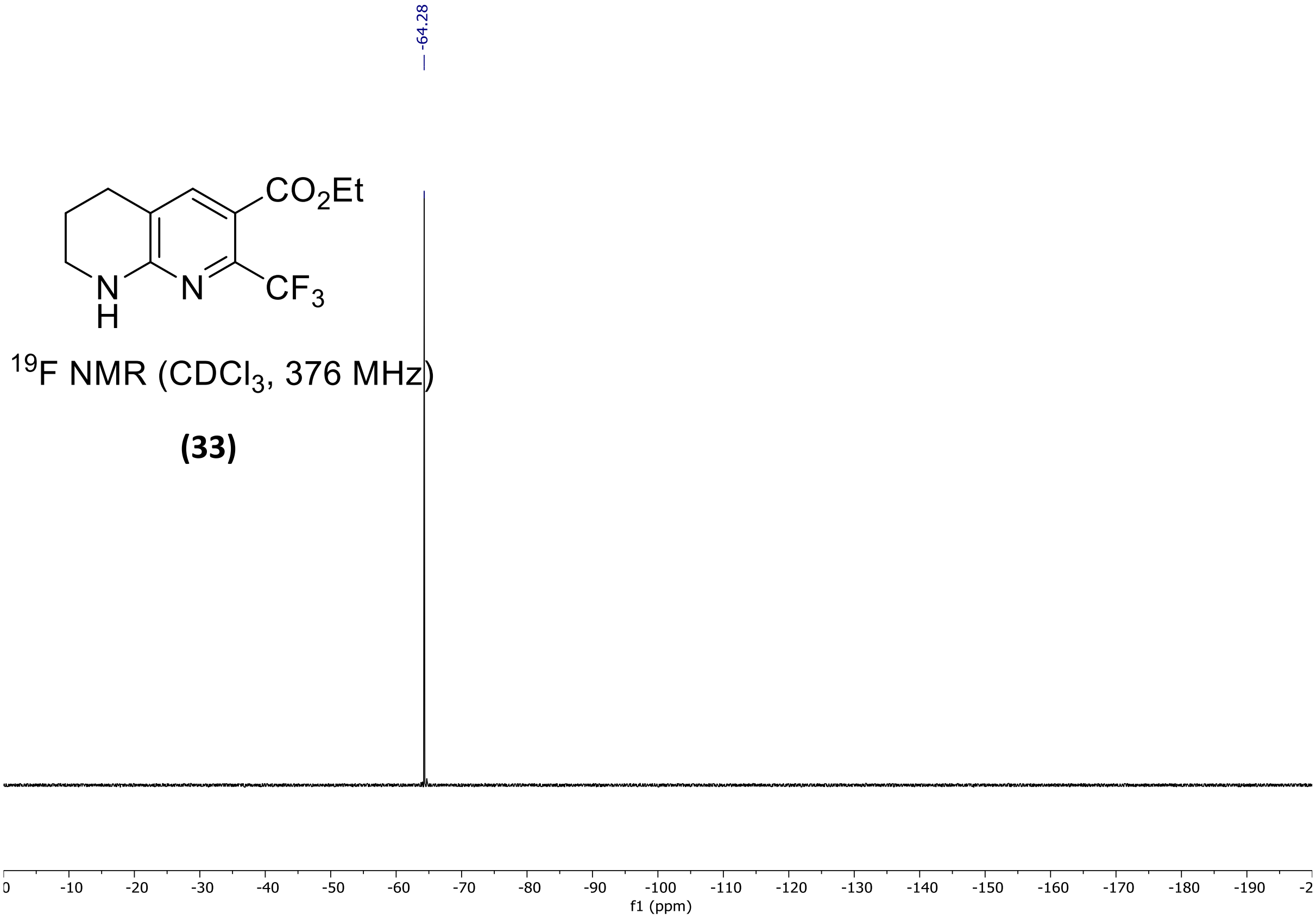

−64.28
CO_2Et
N
H
N
CF_3
^{19}F NMR ($CDCl_3$, 376 MHz)
(33)
f1 (ppm)

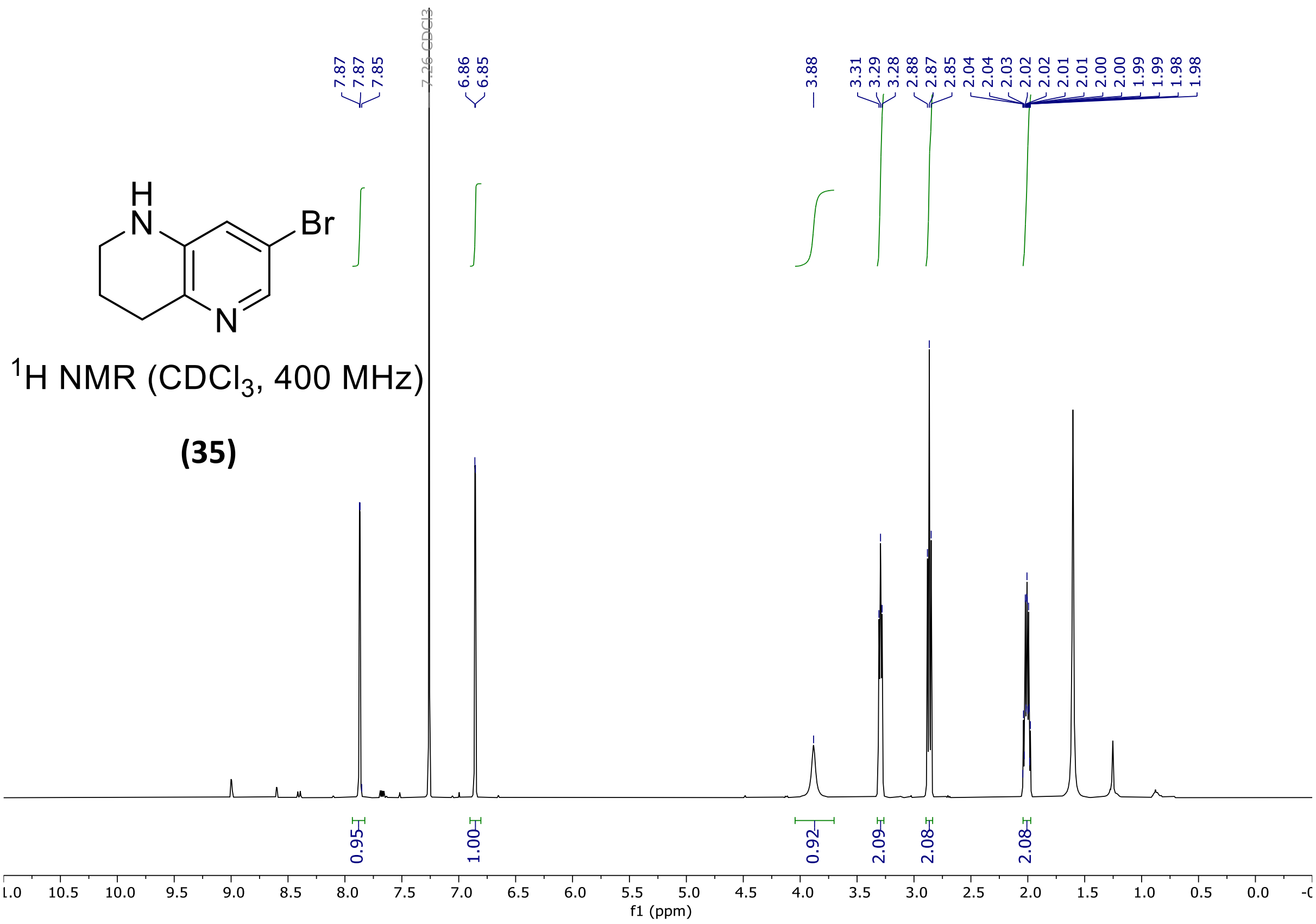

7.87
7.87
7.85
7.26 CDCl3
6.86
6.85
3.88
3.31
3.29
3.28
2.88
2.87
2.85
2.04
2.04
2.03
2.02
2.02
2.01
2.00
2.00
1.99
1.99
1.98
1.98
H
N
Br
N
^{1}H NMR ($CDCl_3$, 400 MHz)
(35)
0.95
1.00
0.92
2.09
2.08
2.08
f1 (ppm)

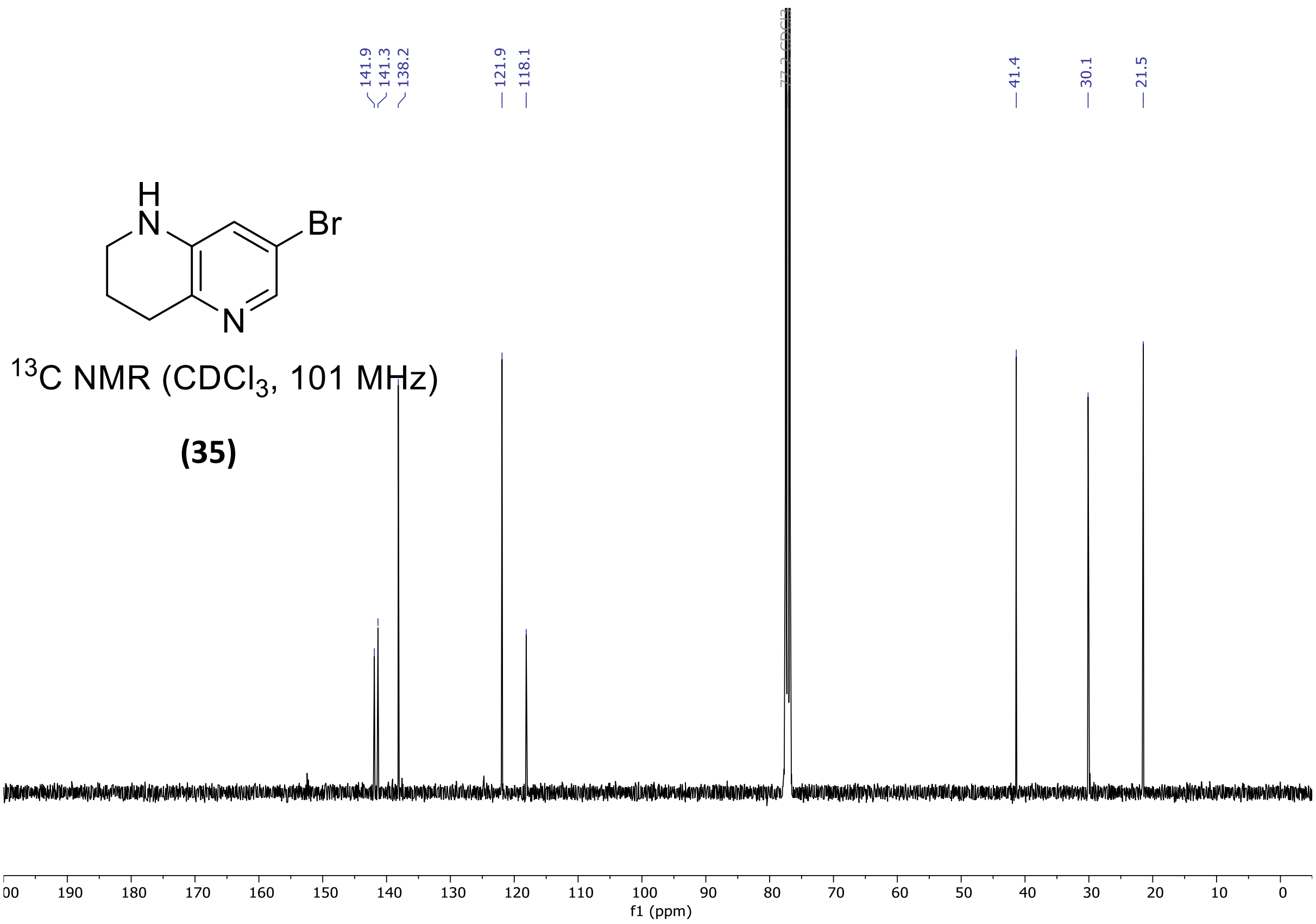

^{13}C NMR ($CDCl_3$, 101 MHz)

(35)

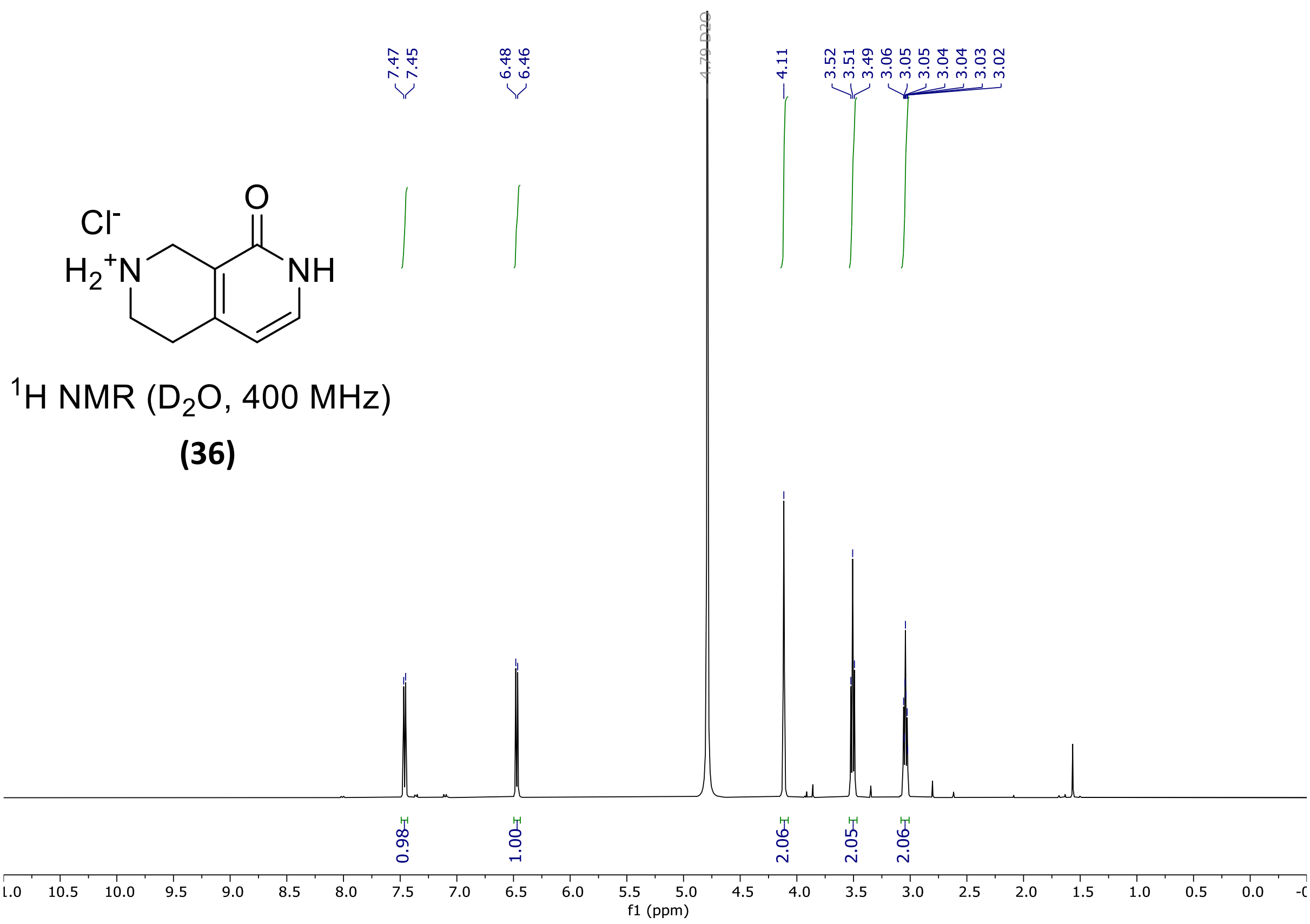

^{1}H NMR (D_2O, 400 MHz)

(36)

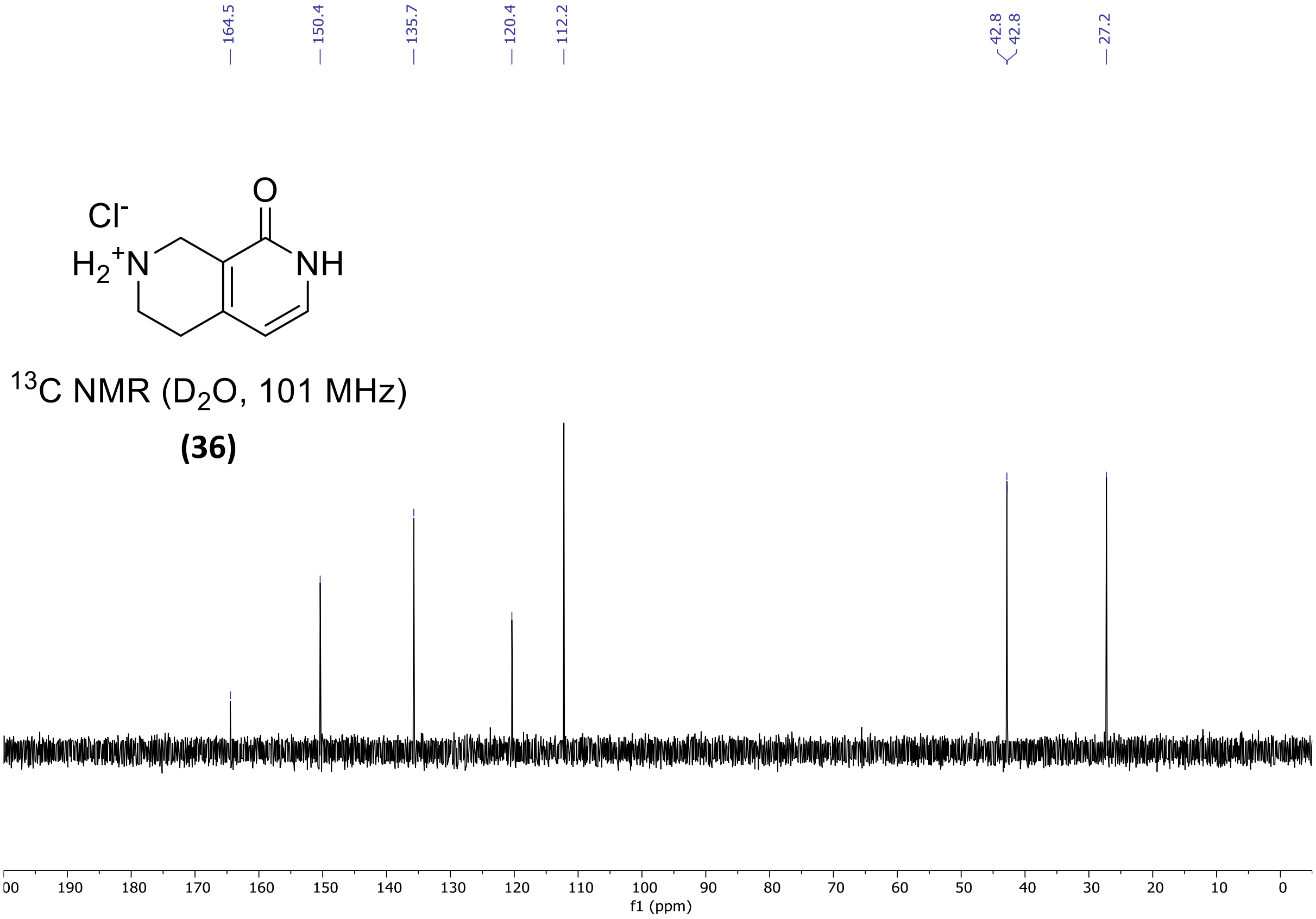

^{13}C NMR (D_2O, 101 MHz)

(36)

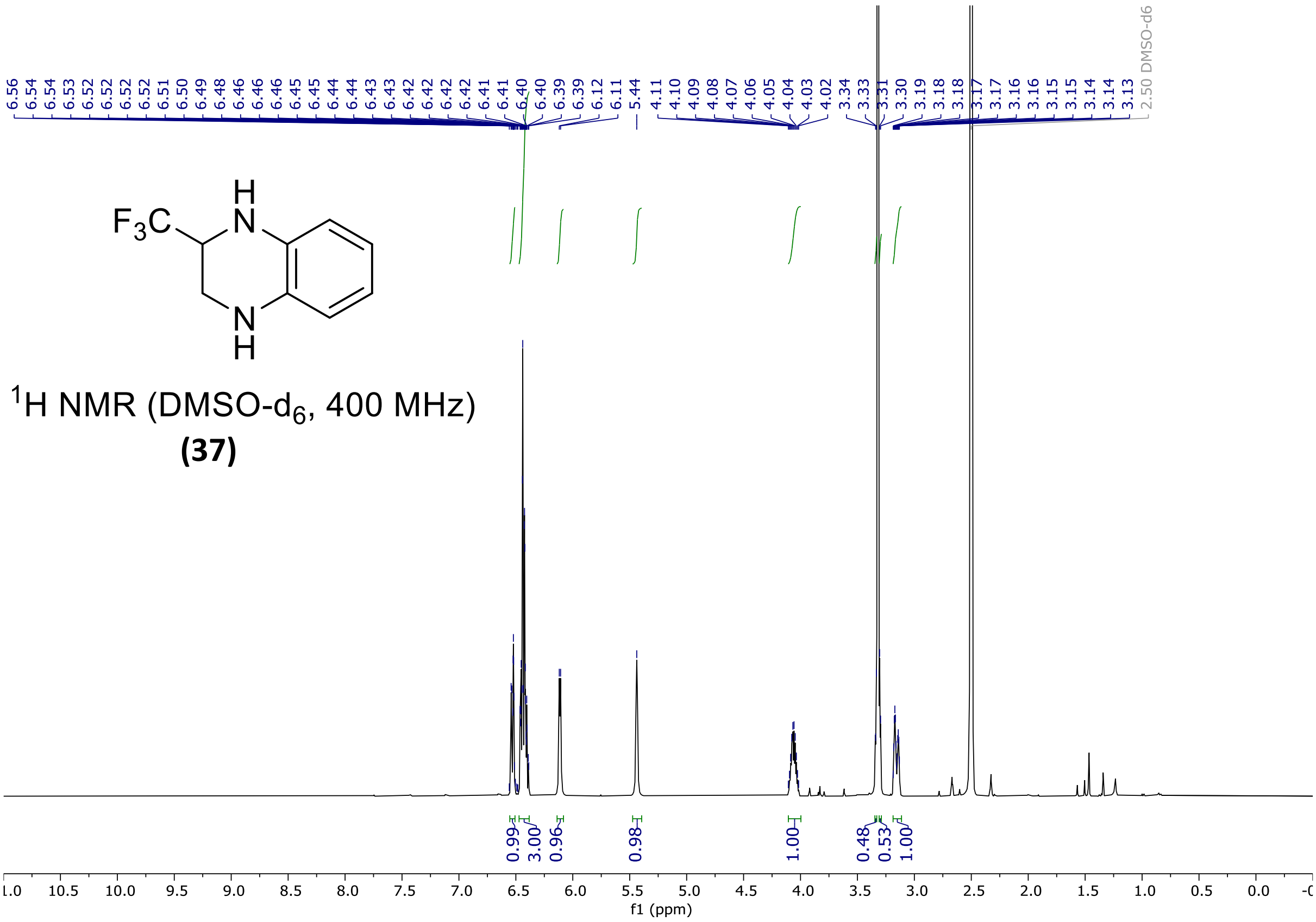

^{1}H NMR (DMSO-d_6, 400 MHz)

(37)

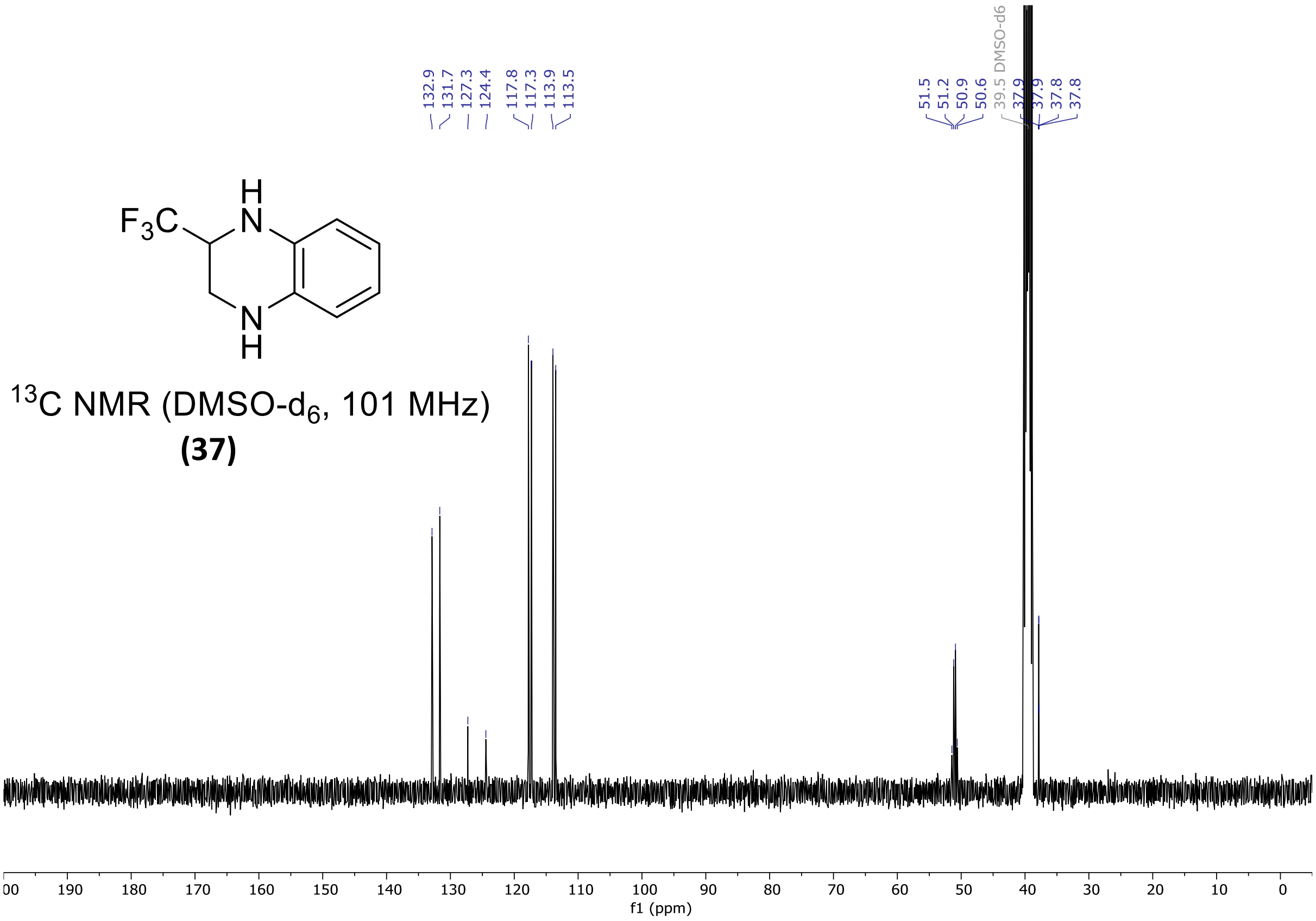

^{13}C NMR (DMSO-d_6, 101 MHz)

(37)

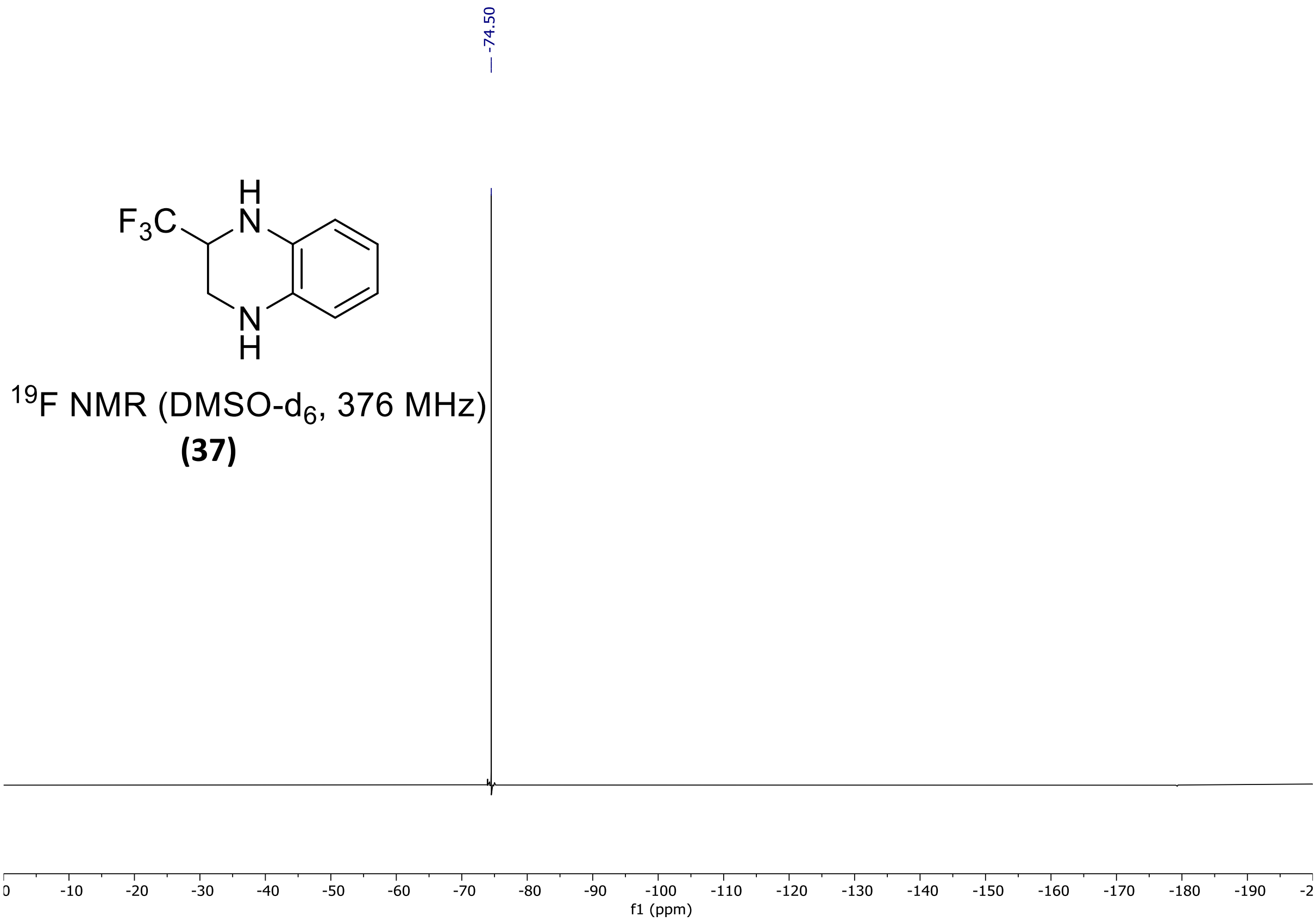

^{19}F NMR (DMSO-d_6, 376 MHz)

(37)

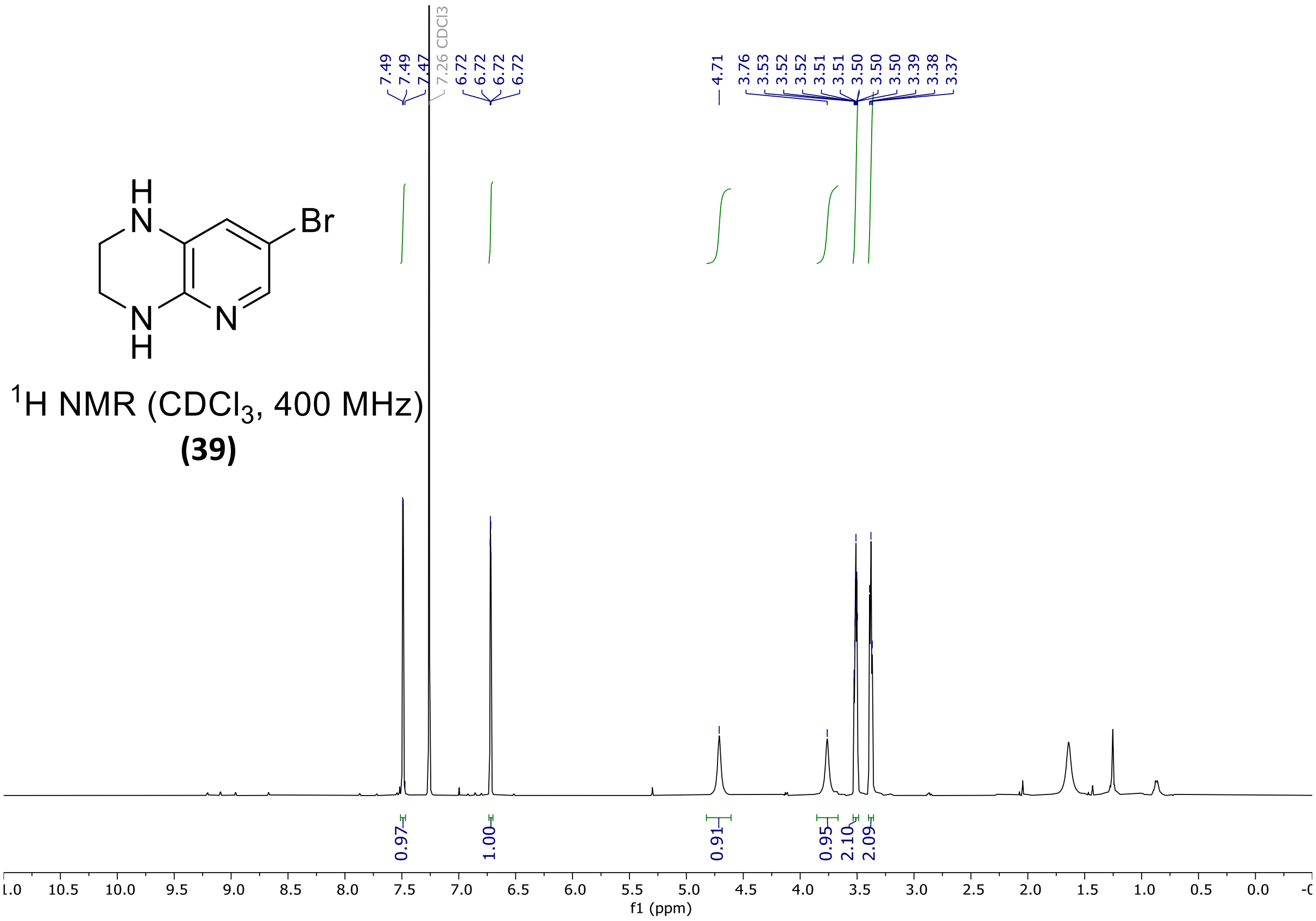

^{1}H NMR ($CDCl_3$, 400 MHz)
(39)

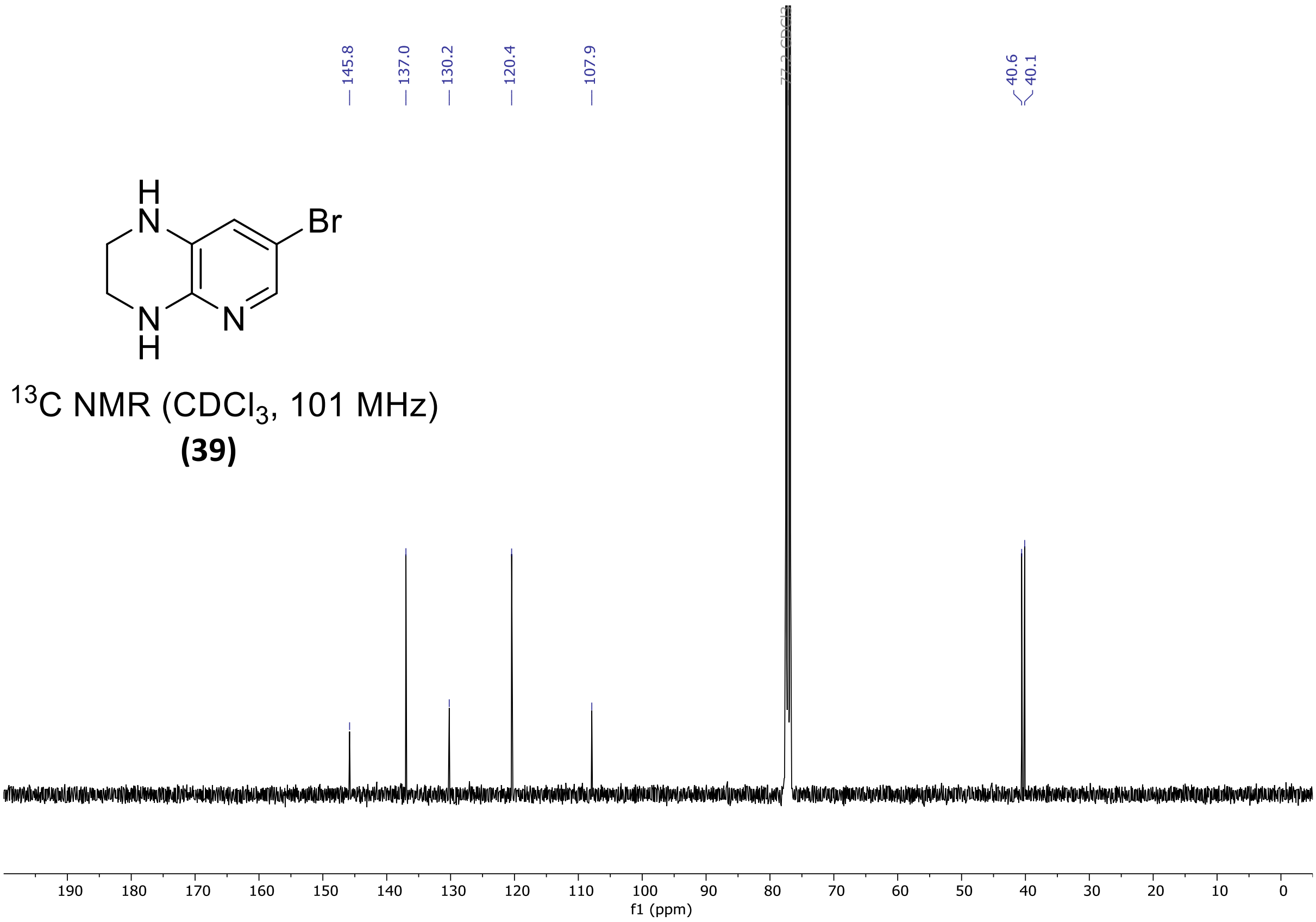

^{13}C NMR ($CDCl_3$, 101 MHz)
(39)

[6,5]-fused heteroarenes hydrogenation

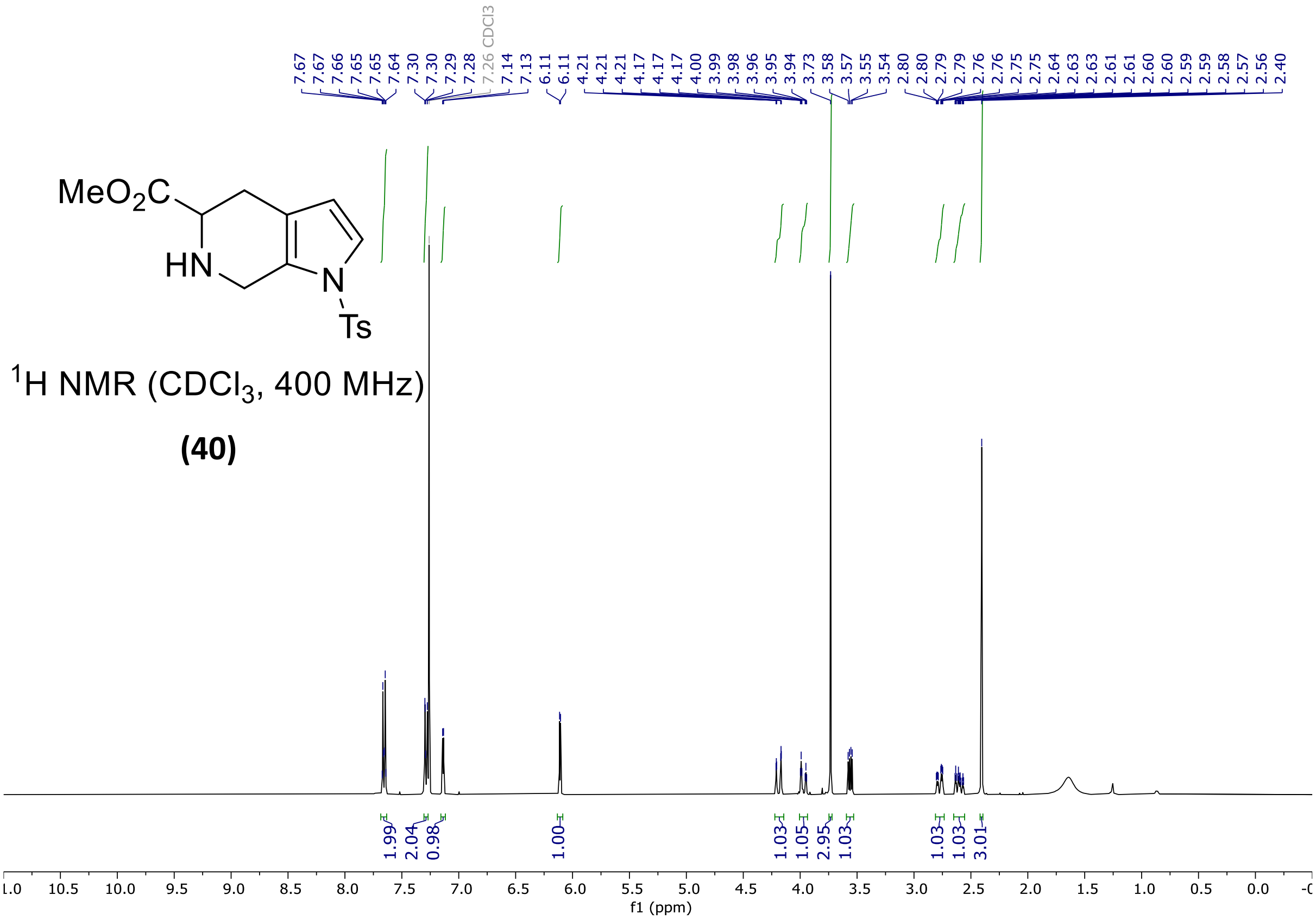

^{1}H NMR ($CDCl_3$, 400 MHz)

(40)

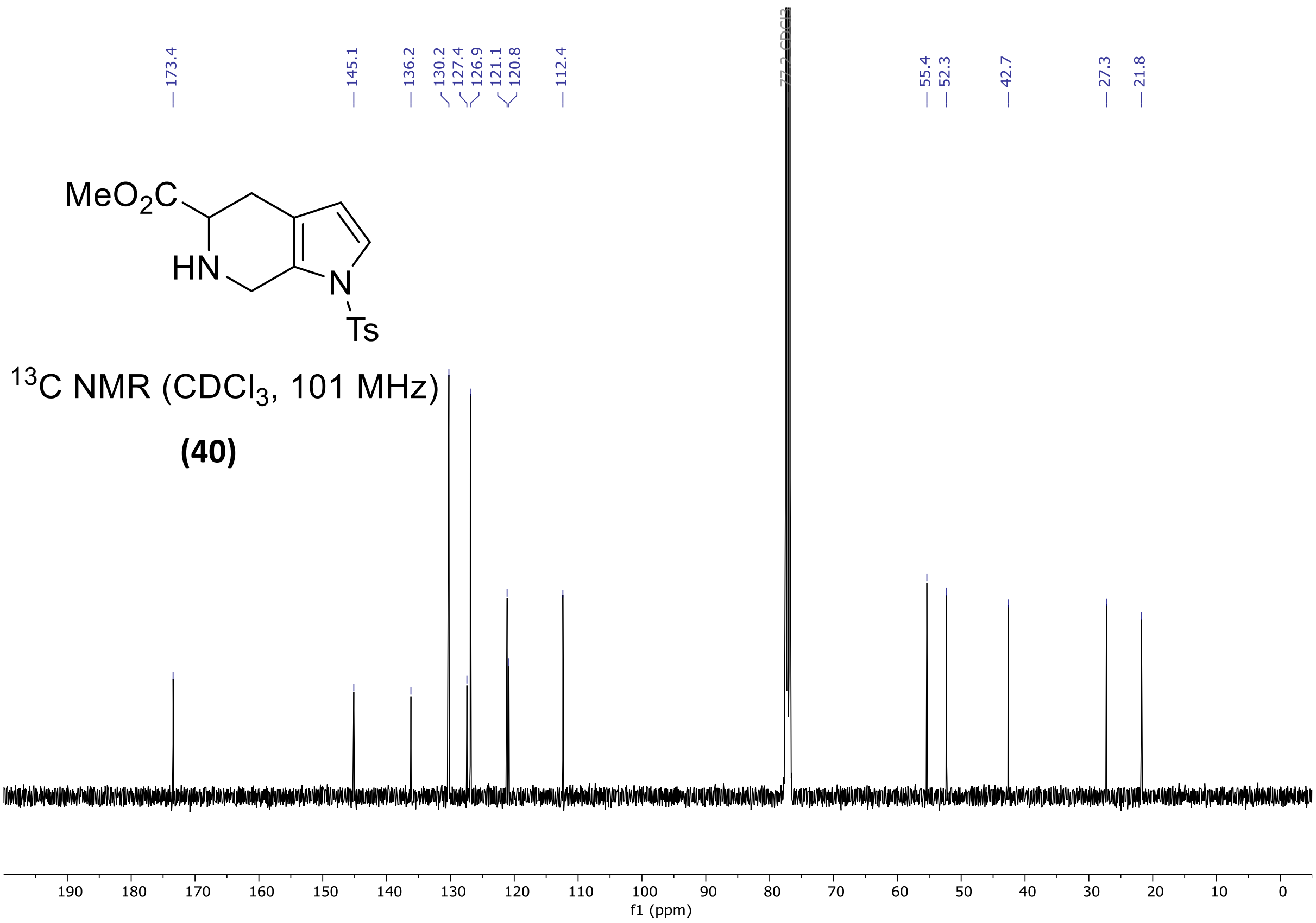

^{13}C NMR ($CDCl_3$, 101 MHz)

(40)

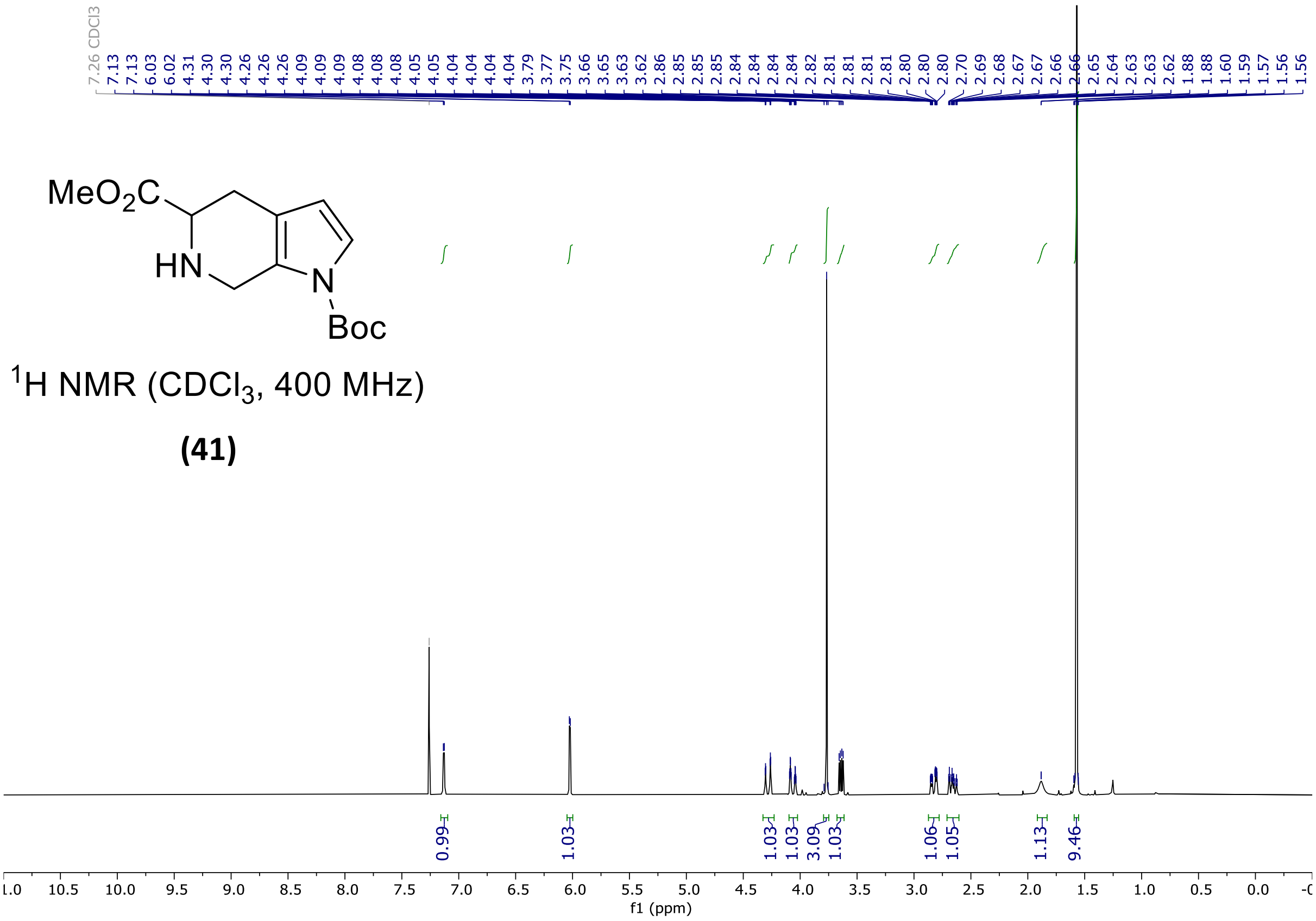

^{1}H NMR ($CDCl_3$, 400 MHz)

(41)

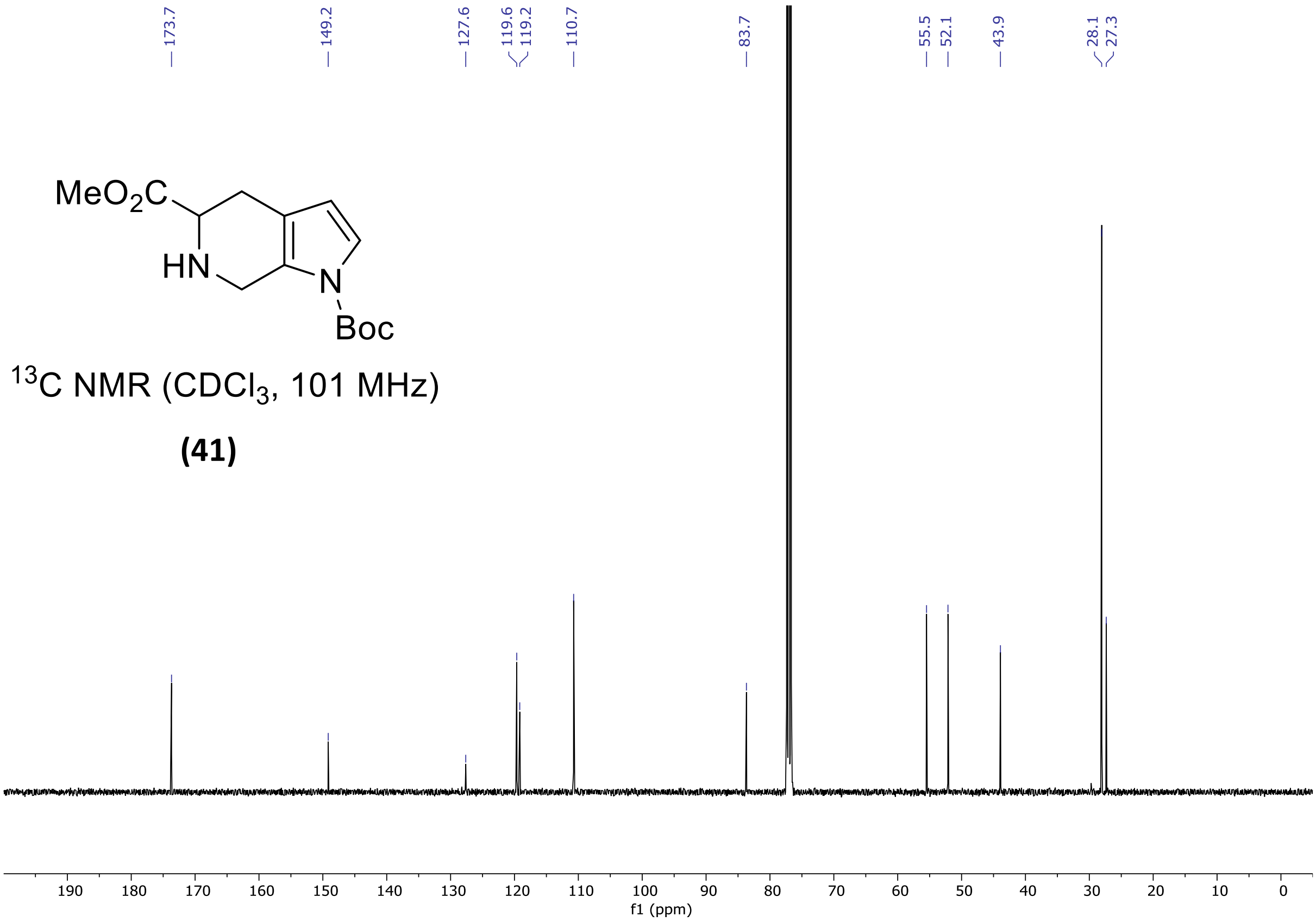

^{13}C NMR ($CDCl_3$, 101 MHz)

(41)

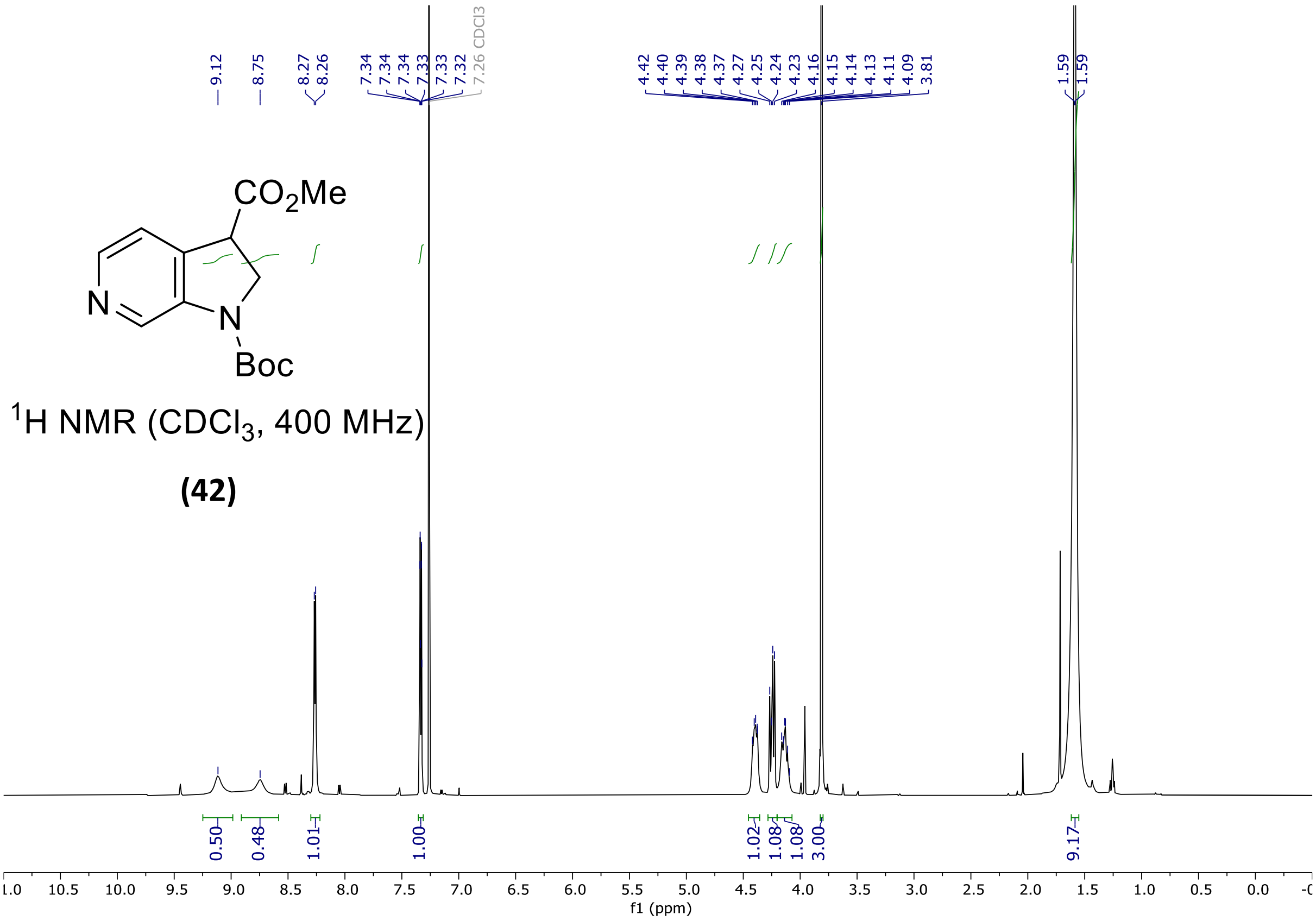

^{1}H NMR ($CDCl_3$, 400 MHz)

(42)

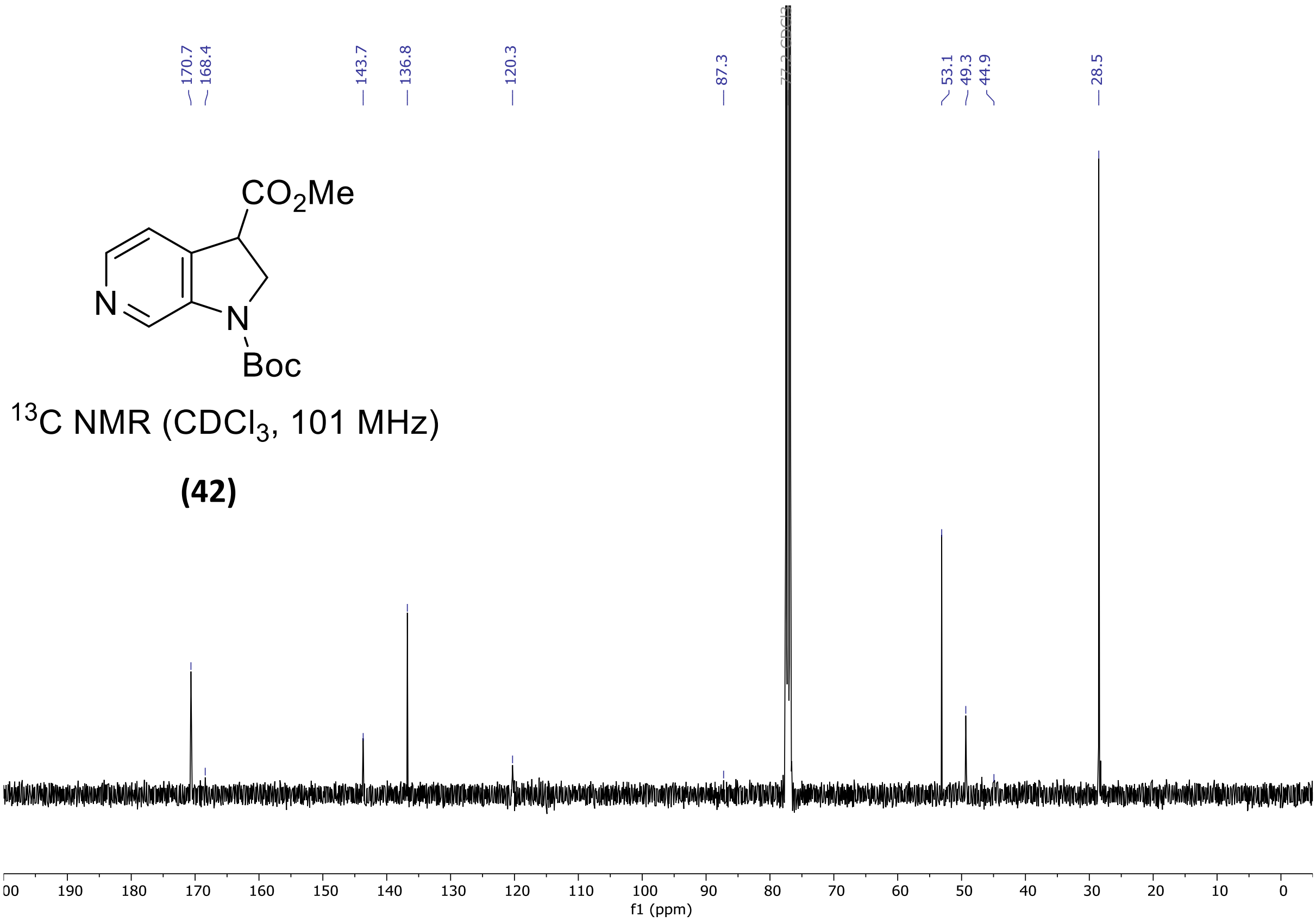

^{13}C NMR ($CDCl_3$, 101 MHz)

(42)

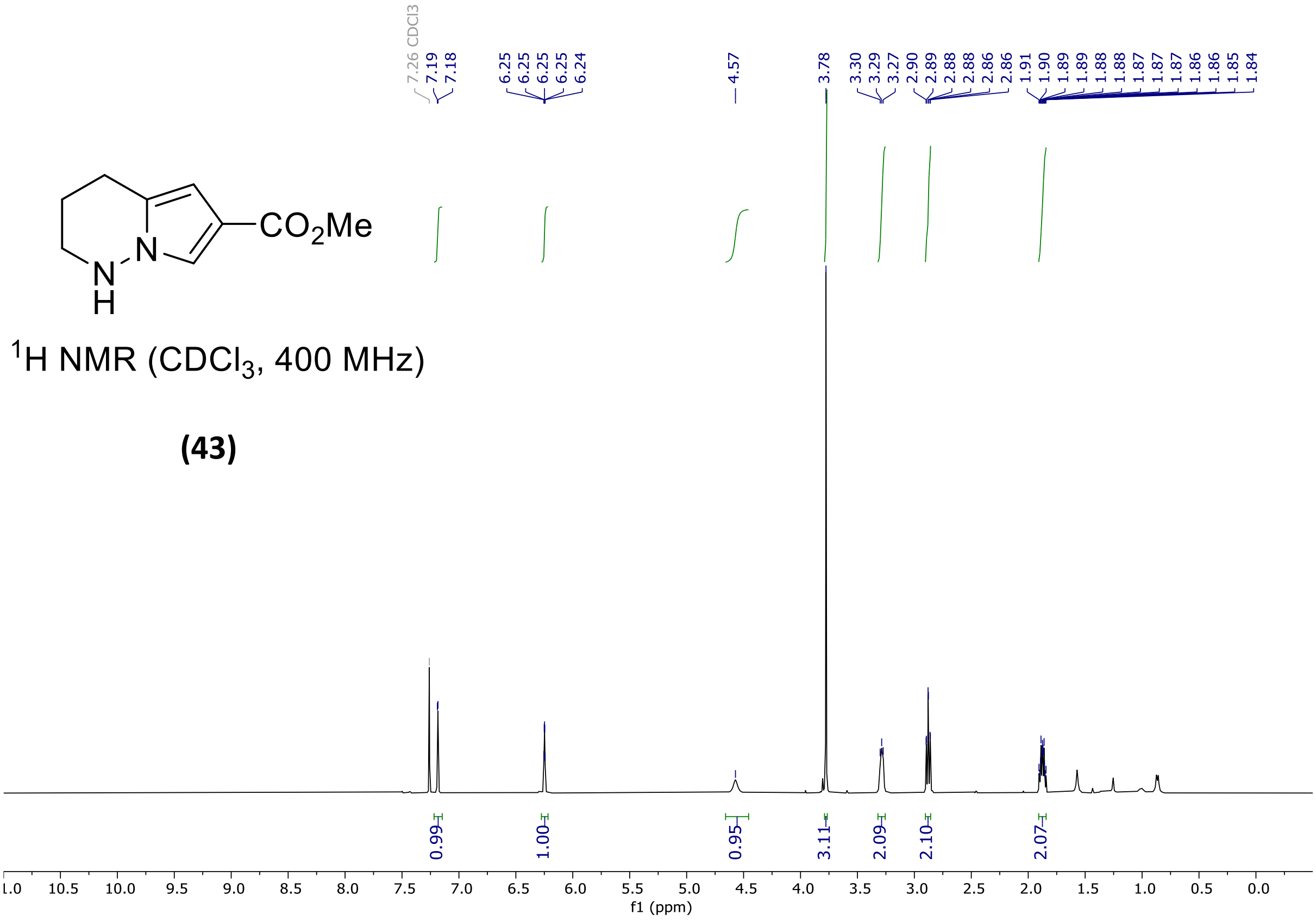

^{1}H NMR ($CDCl_3$, 400 MHz)

(43)

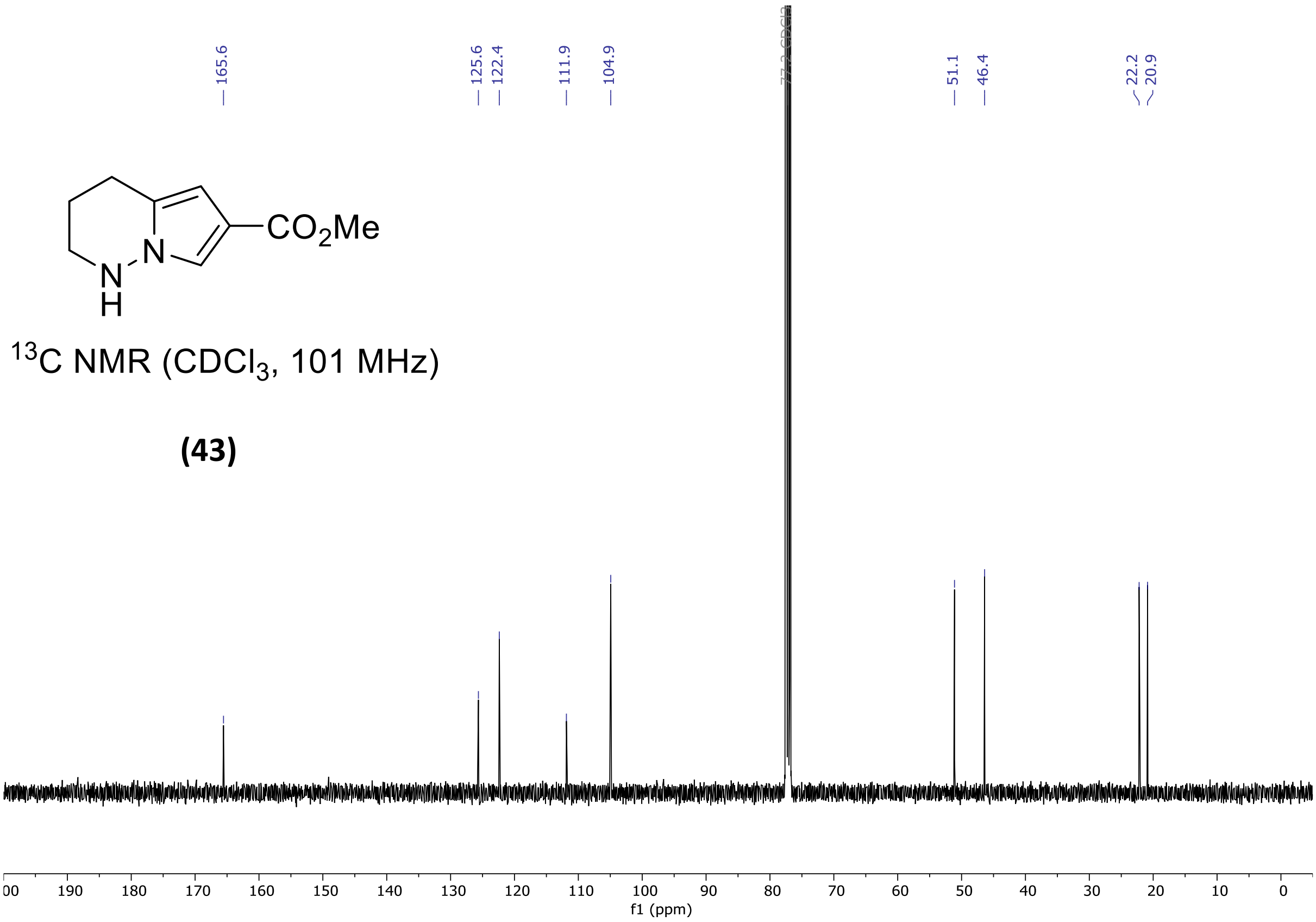

^{13}C NMR ($CDCl_3$, 101 MHz)

(43)

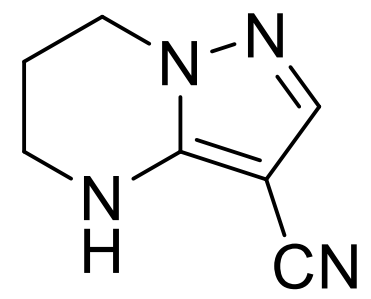

^{1}H NMR ($CDCl_3$, 400 MHz)

(44)

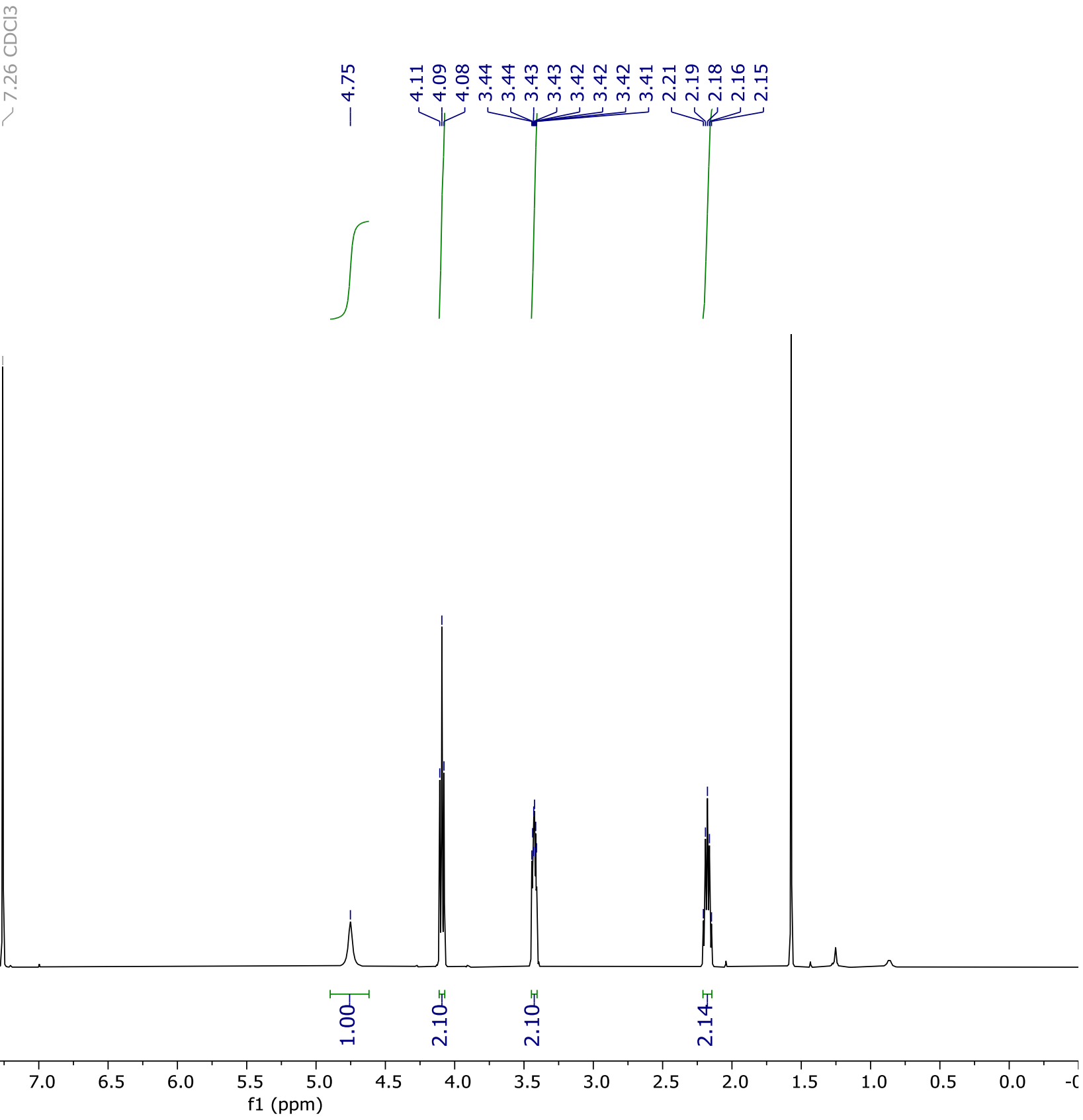

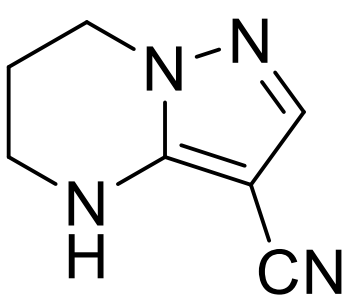

^{13}C NMR ($CDCl_3$, 101 MHz)

(44)

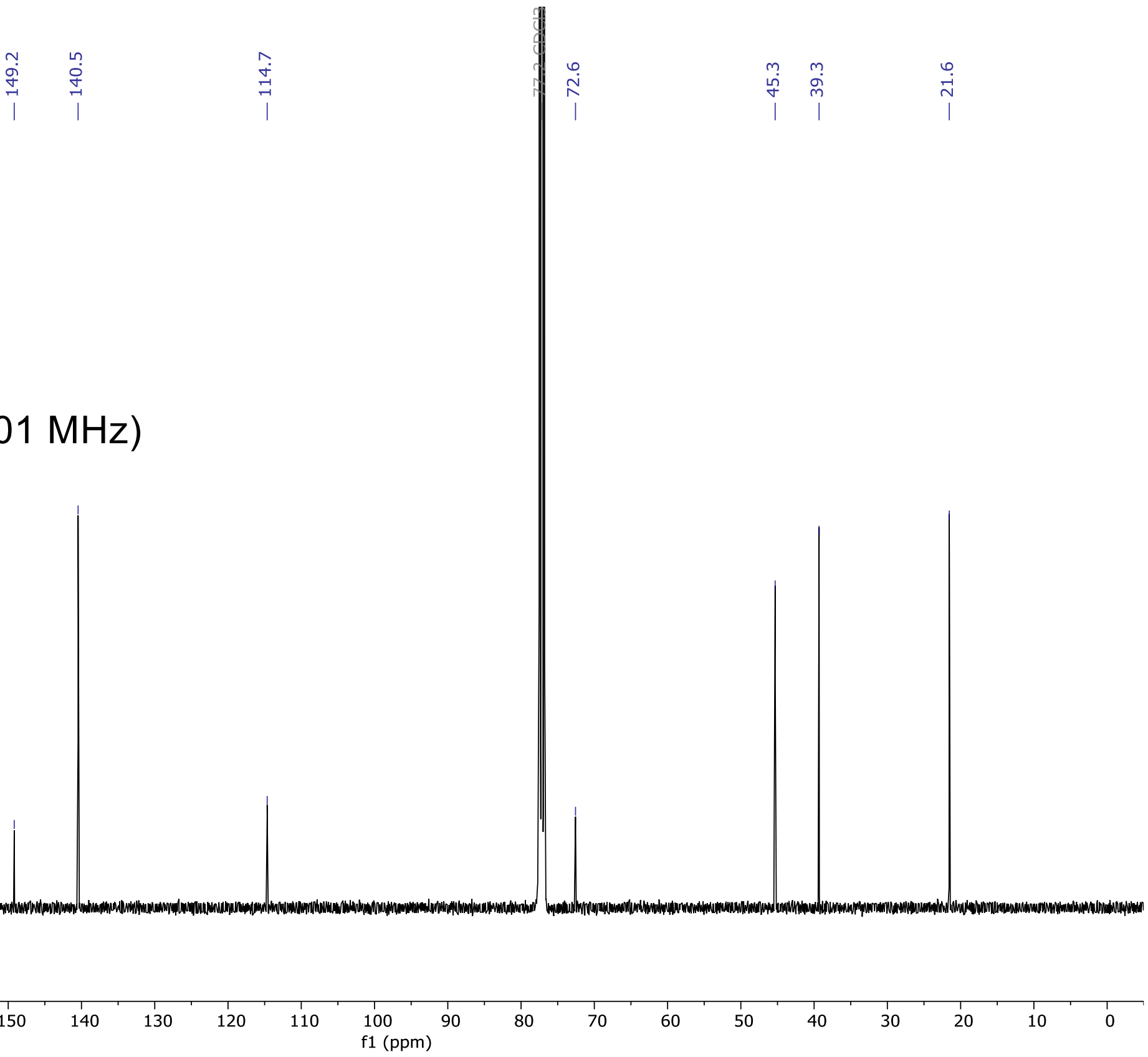

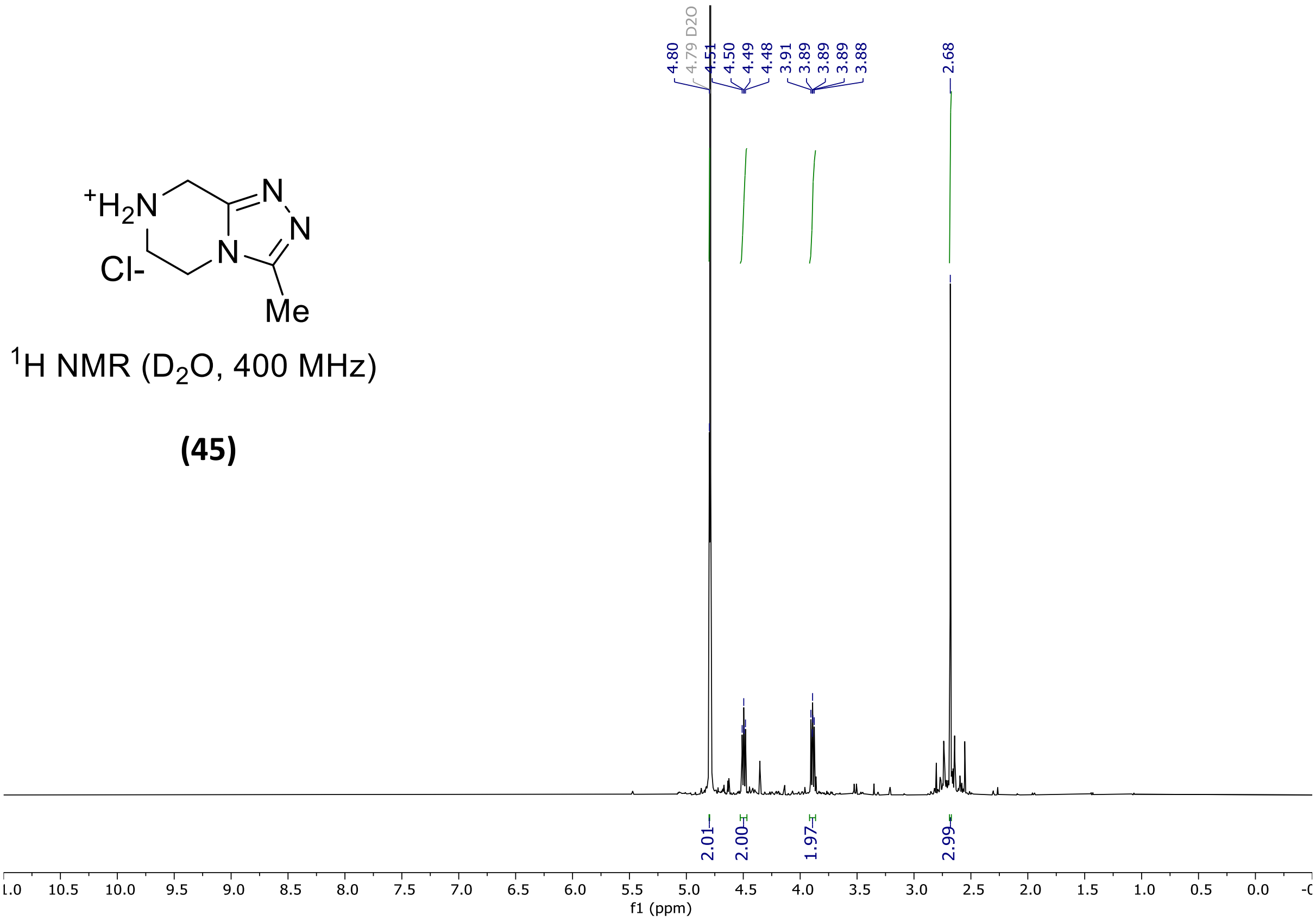

^{1}H NMR (D_2O, 400 MHz)

(45)

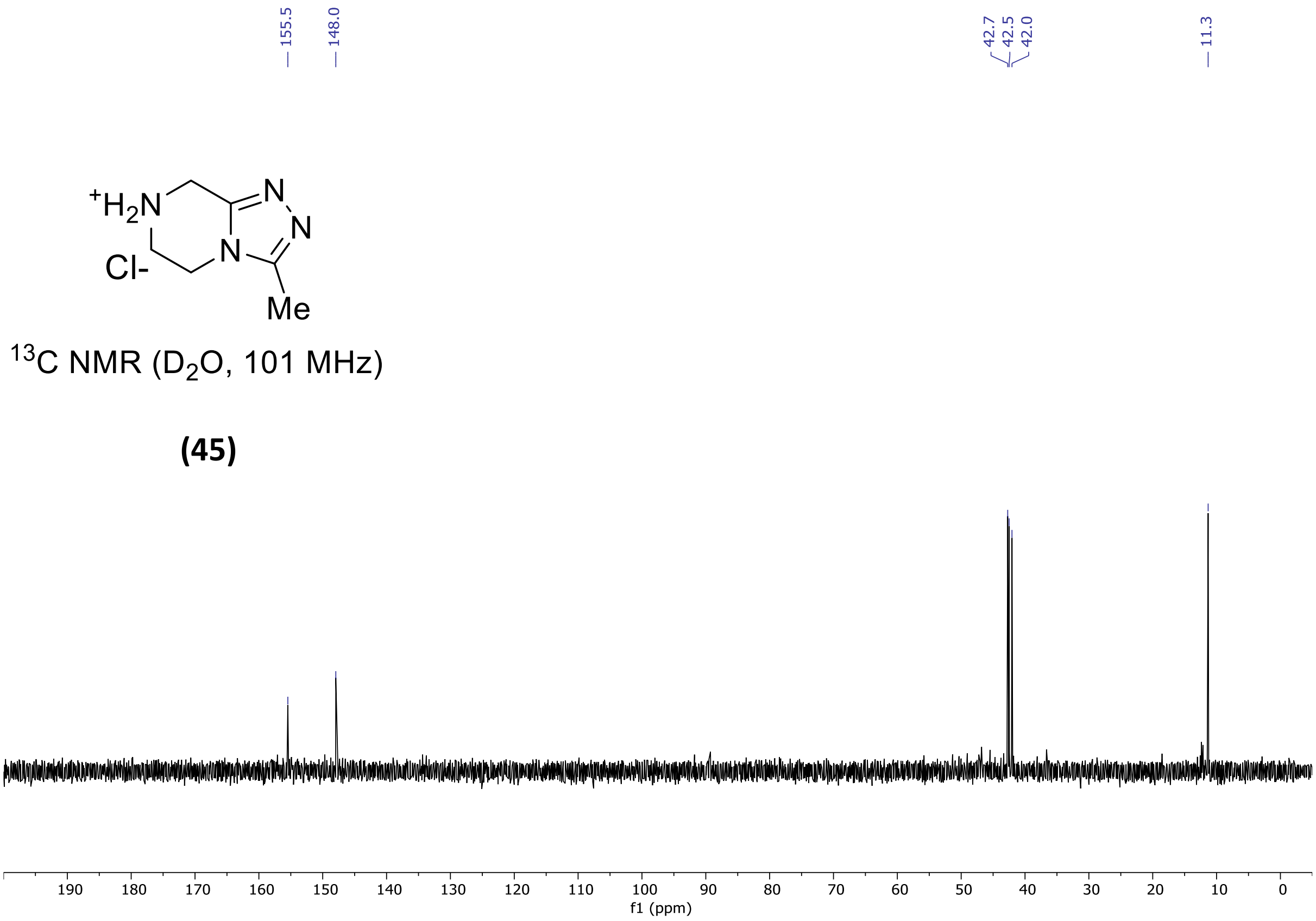

^{13}C NMR (D_2O, 101 MHz)

(45)

Supplementary references

[1] J. Mas-Roselló, T. Smejkal, N. Cramer, "Iridium-catalyzed acid-assisted asymmetric hydrogenation of oximes to hydroxylamines" *Science* **368**, (2020): 1098–1102.

[2] S. Wang, H. Lu, J. Li, D. Zou, Y. Wu, Y. Wu, "Synthesis of functionalized 3-arylpyridazines via Pd-catalyzed decarboxylative cross-coupling of pyridazine-3-carboxylic acids" *Tetrahedron Letters* **58**, (2017): 1107–1111.

[3] M. Li, Y.-P. Hsu, Y.-H. Liu, S.-M. Peng, S.-T. Liu, "Iridium complexes with ligands of 1,8-Naphthyridine-2-carboxylic acid derivatives-preparation and catalysis" *Journal of Organometallic Chemistry* **927**, (2020): 121537.

[4] G. R. Fulmer, A. J. M. Miller, N. H. Sherden, H. E. Gottlieb, A. Nudelman, B. M. Stoltz, J. E. Bercaw, K. I. Goldberg, "NMR Chemical Shifts of Trace Impurities: Common Laboratory Solvents, Organics, and Gases in Deuterated Solvents Relevant to the Organometallic Chemist" *Organometallics* **29**, (2010): 2176–2179.

[5] R. K. Harris, E. D. Becker, S. M. Cabral de Menezes, P. Granger, R. E. Hoffman, K. W. Zilm, "Further conventions for NMR shielding and chemical shifts (IUPAC Recommendations 2008)" *Pure and Applied Chemistry* **80**, (2008): 59–84.

[6] G. Lunn, "Reduction of heterocycles with nickel-aluminum alloy" *The Journal of Organic Chemistry* **52**, (1987): 1043–1046.

[7] Y. Nishimura, Y. Yasui, S. Kobayashi, M. Yamaguchi, H. Cho, "Synthesis of novel 2-phenyl-5-substituted dihydropyrimidines using 2-phenyl-1,3-diaza-1,3-butadienes and electron-deficient olefins" *Tetrahedron* **68**, (2012): 3342–3350.

[8] A. Lebrêne, T. Martzel, L. Gouriou, M. Sanselme, V. Levacher, S. Oudeyer, C. Afonso, C. Loutelier-Bourhis, J.-F. Brière, "The Catalytic Regio- and Stereoselective Synthesis of 1,6-Diazabicyclo[4.3.0]nonane-2,7-diones" *The Journal of Organic Chemistry* **86**, (2021): 8600–8609.

[9] D. Timelthaler, C. Topf, "Heterogeneous Hydrogenation of Quinoline Derivatives Effected by a Granular Cobalt Catalyst" *Synthesis* **54**, (2022): 629–642.

[10] D. Bhattacharyya, S. Nandi, P. Adhikari, B. K. Sarmah, M. Konwar, A. Das, "Boric acid catalyzed chemoselective reduction of quinolines" *Organic and Biomolecular Chemistry* **18**, (2020): 1214–1220.

[11] J. Zhang, Z. Chen, M. Chen, Q. Zhou, R. Zhou, W. Wang, Y. Shao, F. Zhang, "Lanthanide/B(C6F5)3-Promoted Hydroboration Reduction of Indoles and Quinolines with Pinacolborane" *Journal of Organic Chemistry* **89**, (2024): 887–897.

[12] Z. Zhu, J. Furr, J. K. Buolamwini, "Synthesis and Flow Cytometric Evaluation of Novel 1,2,3,4-Tetrahydroisoquinoline Conformationally Constrained Analogues of Nitrobenzylmercaptopurine Riboside (NBMPR) Designed for Probing Its Conformation When Bound to the *e s* Nucleoside Transporter" *Journal of Medicinal Chemistry* **46**, (2003): 831–837.

[13] Z. Wei, H. Li, Y. Wang, Q. Liu, "A Tailored Versatile and Efficient NHC-Based NNC-Pincer Manganese Catalyst for Hydrogenation of Polar Unsaturated Compounds" *Angewandte Chemie - International Edition* **62**, (2023), DOI 10.1002/anie.202301042.

[14] B. Xiong, Y. Li, W. Lv, Z. Tan, H. Jiang, M. Zhang, “Ruthenium-Catalyzed Straightforward Synthesis of 1,2,3,4-Tetrahydronaphthyridines via Selective Transfer Hydrogenation of Pyridyl Ring with Alcohols” *Organic Letters* **17**, (2015): 4054–4057.

[15] B. Xiong, S. Di Zhang, L. Chen, B. Li, H. F. Jiang, M. Zhang, “An annulative transfer hydrogenation strategy enables straightforward access to tetrahydro fused-pyrazine derivatives” *Chemical Communications* **52**, (2016): 10636–10639.

[16] O. V. Borysov, V. V. Voloshchuk, M. A. Nechayev, V. A. Lysenko, M. M. Nikolaychuk, A. O. Portiankin, O. V. Oliinyk, D. A. Lega, D. M. Volochnyuk, S. V. Ryabukhin, “Focused small molecule library of 5,6,7,8-tetrahydro[1,2,4]triazolo-[4,3-a]pyrazines: a brick for the house of medicinal chemistry” *Chemistry of Heterocyclic Compounds* **59**, (2023): 429–441.
